# To Supplement or Not to Supplement: A Metabolic Network Framework for Human Nutritional Supplements

**DOI:** 10.1371/journal.pone.0068751

**Published:** 2013-08-05

**Authors:** Christopher D. Nogiec, Simon Kasif

**Affiliations:** 1 Bioinformatics Program, Boston University, Boston, Massachusetts, United States of America; 2 Joslin Diabetes Center, Boston, Massachusetts, United States of America; 3 Department of Bioengineering, Boston University, Boston, Massachusetts, United States of America; 4 Children's Hospital Informatics Program, Harvard-MIT Division of Health Sciences and Technology (CHIP@HST), Boston, Massachusetts, United States of America; Mayo Clinic, United States of America

## Abstract

Flux balance analysis and constraint based modeling have been successfully used in the past to elucidate the metabolism of single cellular organisms. However, limited work has been done with multicellular organisms and even less with humans. The focus of this paper is to present a novel use of this technique by investigating human nutrition, a challenging field of study. Specifically, we present a steady state constraint based model of skeletal muscle tissue to investigate amino acid supplementation's effect on protein synthesis. We implement several *in silico* supplementation strategies to study whether amino acid supplementation might be beneficial for increasing muscle contractile protein synthesis. Concurrent with published data on amino acid supplementation's effect on protein synthesis in a post resistance exercise state, our results suggest that increasing bioavailability of methionine, arginine, and the branched-chain amino acids can increase the flux of contractile protein synthesis. The study also suggests that a common commercial supplement, glutamine, is not an effective supplement in the context of increasing protein synthesis and thus, muscle mass. Similar to any study in a model organism, the computational modeling of this research has some limitations. Thus, this paper introduces the prospect of using systems biology as a framework to formally investigate how supplementation and nutrition can affect human metabolism and physiology.

## Introduction

The Nutrition Business Journal (2010) estimates the supplement industry at $23.7 billion [1]. However, the claims and efficacy of these supplements are not routinely regulated by the Federal Drug Administration. The question of whether to supplement and what to supplement to increase muscle mass has given rise to many studies over the years [2], [3], [4]. Much focus has been on protein and amino acid supplementation. However, human studies can be difficult due to small sample sizes, length of time per study, and only a limited number of variables can be investigated at a time (i.e., one amino acid or a few combinations at a time). Although some results show clear evidence for the efficacy of certain amino acids at increasing muscle mass [2] others still show inconclusive results [3], [4].

One goal of supplementation research in exercise physiology, fitness, and health is to determine which metabolites have the biggest effect on increasing muscle protein synthesis during the recovery phase. One method has been to increase the bioavailability of amino acids, as these are the building blocks of muscle proteins. Some amino acids and combinations have been investigated in great detail. Branched-chain amino acids (BCAA's) have been shown to increase protein synthesis, specifically in muscle tissue, in response to resistance exercise [2]. Of the three BCAA's, leucine seems to have the biggest effect, and one reason could be that leucine may signal an increase in enzymes involved in protein synthesis [2]. In addition to the BCAA's, some amino acids have been investigated with mixed results. For example, supplementing with arginine, a conditionally essential amino acid, shows that it alone does not seem to increase protein synthesis nor does it increase protein synthesis in the presence of the eight essential amino acids. However, some studies indicate that arginine can increase protein synthesis in conjunction with other amino acids or with meals [3]. However, it is unclear which combination of amino acids is most beneficial. Furthermore, many studies show no effect of some amino acids, but these amino acids are still highly promoted among nutrition and supplement companies. Glutamine is promoted by supplement manufacturers for numerous positive effects, one of which helps muscle protein synthesis. However, a recent review suggests that glutamine supplementation does not provide any of these benefits [4].

It is challenging to determine the individual effects of supplements and their combinations in human studies. Also, without trying every combination, it is unclear which are the most beneficial and whether there is a specific combination that is most effective. Another question is whether increasing the availability of certain amino acids can increase protein synthesis alone or if certain amino acids affect gene and/or signaling, which in turn can affect amino acid synthesis through gene activation.

The advent of metabolic modeling of human tissues opens the door to using this platform for studying multiple hypotheses in this space including nutrition and supplementation. Multiple regimes of supplementation can be investigated efficiently and predictively. Constraint based models convert a network of reactions and their stoichiometry into a matrix (**S**) where the columns is the set of reactions and the rows is the set of metabolites. We can set constraints to these reactions based on thermodynamics of the reactions and substrate availability (**v**) to make the system biologically relevant and to limit the resultant space. Each constraint captures a linear relationship between input and output fluxes for each reaction. We investigate steady state scenarios and use the model to predict optimal flux distributions (**v**), under specific optimization parameters: S * v = 0, where **S** is the matrix of the stoichiometry of the reactions, and **v** is the set of fluxes through reactions.

In addition to the basic set of linear equalities and inequalities that constrain the functional relationship of input/outputs to any reactions, we also postulate a linear optimization criterion that attempt to maximize a desired behavior. In several highly successful unicellular models researchers maximized growth rate of a bacterium under different conditions and environmental constraints [5]. Other research maximized ATP flux. In addition one can simulate perturbation in the form of different nutrient states, knockouts of reactions (e.g. in response to inhibitors) and predict the resulting effects on the chosen flux before and after perturbations. In this paper we introduce perturbation in the form of additional supplementation and measure the effect of individual and combinations of supplements perturbations on protein synthesis flux in the model.

For a comprehensive review of FBA see Oberhardt [5], [6], [7]. While there has been an increased attempt to create human tissue specific models [8], [9], [10], [11], some a subset of the human metabolic model [12], these models are not curated, and the skeletal muscle tissue models have not specifically been reported on [9], [11]. While our model is smaller than previously published skeletal muscle models, it was built from a previously published model [13] and expanded by identifying key reactions in muscle tissue, similar to the method used by Cakir [8]. This curation process is different than methods used by Shlomi [9] and Agren [11], and it includes detailed fluxes for specific muscle proteins (the contractile complexes for Type 1, 2a, 2x, and 2b muscle tissue) while the larger models do not have these specific reactions.

## Methods

### Model Development

The flux model developed here combines the previously published flux model of the mitochondria [13] with muscle specific metabolism including amino acid metabolism and protein synthesis. The goal was to capture metabolism from the major macronutrients: carbohydrates, lipids, and protein/amino acids. In addition, beyond major catabolic reactions, amino acid biosynthesis and protein synthesis for the major contractile muscle proteins has been included. The network includes 374 reactions and 341 metabolites (Files S2 and S3). The model has a rank of 315 and 26 degrees of freedom.

Some changes were made to the Ramakrishna model [13] to make it more complex and detailed so that the model can be expanded. For example, Aconitase was added (EC 4.2.1.3) which catalyzed the reversible reaction between isocitrate and citrate. In addition, the entire electron transport complex was modeled, including electron transfer flavoprotein-ubiquinone oxidoreductase which oxidizes flavin adenine dinucleotide (EC 1.5.5.1). This gives a more accurate ATP count. Further modifications were made to fatty acid oxidation. The fatty acid used by Ramakrishna [13], palmitate, is a 16 carbon fatty acid. Although the same enzymes are used in each cycle of the fatty acid oxidation, each cycle here is detailed. In other words, there are reactions which oxidize a 14 carbon fatty acid, a 12 carbon fatty acid, etc. This makes it possible to add other fatty acids and to incorporate amino acid oxidation into the same cycle.

The model was also expanded beyond the mitochondria model to include amino acid and protein metabolism. During certain conditions, the muscle will supplement or convert to amino acid oxidation for energy supply [14]. In addition, including protein synthesis may help elucidate optimal nutrient supplies to build muscle. Amino acids, which are known to be metabolized in muscles were also included. The branched chain amino acids (BCAA), isoleucine, leucine, and valine, are used as an energy supply during certain conditions, and the byproducts enter fatty acid oxidation cycle and TCA cycle. In addition to the BCAA's, serine can be converted to pyruvate (EC 4.3.1.17), which can be used in the glycolytic pathway. The oxidative products of lysine also enter the fatty acid oxidation pathway. Proline oxidation produces fadh2, which can be incorporated into the ETC. The enzymes, which catalyzes these reactions were found in the *Homo sapiens* KEGG pathway map. Expression of the enzymes was confirmed using Uniprot information on tissue specificity for each of these enzymes involved in amino acid metabolism. In addition to amino acid oxidation, there is some amino acid biosynthesis.

Additional, amino acid metabolism components are included in the protein synthesis pathways. The major contractile proteins actin, myosin, tropomyosin, and troponin were identified. Their amino acid make-up was determined from NCBI database. Different muscle tissue type expresses different myosin, topomyosin and troponin. The myosin complex is made of a heavy chain, which differs between type 1/2x, 2a, and 2b muscle types, and a light chain kinase, and a light chain phosphorylated. Tropomyosin is expressed as two sub-types, type 1 and type 2, and form dimmers within the respective tissue type. Troponin is composed of three subtypes specific to each muscle tissue type 1 or 2: troponin C, troponin I, and troponin T. The entire contractile protein complex is composed in the ratio of 7 actin:7 myosin:1 tropomyosin dimer: 1 troponin complex [15]. See Table S1 for details.

### The Implementation of the Model

Once all the reactions were identified and collected, the text was converted into a matrix for use in Mat Lab software package (Version 7.8.0.347 64 bit). The optimization software was GLPK v4.39, using the default linear programming settings maximizing for the objective function (contractile protein complex, see above). We took a simple approach and did not use any other optimization techniques.

The model is described in standard SBML syntax and included in File S3.

### Assumptions

In addition to assumptions made with FBA [5], we add three postulates. First, we assume muscle tissue is in a recovery state and primed for contractile protein synthesis. For example, we assume a supplementation strategy after exercise stimulus, and all the hormones and enzymes available to support protein synthesis are available. Thus, we are investigating an acute response to supplementation. Next, because of the difficulty in obtaining amino acid uptake fluxes for all amino acids into muscle tissue, we assume the uptake flux is proportional to amino acid concentration levels, as levels are maintained by the help of the liver and red blood cells [16], [17]. Last, we assume the system reaches a steady state over enough time to exhaust the concentrations available in the plasma levels, as we are seeking qualitative results with our exploratory study.

### Experimental Method

The goal of the experiment is to determine if supplementation of amino acids above a normal, fasting state can improve the protein synthesis flux. The normal fasting amino acid profile is based on Cynober [16] (Table 1). The post-absorptive (2 hours post ingestion of a 200 g sirloin steak) is based on the average of Aoki [18], Tessari [19], and Pozefsky [20] (Table 1).

**Table 1:** Fasting and Post-absorptive control for glucose, fatty acid and amino acid levels.

Metabolite	Fasting (mmol/L)^1^	Post-absorptive(mmol/L)^2^
Glucose	5	7
Palmitate	0.125	0.125
Tetradecanoate	0.230	0.230
Arginine	0.080±20	0.115±14
Histidine	0.082±10	0.118±7
Isoleucine	0.062±14	0.128±14
Leucine	0.123±25	0.271±14
Lysine	0.188±32	0.294±24
Methionine	0.025±4	0.030±2
Phenylalanine	0.057±9	0.068±4
Threonine	0.140±33	0.182±13
Tryptophan	0.044±7	0.036±3.6
Valine	0.233±43	0.349±36
Alanine	0.333±74	0.309±46
Asparagine	0.041±10	0.045±2
Aspartate	0.003±1	0.067±10
Cysteine	0.052±11	0.093±10
Glutamate	0.024±15	0.152±22
Glutamine	0.586±84	0.541±25
Glycine	0.230±52	0.281±33
Proline	0.168±60	0.299±23
Serine	0.114±19	0.175±11
Tyrosine	0.059±12	0.092±9

1. Cynober [16].

2. Average of levels from Aoki [18], Tessari [19], and Posefsky [20].

Supplementation was modeled by changing the available flux for each metabolite to an “unlimited” flux (99999 mmol/L/time). For single amino acid supplementation, one amino acid was supplemented one at a time. For multiple supplementations, all combinations of double, triple, quadruple… up to 7 amino acids were supplemented. (Computational time and resources dramatically increased after that and we are interested in supplement a minimum number of amino acids, rather than increasing all the amino acids.) If the protein synthesis flux increased compared to the normal condition, then it's assumed that the increase in availability of the amino acid increased protein synthesis flux.

**Figure 1:**
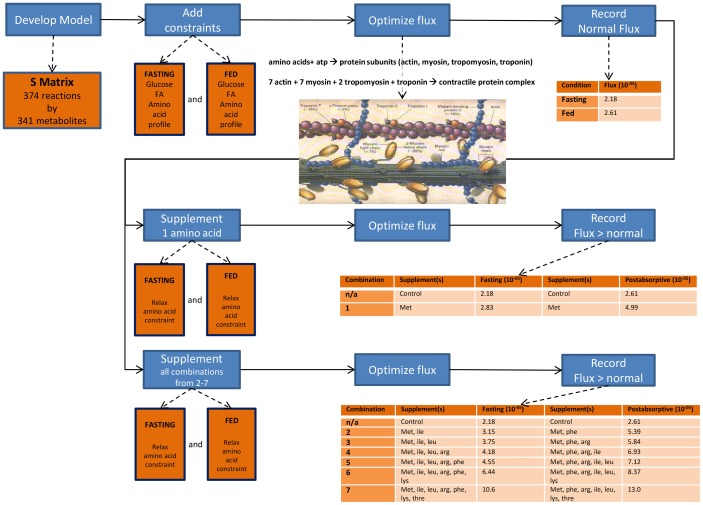
Experimental Procedure. This figure describes the work flow used for the experiment. A flux balance model of 374 reactions and 341 metabolites describing human muscle tissue was developed. Flux constraints based on fasting (Cynober 2002) and fed (Cynober et al 2002, Aoki et al 1976, Pozefsky et al 1969) levels of glucose, fatty acids, and amino acids in blood was added. With the given constraint, the contractile protein flux was determined. Supplementation was simulated by relaxing the constraint on amino acids singly and in all combinations from 2–7 amino acids. If the resultant flux was greater than the control flux, then the flux and conditions were recorded. (The contractile protein schematic is from Spirito [22].).

We first determined the protein synthesis flux for the contractile proteins in each of the four muscle tissues (Type 1, Type 2a, Type 2x, and Type 2b) under both fasting and post-absorptive states (Table S1). We then optimized protein synthesis (contractile protein complex) in both control and supplement conditions and compared the resulting fluxes after optimization. We simulate supplementation by removing the constraint on the supplemented amino acid(s). We then optimized protein synthesis through the contractile protein complex described. The method is outlined in Figure 1.

### Data Analysis

To make sense of the data and which amino acids are actually an artifact of being important or just available in lower quantities, the number of amino acids that are needed were compared to the number that were available (Table S2). The number of each amino acid that is needed is based on a count of the amino acids in each of the protein and subunits of the proteins which make up the protein complex unit (Table S1). The amount that is available was determined by converting the amount available in mmol divided by the control protein flux: 2.18×10^−5^ mmol/time. Next, the difference of what is needed from what is available was determined and the amino acids were ranked by difference (*Expected Supplementation Effect*). The *Actual Supplementation Effect* is which amino acids were effective at increasing protein flux from single to 7 amino acid combinations (Table S2).

## Results

### Single Amino Acid Supplementation

The purpose of this study is to determine if constraint based modeling is an effective tool for investigating nutrition supplementation. To achieve this, we ask a specific question: can increasing single or multiple amino acid uptake flux lead to increased muscle synthesis of the contractile protein complexes (see methods) assuming the muscle tissue is primed for protein synthesis (post-exercise stimulus) without including genetic and regulatory controls. We first investigate single acid supplementation, and after optimizing the model for contractile protein synthesis under fasting and post-absorptive conditions, we relaxed each amino acid constraint one at a time. For single amino acid supplementation, we found that for both states methionine was the only amino acid effective at increasing the protein flux above the control condition (Table 2). Quite often supplementation regimes occur in combinations with many supplements/amino acids.

**Table 2:** Type 2a contractile protein flux synthesis in fasting and post-absorptive states for every combination of 1–7 amino acids.

Combination	Supplement(s) – Fasting	Fasting (10^–05^) mmol/L/time*	Supplement(s) – post-absorptive	Post-absorptive (10^–05^) mmol/L/time*
n/a	Control	2.18	Control	2.61
1	Methionine	2.83	Methionine	4.99
2	Methionine, Isoleucine	3.15	Methionine, Phenylalanine	5.39
3	Methionine, Isoleucine, Leucine	3.75	Methionine, Phenylalanine, Arginine	5.84
4	Methionine, Isoleucine, Leucine, Arginine	4.18	Methionine, Phenylalanine, Arginine, Isoleucine	6.93
5	Methionine, Isoleucine, Leucine, Arginine, Phenylalanine	4.55	Methionine, Phenylalanine, Arginine, Isoleucine, Leucine	7.12
6	Methionine, Isoleucine, Leucine, Arginine, Phenylalanine, Lysine	6.44	Methionine, Phenylalanine, Arginine, Isoleucine, Leucine, Lysine	8.37
7	Methionine, Isoleucine, Leucine, Arginine, Phenylalanine, Lysine, Threonine	10.6	Methionine, Phenylalanine, Arginine, Isoleucine, Leucine, Lysine, Threonine	13.0

* time is not defined.

### Multiple Amino Acid Supplementation

Our next step was to determine if the model predicted that specific combinations of supplements result in increased protein synthesis. We then relaxed the constraints for every combination of 2–7 amino acids to determine what combinations would increase contractile protein synthesis above the control fasting and post-absorptive states. We found that once we supplemented with methionine the combination in the fasting and post-absorptive states vary slightly. For instance, for the combination of three amino acids in the fasting state differs than the optimal combination in the post-absorptive state (Table 2). In fasting, methionine, isoleucine, and leucine were most optimal at increasing protein synthesis, yet in the post-absorptive state, the optimal combination of amino acids is methionine, arginine, and phenylalanine.

However, when investigating the optimal condition for five supplements, the results in both the fasting and post-absorptive states were the same: methionine, arginine, isoleucine, leucine, and phenylalanine (Table 2). In six and seven combinations, the addition of lysine and threonine increased protein synthesis, respectively (Table 2).

## Discussion

The significance of these results is apparent when comparing the amount of amino acids needed to build one contractile protein complex to what is available (Table S2). One would expect that methionine and isoleucine, respectively, should be the top two amino acids needed to increase protein flux because these essential amino acids have the largest differences between what is available and what is needed (Table S2B). As expected, this is also what the model shows. However, it is interesting the model then predicts that leucine and valine are the next important essential amino acids necessary at increasing the protein flux even though, based on availability differences, one would expect phenylalanine and tryptophan would be the next effective supplements (Table S2B). Arginine, a conditionally essential amino acid is also greater than expected in supplementation affect based on essentiality and availability differences (Table S2B). We obtain similar results when investigating the results in post-absorptive state. According to the rank between what is available and what is needed, methionine, isoleucine, and leucine should be the most effective, but in fact the model shows that phenylalanine and arginine are more effective.

The advantage of using this metabolic modeling is being able to track the fluxes for each of the metabolites. Thus, we can predict why our results differ than what is expected if availability is the only factor. Not only is there an increase in contractile protein synthesis when relaxing the constraint on methionine, the model predicts that there is a change in how energy is produced in support for protein synthesis (File S1 and Figure S1E). As seen in Figure S1E, there is a decrease in glycolysis from glucose and a decrease in fatty acid oxidation from palmitate. Some of the energy for the synthesis process comes from increased lysine oxidation among changes in other amino acid catabolism, such as asparagine, serine, and glycine. Glutamine is predicted to be made in excess and transported out of the muscle tissue. This process increases the TCA cycle and the NADH shuttle to the mitochondria (facilitated by the malate and alpha-ketoglutarate exchange seen in Figure S1E).

Although relaxing the constraints on the other single amino acid supplementation conditions did not increase contractile protein synthesis, it did alter the metabolic profile of the network compared to the control condition (File S1). Here we will describe a few of the different profiles based on different supplementation conditions. For example, the model predicts the usage of the BCAA's as a preferred energy source, even in single amino acid supplementation (File S1 and Figures S1C and S1D). Thus, it is predicted that all the available BCAA's (isoleucine, leucine, and valine) will be absorbed and used, whether as a building block for proteins or an energy source for ATP production. Increasing the available BCAA's increases the flux through the branched chain amino acid transaminase enzyme (BCAT-2). Increased flux through BCAT-2 increases the byproduct of glutamate. The increased BCAT-2 flux requires an increase in the fluxes through the malate-aspartate shuttles and ultimately leads to an increase in serine production. This process reduces the need of serine, and as such, less serine is taken into the cell compared to no BCAA supplementation. In summary, the model shows that if the availability of BCAA's is increased, then the muscle tissue doesn't require as much serine to build contractile proteins (File S1 and Figures S1C and S1D). This is also a similar process for supplementation of alanine, aspartate, glutamate, and proline (File S1). Under these conditions less serine is required to achieve the same result in protein synthesis flux. Also, when extra glutamine is supplied to the model, less asparagine is needed (File S1). The model predicts that these amino acids are converted to 3 carbon metabolites and feed into the glycolysis pathways and ultimately increases the TCA cycle.

### Comparison to Literature –BCAA

The model's predictions are more similar to what is found in literature than what is expected based on availability differences. Branched-chain amino acids (BCAAs) have been found to greatly increase protein synthesis. Even though BCAA catabolism increases after exercise [21], a popular current hypothesis is that they have a signaling effect and increase enzymes necessary for protein synthesis [2], [21]. The model used here is void of any genetic controls, yet it still shows that increasing the bioavailability of these amino acids is enough to predict an increase in protein synthesis flux (Table S2). The model predicts that the BCAA's are preferentially oxidized over glucose and fatty acids and used as an energy source rather than as a building block for the contractile proteins (File S4, File S5, and Figure S1B). It is possible that in addition to a signaling effect, BCAA's are effective at increasing protein synthesis by adding a more efficient acute energy source when energy is needed quickly for protein synthesis.

### Comparison to Literature – Arginine

Another pertinent revelation from the model is an amino acid combination where arginine is required to increase protein synthesis (Table 2, row 4 Fasting, row 3 post-absorptive). In the literature, arginine supplementation had mixed results. It was hypothesized that arginine is only effective under certain amino acid profiles [3], but the exact profile is unknown. As summarized in Table 2, row 4 in the fasting condition, the model shows that arginine is effective when supplemented with methionine, isoleucine, and leucine in the fasting state and effective in the post-absorptive state when combined with methionine and phenylalanine (Table 2). Most of the studies with arginine have been compared to either all the essential amino acids or none. The model predicts two different scenarios where arginine is effective at increasing protein synthesis and could explain the mixed results seen in the literature. This could indicate that during fasting conditions, in between meals, or pre-bed different supplementation strategies could be important compared to supplementation with meals.

### Comparison to Literature – Glutamine

Lastly, contrary to nutrition/supplement companies but consistent with the literature [4], glutamine supplementation is not predicted by the model to have an effect on protein synthesis. It is the most abundant amino acid found in fasting blood at concentrations above what is needed for protein synthesis of the contractile complexes (Table S2). Glutamine is a byproduct of many reactions and easily synthesized in the body. The model also shows glutamine is not found in the top seven amino acids which show an increase in protein synthesis (Table 2). In fact, the model shows an abundance of glutamine is made and eliminated from muscle tissue in some conditions (Files S4 and S5).

## Conclusion

One challenge in the application of FBA to study metabolism of multicellular organisms is choosing an appropriate objective function for the organism or tissue [9]. However, muscle tissue as compared to other tissues has more defined states, which we can be modeled: contraction and recovery/growth. Recovery from certain types of training leads to muscle tissue hypertrophy which is achieved by increasing the contractile proteins and scaffolding proteins in the muscle tissue. The contractile proteins differ slightly based on the muscle type: 1, 2a, 2x, and 2b. However, the amino acid composition of these proteins is similar enough that according to the model, amino acid supplementation effects protein synthesis equally (data not shown). This means that, similar supplementation regimes could be appropriate no matter the type of training goal. However, determining if there are differences between recovery and growth, one can slightly alter the objective function. For Type 1 muscle tissue (slow twitch), one could look at an objective function that would model increasing the concentration of mitochondria, while for Type 2 muscle tissue (fast twitch), the contractile protein synthesis may be more appropriate. To elucidate the differences even more, an objective function which incorporates a combination of mitochondrial proliferation and contractile muscle protein synthesis can be used to differentiate Type 2a, Type 2x, and Type 2b muscle tissues. Furthermore, the model predicts supplementation from fasting normo-aminoacidemia and post-absorptive states. These profiles were obtained by averaging the profiles of multiple individuals. Perhaps, by knowing the specific amino acids levels of an athlete or patient, one can use this model to predict an individualized supplement regimen appropriate for which muscle tissue recovery is desired. In addition, we use plasma concentration levels of amino acids as a proxy for amino acid flux. Differing concentrations can lead to differing results. Ultimately, we are looking at which uptake fluxes need to be increased to achieve the desired results. While our results are concurrent with literature, we can conceive that this can be used for personalized supplementations strategy. If we knew the concentrations and/or amino acid uptake fluxes of individuals, we can determine the most effective supplementation strategy to achieve the individual's objective.

We also recognize that the model used here is less complex than previously published muscle models [9], [11]. However, our model was curated at the level of muscle cell metabolism and includes specific protein synthesis for contractile protein complex, while the other models do not. Yet, increasing the complexity by incorporating the specific protein synthesis into other published muscle models, can potentially allow us to investigate other nutrients and metabolites often used for supplementation. In addition, the model only investigates acute response to increasing the uptake flux and does not implement genetic controls, which can lead to increased muscle synthesis post exercise. However, these results are still pertinent because we make the assumption that the muscle tissue is primed for protein synthesis, and we ask under this state, increasing which amino acid uptake fluxes are necessary to take advantage of protein synthesis.

This paper documents a novel and potentially important application for FBA. When looking at different nutrition, supplementation, and amino acid profiles, we gain a perspective on how this can affect the entire network. Not only did we predict amino acid supplementation and combinations that increased protein synthesis already found in literature, we showed that there can be various supplementation regimes that produce the same results but have differing metabolic landscapes. This gives us another tool to study nutrition and supplementation more effectively for promoting health. The results we obtained in this study can be considered as a proof of concept that introduces the prospect of using systems biology to investigate how supplementation and nutrition can affect human muscle metabolism. Clearly, additional work must be conducted both experimentally and computationally to refine the model and the perturbation protocols. The recent community efforts to build human tissue metabolic models would be very of great value for future work in this area, as well as conducting more detailed animal and human studies to validate the models and the predictions they make.

## Supporting Information

Figure S1 **Flux differences compared to control show how increased amino acids could be used.** This figure shows the changes in fluxes from the control solution described in methods. (a) Manually assigned color key for pathways in the model. (b) Shows the differences in fluxes between the three amino acid supplementation and control results. (c) Shows the differences in fluxes between isoleucine supplementing and control. (d) Shows the differences in fluxes between leucine supplementing and control. (e) Shows the differences in fluxes between methionine supplementing and control.
(PDF)

File S1 **Single Amino Acid Supplementation Flux Results.**
(PDF)

File S2 **Muscle Cell Model.**
(PDF)

File S3 **SBML Muscle Cell Model.**
(XML)

File S4 **Results: fasting.**
(PDF)

File S5 **Results: Post-absorptive.**
(PDF)

Table S1 **Table of the contractile protein complexes in different types of muscle tissue used for the model.**
(PDF)

Table S2 **Amino Acids Needed Compared to the Amino Acids Available to produce one contractile protein complex in Type 2a.**
(PDF)
