# Supplementary material for: To Supplement or Not to Supplement: A Metabolic Network Framework for Human Nutritional Supplements
Source: PLoS One. 2013 Aug 5;8(8):e68751. doi: 10.1371/journal.pone.0068751 (PMC3740736; doi:10.1371/journal.pone.0068751)

**Pathways**	**Color**	R	G	B	Rank
Objecitve		0	0	0	1
Glycolysis		0	255	0	2
Fatty Acid		255	255	0	3
Amino Acid Catabolism		0	0	255	4
Fatty Alcohol		255	150	0	5
Pentose Phosphate Pathway		0	150	150	6
TCA		255	0	0	7
Electron Transport Chain		200	0	0	8
Amino Acid Biosynthesis		100	200	255	9
Muscle		0	100	200	10
Polymerizatin		0	100	200	11
Protein Synthesis		0	100	200	12
Translation		0	100	200	13
Glycogen Synthesis		0	150	100	14
TAG		200	200	0	15
Amino Acid Mit Transport		175	150	200	16
Mitchondrial Transport		100	150	100	17
Cellular Uptake		150	150	100	18
Cellular Export		150	150	100	19
Exchange		150	150	100	20
Nutrient		150	150	100	21
Shuttle Cytosolic		100	50	50	22
Shuttle Mitochondrial		100	50	50	23
Creatine		150	0	200	24
Vitamin Metabolism		150	0	200	25
Glutathione biosysnthesis		150	0	200	26
Glutathione Redox		150	0	200	27
Signaling/Hormone		150	100	50	28

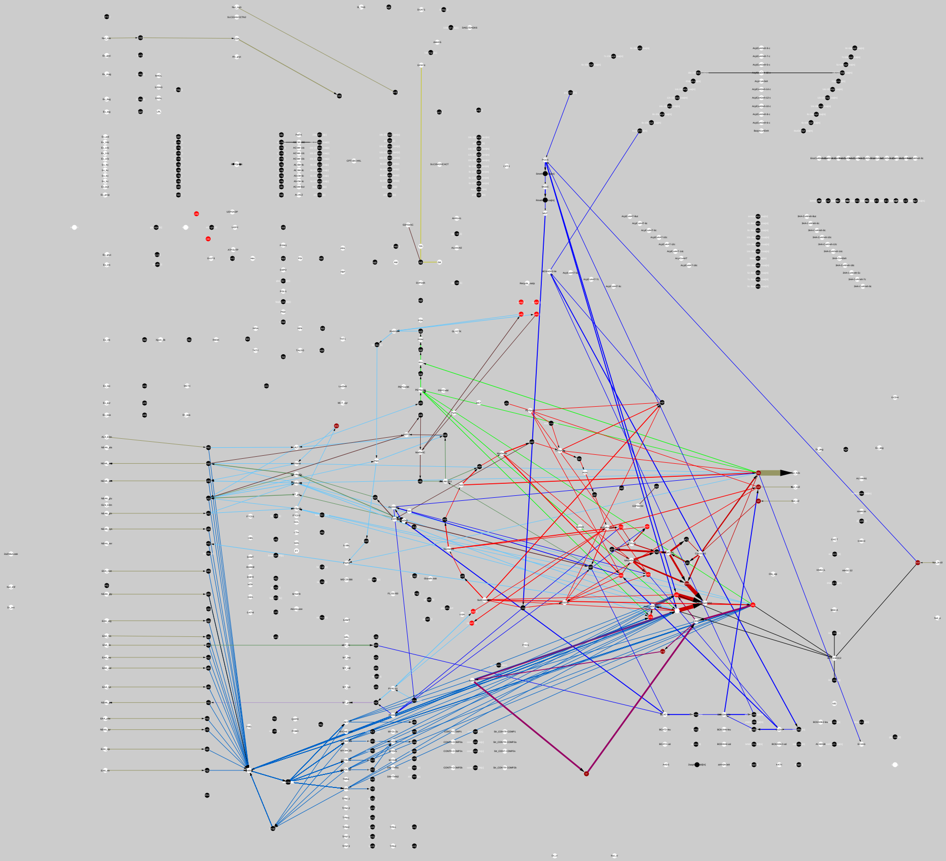

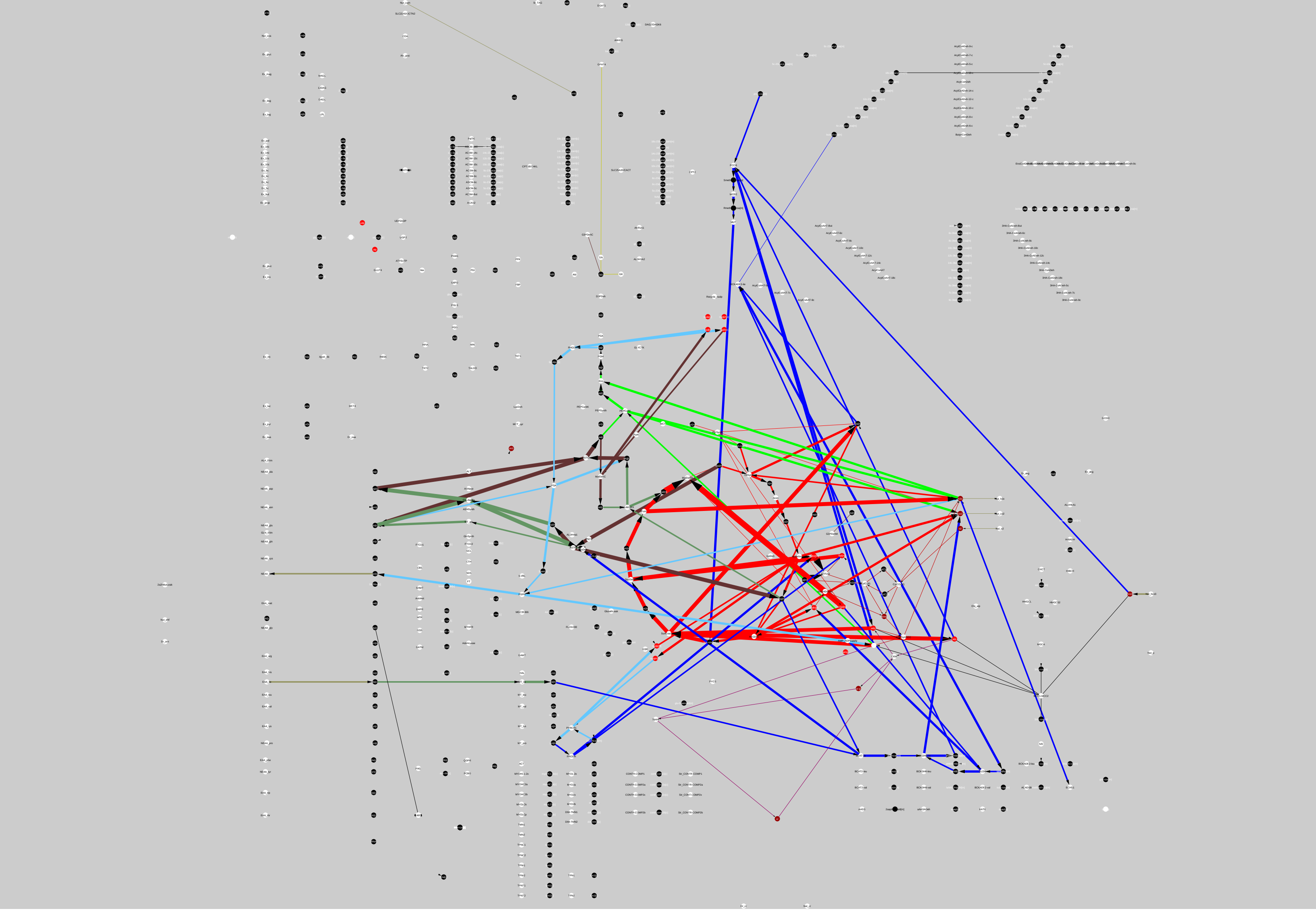

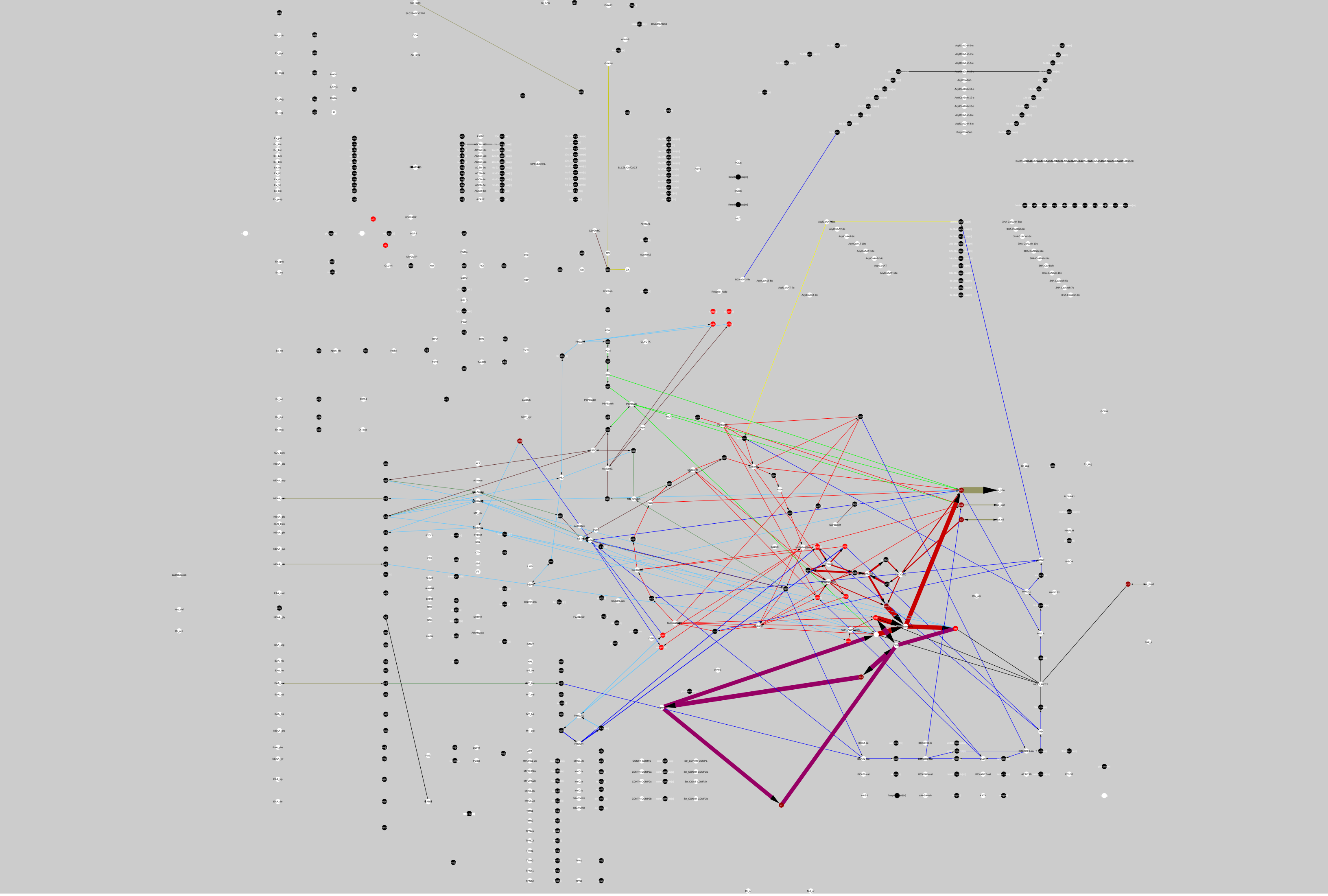

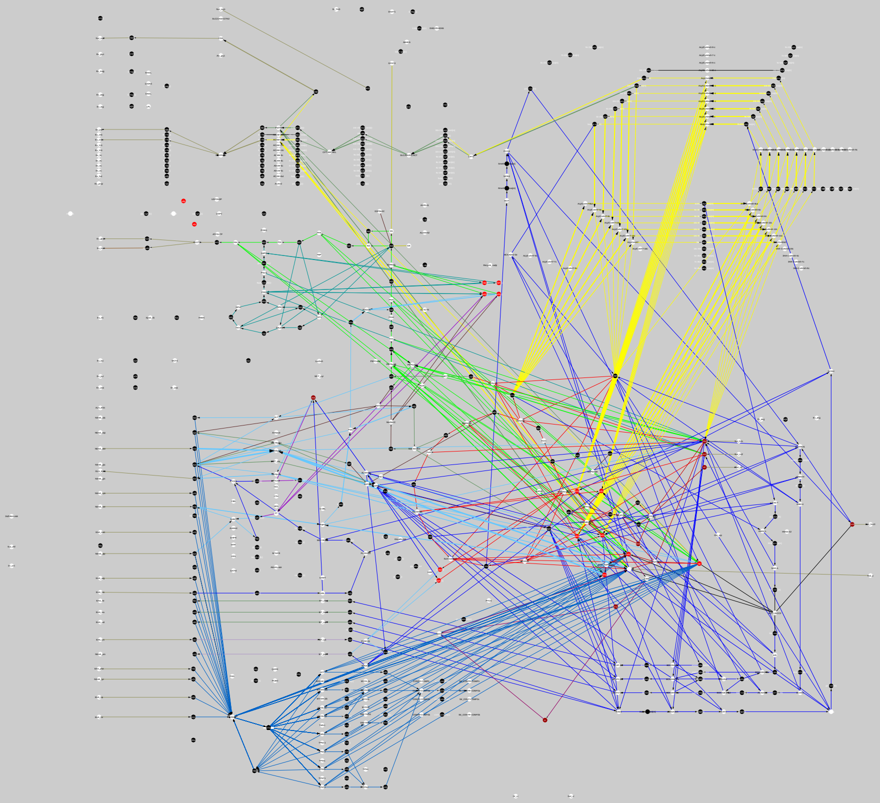

Supplement: Figure S1 — Flux differences compared to control show how increased amino acids could be used. This figure shows the changes in fluxes from the control solution described in methods. (a) Manually assigned color key for pathways in the model. (b) Shows the differences in fluxes between the three amino acid supplementation and control results. (c) Shows the differences in fluxes between isoleucine supplementing and control. (d) Shows the differences in fluxes between leucine supplementing and control. (e) Shows the differences in fluxes between methionine supplementing and control. (PDF) [file pone.0068751.s001.pdf]
