# Supplementary material for: To Supplement or Not to Supplement: A Metabolic Network Framework for Human Nutritional Supplements
Source: PLoS One. 2013 Aug 5;8(8):e68751. doi: 10.1371/journal.pone.0068751 (PMC3740736; doi:10.1371/journal.pone.0068751)
Supplement: File S1 — Single Amino Acid Supplementation Flux Results. (PDF) [file pone.0068751.s002.pdf]

	control	arg	his	ile	leu	lys	met	phe
	2.178E-05	2.178E-05	2.178E-05	2.178E-05	2.178E-05	2.178E-05	**2.83E-05**	2.178E-05
**arg**	-0.08	**-0.04645**	-0.04645	-0.04645	-0.04645	-0.04645	-0.060359	-0.04645
**his**	-0.082	-0.016006	**-0.016006**	-0.016006	-0.016006	-0.016006	-0.050845	-0.016006
**ile**	-0.062	-0.062	-0.062	**-40001.79**	-0.062	-0.062	-0.062	-0.062
**leu**	-0.123	-0.123	-0.123	-0.085126	**-0.300845**	-0.123	-0.123	-0.123
**lys**	-0.188	-0.089983	-0.178905	-0.089983	-0.089983	**-0.089983**	-0.162506	-0.089983
**met**	-0.025	-0.025	-0.025	-0.025	-0.025	-0.025	**-0.032486**	-0.025
**phe**	-0.057	-0.029682	-0.029682	-0.029682	-0.029682	-0.029682	-0.03857	**-0.029682**
**thr**	-0.14	-0.047343	-0.047343	-0.047343	-0.047343	-0.047343	-0.061519	-0.047343
**trp**	-0.044	-0.004943	-0.004943	-0.004943	-0.004943	-0.004943	-0.006424	-0.004943
**val**	-0.233	-0.233	-0.233	-0.233	-0.233	-0.233	-0.233	-0.233
**ala**	-0.333	-0.333	-0.333	-0.333	-0.333	-0.333	-0.333	-0.333
**asn**	-0.041	0.5371577	0.7150026	-0.041	0.7150026	0.5371577	-0.041	0.5371577
**asp**	-0.003	-0.003	-0.003	-0.003	-0.003	-0.003	-0.003	-0.003
**cys**	-0.052	-0.010279	-0.010279	-0.010279	-0.010279	-0.010279	-0.013356	-0.010279
**glu**	-0.024	-0.024	-0.024	-0.024	-0.024	-0.024	-0.024	-0.024
**gln**	-0.586	-0.586	-0.586	-0.586	-0.586	-0.586	-0.586	-0.586
**gly**	-0.23	-0.047147	-0.047147	-0.047147	-0.047147	-0.047147	-0.091311	-0.047147
**pro**	-0.168	-0.168	-0.168	-0.168	-0.168	-0.168	-0.168	-0.168
**ser**	-0.114	0.3848432	0.2069983	40002.148	0.2069983	0.3848432	0.1993168	0.3848432
**tyr**	-0.059	-0.017378	-0.017378	-0.017378	-0.017378	-0.017378	-0.022581	-0.017378

thr	trp	val	ala	asn	asp	cys	glu	gln
2.178E-05	2.178E-05	2.178E-05	2.178E-05	2.178E-05	2.178E-05	2.178E-05	2.178E-05	2.178E-05
-0.04645	-0.04645	-0.04645	-0.04645	-0.04645	-0.04645	-0.04645	-0.04645	-0.04645
-0.016006	-0.016006	-0.016006	-0.016006	-0.016006	-0.082	-0.016006	-0.016006	-0.016006
-0.062	-0.062	-0.062	-0.062	-0.062	-0.062	-0.062	-0.062	-0.062
-0.123	-0.123	-0.085126	-0.085126	-0.123	-0.123	-0.123	-0.085126	-0.085126
-0.089983	-0.089983	-0.089983	-0.089983	-0.178905	-0.188	-0.089983	-0.089983	-0.089983
-0.025	-0.025	-0.025	-0.025	-0.025	-0.025	-0.025	-0.025	-0.025
-0.029682	-0.029682	-0.029682	-0.057	-0.029682	-0.057	-0.029682	-0.057	-0.029682
**-0.047343**	-0.047343	-0.047343	-0.047343	-0.047343	-0.047343	-0.047343	-0.047343	-0.047343
-0.004943	**-0.004943**	-0.004943	-0.004943	-0.004943	-0.004943	-0.004943	-0.004943	-0.004943
-0.233	-0.233	**-52629.9**	-0.086286	-0.233	-0.042204	-0.233	-0.042204	-0.233
-0.333	-0.333	-0.333	**-333332.6**	-0.333	-0.333	-0.333	-0.333	-0.333
0.5371577	0.5371577	-0.034669	-0.034669	**0.7150026**	62497.926	0.5371577	-0.041	124974.56
-0.003	-0.003	-0.003	-0.003	-0.003	**-124994**	-0.003	124999.73	-0.003
-0.010279	-0.010279	-0.010279	-0.010279	-0.010279	-0.010279	**-0.010279**	-0.010279	-0.010279
-0.024	-0.024	-0.024	-0.024	-0.024	-0.024	-0.024	**-125000.5**	-0.024
-0.586	-0.586	-0.586	-0.586	-0.586	-0.586	-0.586	-0.586	**-124974.7**
-0.047147	-0.047147	-0.047147	-0.047147	-0.047147	-0.113141	-0.047147	-0.047147	-0.047147
-0.168	-0.168	-0.168	-0.168	-0.168	-0.168	-0.168	-0.028484	-0.168
0.3848432	0.3848432	52630.089	333332.45	0.2069983	-0.114	0.3848432	-0.114	0.4227169
-0.017378	-0.017378	-0.017378	0.0099399	-0.017378	0.0099399	-0.017378	0.0099399	-0.017378

**gly**	**pro**	**ser**	**tyr**
2.178E-05	2.178E-05	2.178E-05	2.178E-05
-0.04645	-0.04645	-0.04645	-0.04645
-0.016006	-0.016006	-0.016006	-0.016006
-0.062	-0.062	-0.062	-0.062
-0.123	-0.085126	-0.123	-0.123
-0.089983	-0.089983	-0.089983	-0.089983
-0.025	-0.025	-0.025	-0.025
-0.029682	-0.029682	-0.029682	-0.029682
-0.047343	-0.047343	-0.047343	-0.047343
-0.004943	-0.004943	-0.004943	-0.004943
-0.233	-0.042204	-0.233	-0.233
-0.333	-0.333	-0.333	-0.333
0.5371577	-0.034669	0.5371577	0.5371577
-0.003	-0.003	-0.003	-0.003
-0.010279	-0.010279	-0.010279	-0.010279
-0.024	199999.3	-0.024	-0.024
-0.586	-0.586	-0.586	-0.586
**-0.047147**	-0.047147	-0.047147	-0.047147
-0.168	**-199999.7**	-0.168	-0.168
0.3848432	-0.046124	**0.3848432**	0.3848432
-0.017378	-0.017378	-0.017378	**-0.017378**

Flux	
**Prot Synth**	
Obj_atp	atp --> adp + p
Ex_ins	insulin[e] <==>
Ex_gluc	gluc[e] <==>
Ex_lac	lac[e] <==>
Ex_pyr	pyr[e] <==>
Ex_pal	pal[e] <==>
Ex_prop	prop[e] <==>
Ex_but	but[e] <==>
Ex_5c	5c-fa[e] <==>
Ex_6c	6c-fa[e] <==>
Ex_8c	8c-fa[e] <==>
Ex_9c	9c-fa[e] <==>
Ex_10c	10c-fa[e] <==>
Ex_12c	12c-fa[e] <==>
Ex_14c	14c-fa[e] <==>
Ex_18c	18c-fa[e] <==>
Ex_glyc	glyc[e] <==>
Ex_tag	tag[e] <==>
Ex_dag	12dag[e] <==>
Ex_mag	1mag[e] <==>
Ex_akg	akg[e] -->
Ex_oaa	oaa[e] -->
Ex_rib	rib[e] <==>
Nut_o2	o2 <==>
Nut_co2	co2 <==>
Nut_h2o	h2o <==>
Nut_p	p <==>
Nut_hco3	hco3 <==>
Nut_coa	coa[e] <==>
Nut_carn	carn[e] <==>
Nut_dhf	dhf[c] <==>
LPL	tag[e] + h2o --> pal[e] + 12dag[e]
DAGL	12dag[e] + h2o --> 2mag[e] + pal[e]
12DAG	2mag[e] <==> 1mag[e]
MAGL	1mag[e] + h2o --> glyc[e] + pal[e]
GLUT4	insulin[e] + gluc[e] --> gluc[c]
MCT4	lac[e] <==> lac[c]
MCT_pyr	pyr[e] --> pyr[c]
FAT/CD36	pal[e] --> pal[c]
FATprop	prop[e] <==> prop[c]
FATbut	but[e] <==> but[c]
FAT5c	5c-fa[e] <==> 5c-fa[c]
FAT6c	6c-fa[e] <==> 6c-fa[c]
FAT8c	8c-fa[e] <==> 8c-fa[c]
FAT9c	9c-fa[e] <==> 9c-fa[c]

FAT10c	10c-fa[e] <==> 10c-fa[c]
FAT12c	12c-fa[e] <==> 12c-fa[c]
FAT14c	14c-fa[e] <==> 14c-fa[c]
FAT18c	18c-fa[e] <==> 18c-fa[c]
Alc_glyc	glyc[e] <==> glyc[c]
COA	coa[e] --> coa[c]
SLC22A5/OCTN2	carn[e] <==> carn[c]
Xport_rib	rib[e] --> rib[c]
Dr_akg	akg[c] --> akg[e]
Dr_oaa	oaa[c] --> oaa[e]
Hex	atp + gluc[c] --> adp + p + g6p[c]
PGI	g6p[c] <==> f6p[c]
PFK	atp + f6p[c] --> adp + fbp[c]
FBP	fbp[c] + h2o --> f6p[c] + p
Ald	fbp[c] <==> dhap[c] + g3p[c]
TPI	g3p[c] <==> dhap[c]
D3PDeh	g3p[c] + nad[c] <==> dpg[c] + nadh[c]
PGK	dpg[c] + adp <==> 3pg[c] + atp
PGM	3pg[c] <==> 2pg[c]
Eno	2pg[c] <==> pep[c] + h2o
PyrK	pep[c] + adp --> pyr[c] + atp
PEPSynth	pyr[c] + atp + h2o --> pep[c] + amp + p
PEPCarbK	oaa[c] + atp --> co2 + pep[c] + adp
PEPCarbl	oaa[c] + p --> pep[c] + h2o + co2
LacDeh	lac[c] + nad[c] <==> pyr[c] + nadh[c]
MalDehC(mdh1)	oaa[c] + nadh[c] <==> mal[c] + nad[c]
ASTC	asp[c] + akg[c] <==> glu[c] + oaa[c]
G3PDehC	dhap[c] + nadh[c] <==> glyc3p[c] + nad[c]
Mal-AKGEX	mal[c] + akg[m] <==> mal[m] + akg[c]
Glu-AspEx	asp[m] + glu[c] <==> asp[c] + glu[m]
MCT	pyr[c] --> pyr[m]
ASTM	glu[m] + oaa[m] <==> asp[m] + akg[m]
G3PDehM	glyc3p[m] + fad[m] <==> dhap[m] + fadh2[m]
PyrSynth/PDH	pyr[m] + coa[m] + nad[m] --> acetyl-coa[m] + nadh[m] + co2
CitSynth	oaa[m] + acetyl-coa[m] + h2o <==> cit[m] + coa[m]
Acon	cit[m] <==> iso[m]
IsoDeh	iso[m] + nad[m] --> akg[m] + co2 + nadh[m]
IsoDehnadph	iso[m] + nadp[m] --> akg[m] + co2 + nadph[m]
AKGDeh	akg[m] + coa[m] + nad[m] --> suc-coa[m] + co2 + nadh[m]
SunCoaSynth	suc-coa[m] + adp + p <==> suc[m] + coa[m] + atp
SucDeh(I)	suc[m] + fad[m] <==> fum[m] + fadh2[m]
Fum	fum[m] <==> mal[m] + h2o
MalDehM	mal[m] + nad[m] <==> oaa[m] + nadh[m]
NADHDeh(II)	nadh[m] + uq[m] --> nad[m] + uqh2[m] + 4 h[out]
ETFUO	fadh2[m] + uq[m] --> fad[m] + uqh2[m]
UCytC(III)	2 ocytC[m] + uqh2[m] --> 2 rcytC[m] + uq[m] + 4 h[out]
CytCO(IV)	o2 + 4 rcytC[m] --> 2 h2o + 4 ocytC[m] + 8 h[out]

ATPSynth(V)	adp + p + 4 h[out] <==> atp + h2o
AMP_ADPsupply	amp + p <==> adp
PalTK	pal[c] + coa[c] + atp --> 234pal-coa[c] + 2 p + amp
CPT1B/CHKL	carn[c] + 234pal-coa[c] <==> pal-carn[c] + coa[c]
SLC25A20/CACT	pal-carn[c] + carn[m] --> carn[c] + pal-carn[m]
CPT2	pal-carn[m] + coa[m] --> 234pal-coa[m] + carn[m]
CRAT(mit)	acetyl-carn[m] + coa[m] <==> carn[m] + acetyl-coa[m]
CRAT Transport	acetyl-carn[c] + carn[m] <==> carn[c] + acetyl-carn[m]
CRAT(cyt)	carn[c] + acetyl-coa[c] <==> acetyl-carn[c] + coa[c]
Acetyl-coaLeak	acetyl-coa[c] -->
AcylCoADeh	234pal-coa[m] + fad[m] --> fadh2[m] + 23pal-coa[m]
EnoCoADeh	23pal-coa[m] + h2o <==> 3ohpal-coa[m]
3HA-CoADeh	3ohpal-coa[m] + nad[m] <==> 3ketopal-coa[m] + nadh[m]
AcylCoAAT	3ketopal-coa[m] + coa[m] --> 14c-234acyl-coa[m] + acetyl-coa[m]
AcylCoADeh-18-c	18c-234acyl-coa[m] + fad[m] --> 18c-t2enoyl-coa[m] + fadh2[m]
EnoCoADeh-18c	18c-t2enoyl-coa[m] + h2o <==> 18c-3ohacyl-coa[m]
3HA-CoADeh-18c	18c-3ohacyl-coa[m] + nad[m] <==> 18c-3ketoacyl-coa[m] + nadh[m]
AcylCoAAT-18c	18c-3ketoacyl-coa[m] + coa[m] --> 234pal-coa[m] + acetyl-coa[m]
AcylCoADeh-14-c	14c-234acyl-coa[m] + fad[m] --> 14c-t2enoyl-coa[m] + fadh2[m]
EnoCoADeh-14c	14c-t2enoyl-coa[m] + h2o <==> 14c-3ohacyl-coa[m]
3HA-CoADeh-14c	14c-3ohacyl-coa[m] + nad[m] <==> 14c-3ketoacyl-coa[m] + nadh[m]
AcylCoAAT-14c	14c-3ketoacyl-coa[m] + coa[m] --> 12c-234acyl-coa[m] + acetyl-coa[m]
AcylCoADeh-12-c	12c-234acyl-coa[m] + fad[m] --> 12c-t2enoyl-coa[m] + fadh2[m]
EnoCoADeh-12c	12c-t2enoyl-coa[m] + h2o <==> 12c-3ohacyl-coa[m]
3HA-CoADeh-12c	12c-3ohacyl-coa[m] + nad[m] <==> 12c-3ketoacyl-coa[m] + nadh[m]
AcylCoAAT-12c	12c-3ketoacyl-coa[m] + coa[m] --> 10c-234acyl-coa[m] + acetyl-coa[m]
AcylCoADeh-10-c	10c-234acyl-coa[m] + fad[m] --> 10c-t2enoyl-coa[m] + fadh2[m]
EnoCoADeh-10c	10c-t2enoyl-coa[m] + h2o <==> 10c-3ohacyl-coa[m]
3HA-CoADeh-10c	10c-3ohacyl-coa[m] + nad[m] <==> 10c-3ketoacyl-coa[m] + nadh[m]
AcylCoAAT-10c	10c-3ketoacyl-coa[m] + coa[m] --> 8c-234acyl-coa[m] + acetyl-coa[m]
AcylCoADeh-8-c	8c-234acyl-coa[m] + fad[m] --> 8c-t2enoyl-coa[m] + fadh2[m]
EnoCoADeh-8c	8c-t2enoyl-coa[m] + h2o <==> 8c-3ohacyl-coa[m]
3HA-CoADeh-8c	8c-3ohacyl-coa[m] + nad[m] <==> 8c-3ketoacyl-coa[m] + nadh[m]
AcylCoAAT-8c	8c-3ketoacyl-coa[m] + coa[m] --> 6c-234acyl-coa[m] + acetyl-coa[m]
AcylCoADeh-6-c	6c-234acyl-coa[m] + fad[m] --> 6c-t2enoyl-coa[m] + fadh2[m]
EnoCoADeh-6c	6c-t2enoyl-coa[m] + h2o <==> 6c-3ohacyl-coa[m]
3HA-CoADeh-6c	6c-3ohacyl-coa[m] + nad[m] <==> 6c-3ketoacyl-coa[m] + nadh[m]
AcylCoAAT-6c	6c-3ketoacyl-coa[m] + coa[m] --> butyryl-coa[m] + acetyl-coa[m]
ButyrlCoADeh	butyryl-coa[m] + fad[m] --> tbut2enoyl-coa[m] + fadh2[m]
EnoCoADeh-But	tbut2enoyl-coa[m] + h2o <==> 3ohbutyryl-coa[m]
3HA-CoADeh-But	3ohbutyryl-coa[m] + coa[m] <==> acetoacetyl-coa[m]
AcylCoAAT-But	acetoacetyl-coa[m] --> 2 acetyl-coa[m]
GK	glyc[c] + atp <==> glyc3p[c] + adp
GPAT4	glyc3p[c] + 234pal-coa[c] <==> 1acyl-g3p[c] + coa[c]
ABHD5	1acyl-g3p[c] + 234pal-coa[c] <==> 12dacyl-g3p[c] + coa[c]
DAGZ/DAGK6	12dacyl-g3p[c] + adp <==> 12dag[c] + atp
DGAT1	12dag[c] + 234pal-coa[c] <==> tag[c] + coa[c]

St_TAG	tag[c] -->
ACAS2	prop[c] + atp + coa[c] --> prop-coa[c] + adp + p
ACSM-But	but[c] + atp + coa[c] --> butyryl-coa[c] + adp + p
ASCM-5c	5c-fa[c] + atp + coa[c] --> 5c-234acyl-coa[c] + adp + p
ASCM-6c	6c-fa[c] + atp + coa[c] --> 6c-234acyl-coa[c] + adp + p
ACSM-8c	8c-fa[c] + atp + coa[c] --> 8c-234acyl-coa[c] + adp + p
ACSM-9c	9c-fa[c] + atp + coa[c] --> 9c-234acyl-coa[c] + adp + p
ACSM-10c	10c-fa[c] + atp + coa[c] --> 10c-234acyl-coa[c] + adp + p
ACSM-12c	12c-fa[c] + atp + coa[c] --> 12c-234acyl-coa[c] + adp + p
ACSM-14c	14c-fa[c] + atp + coa[c] --> 14c-234acyl-coa[c] + adp + p
ACSM-18c	18c-fa[c] + atp + coa[c] --> 18c-234acyl-coa[c] + adp + p
CPT1B/CHKL	prop-coa[c] + carn[c] --> prop-carn[c] + coa[c]
CPT1B/CHKL	butyryl-coa[c] + carn[c] --> butyryl-carn[c] + coa[c]
CPT1B/CHKL	5c-234acyl-coa[c] + carn[c] --> 5c-234acyl-carn[c] + coa[c]
CPT1B/CHKL	6c-234acyl-coa[c] + carn[c] --> 6c-234acyl-carn[c] + coa[c]
CPT1B/CHKL	8c-234acyl-coa[c] + carn[c] --> 8c-234acyl-carn[c] + coa[c]
CPT1B/CHKL	9c-234acyl-coa[c] + carn[c] --> 9c-234acyl-carn[c] + coa[c]
CPT1B/CHKL	10c-234acyl-coa[c] + carn[c] --> 10c-234acyl-carn[c] + coa[c]
CPT1B/CHKL	12c-234acyl-coa[c] + carn[c] --> 12c-234acyl-carn[c] + coa[c]
CPT1B/CHKL	14c-234acyl-coa[c] + carn[c] --> 14c-234acyl-carn[c] + coa[c
CPT1B/CHKL	18c-234acyl-coa[c] + carn[c] --> 18c-234acyl-carn[c] + coa[c
SLC25A20/CACT	prop-carn[c] + carn[m] --> prop-carn[m] + carn[c]
SLC25A20/CACT	butyryl-carn[c] + carn[m] --> butyryl-carn[m] + carn[c]
SLC25A20/CACT	5c-234acyl-carn[c] + carn[m] --> 5c-234acyl-carn[m] + carn[c]
SLC25A20/CACT	6c-234acyl-carn[c] + carn[m] --> 6c-234acyl-carn[m] + carn[c]
SLC25A20/CACT	8c-234acyl-carn[c] + carn[m] --> 8c-234acyl-carn[m] + carn[c]
SLC25A20/CACT	9c-234acyl-carn[c] + carn[m] --> 9c-234acyl-carn[m] + carn[c]
SLC25A20/CACT	10c-234acyl-carn[c] + carn[m] --> 10c-234acyl-carn[m] + carn[c]
SLC25A20/CACT	12c-234acyl-carn[c] + carn[m] --> 12c-234acyl-carn[m] + carn[c]
SLC25A20/CACT	14c-234acyl-carn[c] + carn[m] --> 14c-234acyl-carn[m] + carn[c]
SLC25A20/CACT	18c-234acyl-carn[c] + carn[m] --> 18c-234acyl-carn[m] + carn[c]
CPT2	prop-carn[m] + coa[m] --> prop-coa[m] + carn[m]
CPT2	butyryl-carn[m] + coa[m] --> butyryl-coa[m] + carn[m]
CPT2	5c-234acyl-carn[m] + coa[m] --> 5c-234acyl-coa[m] + carn[m]
CPT2	6c-234acyl-carn[m] + coa[m] --> 6c-234acyl-coa[m] + carn[m]
CPT2	8c-234acyl-carn[m] + coa[m] --> 8c-234acyl-coa[m] + carn[m]
CPT2	9c-234acyl-carn[m] + coa[m] --> 9c-234acyl-coa[m] + carn[m]
CPT2	10c-234acyl-carn[m] + coa[m] --> 10c-234acyl-coa[m] + carn[m]
CPT2	12c-234acyl-carn[m] + coa[m] --> 12c-234acyl-coa[m] + carn[m]
CPT2	14c-234acyl-carn[m] + coa[m] --> 14c-234acyl-coa[m] + carn[m]
CPT2	18c-234acyl-carn[m] + coa[m] --> 18c-234acyl-coa[m] + carn[m]
AcylCoADeh-9-c	9c-234acyl-coa[m] + fad[m] --> 9c-t2enoyl-coa[m] + fadh2[m]
EnoCoADeh-9c	9c-t2enoyl-coa[m] + h2o <==> 9c-3ohacyl-coa[m]
3HA-CoADeh-9c	9c-3ohacyl-coa[m] + nad[m] <==> 9c-3ketoacyl-coa[m] + nadh[m]
AcylCoAAT-9c	9c-3ketoacyl-coa[m] + coa[m] --> 7c-234acyl-coa[m] + acetyl-coa[m]
AcylCoADeh-7-c	7c-234acyl-coa[m] + fad[m] --> 7c-t2enoyl-coa[m] + fadh2[m]
EnoCoADeh-7c	7c-t2enoyl-coa[m] + h2o <==> 7c-3ohacyl-coa[m]

3HA-CoADeh-7c	7c-3ohacyl-coa[m] + nad[m] <==> 7c-3ketoacyl-coa[m] + nadh[m]
AcylCoAAT-7c	7c-3ketoacyl-coa[m] + coa[m] --> 5c-234acyl-coa[m] + acetyl-coa[m]
AcylCoADeh-5-c	5c-234acyl-coa[m] + fad[m] --> 5c-t2enoyl-coa[m] + fadh2[m]
EnoCoADeh-5c	5c-t2enoyl-coa[m] + h2o <==> 5c-3ohacyl-coa[m]
3HA-CoADeh-5c	5c-3ohacyl-coa[m] + nad[m] <==> 5c-3ketoacyl-coa[m] + nadh[m]
AcylCoAAT-5c	5c-3ketoacyl-coa[m] + coa[m] --> acetyl-coa[m] + prop-coa[m]
AKR1A1	glyc[c] + nadp[c] --> glyc-ald[c] + nadph[c]
ALDH3A2	glyc-ald[c] + nad[c] + h2o --> glyc-ate[c] + nadh[c]
GLYCTK	glyc-ate[c] + atp --> 3pg[c] + adp + p
EAA_arg	arg[c] <==>
EAA_his	his[c] <==>
EAA_ile	ile[c] <==>
EAA_leu	leu[c] <==>
EAA_lys	lys[c] <==>
EAA_met	met[c] <==>
EAA_phe	phe[c] <==>
EAA_thr	thr[c] <==>
EAA_trp	trp[c] <==>
EAA_val	val[c] <==>
NEAA_ala	ala[c] <==>
NEAA_asn	asn[c] <==>
NEAA_asp	asp[c] <==>
NEAA_cys	cys[c] <==>
NEAA_glu	glu[c] <==>
NEAA_gln	gln[c] <==>
NEAA_gly	gly[c] <==>
NEAA_pro	pro[c] <==>
NEAA_ser	ser[c] <==>
NEAA_tyr	tyr[c] <==>
DLDH	laN6dhpl[m] + nad[m] <==> laN6ll[m] + nadh[m]
MT_val	val[c] --> val[m]
BCAT2-val	val[m] + akg[m] --> akisov[m] + glu[m]
BCKDHB-val	akisov[m] + laN6ll[m] --> laN6S2mpdhl[m] + co2
BCKADE2-val	laN6S2mpdhl[m] + coa[m] --> laN6dhpl[m] + isob-coa[m]
ACADSB	isob-coa[m] + fad[m] --> methacrl-coa[m] + fadh2[m]
ECHS1	methacrl-coa[m] + h2o --> 3hisob-coa[m]
HIBCH	3hisob-coa[m] + h2o --> 3hisob[m] + coa[m]
HIBADH	3hisob[m] + nad[m] --> methmalsemald[m] + nadh[m]
ALDH6A1	methmalsemald[m] + nad[m] + coa[m] + h2o --> prop-coa[m] + hco3 + nadh[m]
PCCB	prop-coa[m] + atp + hco3 --> Smethmal-coa[m] + adp + p
MCEE	Smethmal-coa[m] --> Rmethmal-coa[m]
MUT	Rmethmal-coa[m] --> suc-coa[m]
MT_ile	ile[c] --> ile[m]
BCAT-ile	ile[m] + akg[m] --> ak3mvalt[m] + glu[m]
BCKDHB-ile	ak3mvalt[m] + laN6ll[m] --> eN6S2mbdhpl[m] + co2
BCKADE2-ile	eN6S2mbdhpl[m] + coa[m] --> laN6dhpl[m] + t2metbut2en-coa[m]
ECHS1	t2metbut2en-coa[m] + h2o --> 3oh2metbut-coa[m]

HSD17B10	3oh2metbut-coa[m] + nad[m] --> 2met-acetoacetyl-coa[m] + nadh[m]
ACAT1	2met-acetoacetyl-coa[m] + coa[m] --> prop-coa[m] + acetyl-coa[m]
MT_leu	leu[c] --> leu[m]
BCAT2-leu	leu[m] + akg[m] --> akiso[m] + glu[m]
BCKDHB-leu	akiso[m] + laN6ll[m] --> eN6S3mbdhpl[m] + co2
BCKADE2-leu	eN6S3mbdhpl[m] + coa[m] --> laN6dhpl[m] + isov-coa[m]
IVD	isov-coa[m] + fad[m] --> 3mc-coa[m] + fadh2[m]
MCC1,MCC2	3mc-coa[m] + atp + hco3 --> 3mg-coa[m] + adp + p
MGCA	3mg-coa[m] + h2o --> 3h3mg-coa[m]
HMGCL	3h3mg-coa[m] --> acetacet[m] + acetyl-coa[m]
OXCT	acetacet[m] + suc-coa[m] --> suc[m] + acetoacetyl-coa[m]
HMGCS2	acetoacetyl-coa[m] + acetyl-coa[m] + h2o --> 3h3mg-coa[m] + coa[m]
GluDeh	glu[m] + nadp[m] + h2o <==> akg[m] + nadph[m] + nh3
GlnSynth	nh3 + glu[c] + atp --> gln[c] + adp + p
GLN_Elim	gln[c] -->
ALT	glu[c] + pyr[c] <==> akg[c] + ala[c]
ALA_Elim	ala[c] -->
SDS	ser[c] --> pyr[c] + nh3
CBS	ser[c] + hcys[c] --> cyst[c] + h2o
CTH	cyst[c] + h2o --> nh3 + cys[c] + 2ob[c]
ILVBL	pyr[c] + 2ob[c] --> 2a2hbut[c] + co2
2a2hButLeak	2a2hbut[c] -->
MT_lys	lys[c] --> lys[m]
AASS	lys[m] + akg[m] + nadh[m] <==> sacpn[m] + nad[m] + h2o
AASS	sacpn[m] + nad[m] + h2o <==> 2aap6semald[m] + glu[m] + nadh[m]
aAASADeh	2aap6semald[m] + nad[m] + h2o <==> aaap[m] + nadh[m]
KATII	aaap[m] + akg[m] <==> glu[m] + akap[m]
lysdeg	akap[m] + nad[m] + coa[m] <==> glut-coa[m] + nadh[m] + co2
GCDH	glut-coa[m] + fad[m] <==> tbut2enoyl-coa[m] + fadh2[m]
MT_pro	pro[c] --> pro[m]
PRODH	pro[m] + fad[m] --> 1pyrr5carb[m] + fadh2[m]
ALDH4a1	1pyrr5carb[m] + nad[m] + 2 h2o --> glu[m] + nadh[m]
GATM	gly[c] + arg[c] --> guan[c] + orn[m]
Dr_orn	orn[m] -->
GAMT	guan[c] + sal-met[c] --> cr + sal-hcys[c]
Dr_cr	cr -->
AdoHcyase	sal-hcys[c] + h2o --> hcys[c] + amp
MTR	hcys[c] + 5meth-thf[c] <==> met[c] + thf[c]
AdoMet	atp + met[c] + h2o --> sal-met[c] + 2 p
SHMT	ser[c] + thf[c] <==> gly[c] + 5methyln-thf[c]
MTHFR	5methyln-thf[c] + nadph[c] --> 5meth-thf[c] + nadp[c]
DHFR	dhf[c] + nadph[c] --> thf[c] + nadp[c]
HAL	his[c] --> uro[c] + nh3
FLJ31300	uro[c] + h2o --> 4im5prop[c]
MGC35366	4im5prop[c] + h2o --> form-glu[c]
FTCD1	form-glu[c] + thf[c] --> glu[c] + form-thf[c]
FTCD2	form-thf[c] <==> 5methn-thf[c] + nh3

FT	5methn-thf[c] + nadh[c] <==> 5methyln-thf[c] + nad[c]
ASNase	asn[c] + h2o --> asp[c] + nh3
ASNSynth	gln[c] + asp[c] + atp + h2o --> glu[c] + asn[c] + amp + 2 p
PHGDH	3pg[c] + nad[c] --> 3phdpyr[c] + nadh[c]
PSA	3phdpyr[c] + glu[c] --> 3pser[c] + akg[c]
PSPH	3pser[c] + h2o --> ser[c] + p
P5CS	glu[m] + atp --> glu-5-p[m] + adp + p
PYCS	glu-5-p[m] + nadph[m] --> glu-5-semald[m] + nadp[m]
Spont	glu-5-semald[m] --> 1pyrr5carb[m] + h2o
PYRCR1	1pyrr5carb[m] + nadph[m] --> pro[m] + nadp[m]
PAG	phe[c] + thb[c] --> tyr[c] + 4ah-thb[c]
PCBD	4ah-thb[c] --> dhb[c] + h2o
QDPR	dhb[c] + nadh[c] --> thb[c] + nad[c]
MT_glu	glu[m] <==> glu[c]
tRNA	trna[c] <==>
YARS	trna[c] + tyr[c] + atp <==> trna-tyr[c] + amp + 2 p
WARS	trna[c] + trp[c] + atp <==> trna-trp[c] + amp + 2 p
TARS	trna[c] + thr[c] + atp <==> trna-thr[c] + amp + 2 p
LARS	trna[c] + leu[c] + atp <==> trna-leu[c] + amp + 2 p
IARS	trna[c] + ile[c] + atp <==> trna-ile[c] + amp + 2 p
KARS	trna[c] + lys[c] + atp <==> trna-lys[c] + amp + 2 p
AARS	trna[c] + ala[c] + atp <==> trna-ala[c] + amp + 2 p
VARS	trna[c] + val[c] + atp <==> trna-val[c] + amp + 2 p
MARS	trna[c] + met[c] + atp <==> trna-met[c] + amp + 2 p
SARS	trna[c] + ser[c] + atp <==> trna-ser[c] + amp + 2 p
DARS	trna[c] + asp[c] + atp <==> trna-asp[c] + amp + 2 p
GARS	trna[c] + gly[c] + atp <==> trna-gly[c] + amp + 2 p
PARS	trna[c] + pro[c] + atp <==> trna-pro[c] + amp + 2 p
CARS	trna[c] + cys[c] + atp <==> trna-cys[c] + amp + 2 p
EPRS	trna[c] + glu[c] + atp <==> trna-glu[c] + amp + 2 p
QARS	trna[c] + gln[c] + atp <==> trna-gln[c] + amp + 2 p
RARS	trna[c] + arg[c] + atp <==> trna-arg[c] + amp + 2 p
FARS	trna[c] + phe[c] + atp <==> trna-phe[c] + amp + 2 p
HARS	trna[c] + his[c] + atp <==> trna-his[c] + amp + 2 p
NARS	trna[c] + asn[c] + atp <==> trna-asn[c] + amp + 2 p
ACT	29 trna-ala[c] + 6 trna-cys[c] + 22 trna-asp[c] + 28 trna-glu[c] + 12 trna-phe[c] + 28
MYOHC1-2x	171 trna-ala[c] + 17 trna-cys[c] + 98 trna-asp[c] + 261 trna-glu[c] + 56 trna-phe[c] +
MYOHC2a	172 trna-ala[c] + 18 trna-cys[c] + 93 trna-asp[c] + 265 trna-glu[c] + 58 trna-phe[c] +
MYOHC2b	168 trna-ala[c] + 17 trna-cys[c] + 94 trna-asp[c] + 263 trna-glu[c] + 61 trna-phe[c] +
MYOLCk	56 trna-ala[c] + 10 trna-cys[c] + 30 trna-asp[c] + 48 trna-glu[c] + 20 trna-phe[c] + 4
MYOLCp	12 trna-ala[c] + 2 trna-cys[c] + 15 trna-asp[c] + 15 trna-glu[c] + 12 trna-phe[c] + 13
MYO1-2x	2 myoHC1-2x[c] + 2 myoLCk[c] + 2 myoLCp[c] --> myo1[c]
MYO2a	2 myoHC2a[c] + 2 myoLCk[c] + 2 myoLCp[c] --> myo2a[c]
MYO2x	2 myoHC1-2x[c] + 2 myoLCk[c] + 2 myoLCp[c] --> myo2x[c]
MYO2b	2 myoHC2b[c] + 2 myoLCk[c] + 2 myoLCp[c] --> myo2b[c]
TMN1	38 trna-ala[c] + 1 trna-cys[c] + 19 trna-asp[c] + 61 trna-glu[c] + 1 trna-phe[c] + 3 tr
TMN2	28 trna-ala[c] + 3 trna-cys[c] + 14 trna-asp[c] + 54 trna-glu[c] + 1 trna-phe[c] + 4 tr

DIM-TMN1	2 tmn1[c] --> dim-tmn1[c]
DIM-TMN2	2 tmn2[c] --> dim-tmn2[c]
TPNC1	7 trna-ala[c] + 2 trna-cys[c] + 23 trna-asp[c] + 23 trna-glu[c] + 9 trna-phe[c] + 12 trr
TPNC2	12 trna-ala[c] + 1 trna-cys[c] + 19 trna-asp[c] + 26 trna-glu[c] + 10 trna-phe[c] + 13
TPNI1	15 trna-ala[c] + 3 trna-cys[c] + 10 trna-asp[c] + 22 trna-glu[c] + 2 trna-phe[c] + 6 trr
TPNI2	13 trna-ala[c] + 3 trna-cys[c] + 11 trna-asp[c] + 25 trna-glu[c] + 3 trna-phe[c] + 7 trr
TPNT1	20 trna-ala[c] + 1 trna-cys[c] + 12 trna-asp[c] + 54 trna-glu[c] + 6 trna-phe[c] + 12 t
TPNT2	24 trna-ala[c] + 18 trna-asp[c] + 49 trna-glu[c] + 4 trna-phe[c] + 9 trna-gly[c] + 4 trn
TPN1	tpnc1[c] + tpni1[c] + tpnt1[c] --> tpn1[c]
TPN2	tpnc2[c] + tpni2[c] + tpnt2[c] --> tpn2[c]
CONTR-COMP1	7 act[c] + 7 myo1[c] + dim-tmn1[c] + tpn1[c] --> contr-comp1[c]
CONTR-COMP2a	7 act[c] + 7 myo2a[c] + dim-tmn2[c] + tpn2[c] --> contr-comp2a[c]
CONTR-COMP2x	7 act[c] + 7 myo2x[c] + dim-tmn2[c] + tpn2[c] --> contr-comp2x[c]
CONTR-COMP2b	7 act[c] + 7 myo2b[c] + dim-tmn2[c] + tpn2[c] --> contr-comp2b[c]
Str_CONTR-COMP1	contr-comp1[c] -->
Str_CONTR-COMP2a	contr-comp2a[c] -->
Str_CONT-COMP2x	contr-comp2x[c] -->
Str_CONTR-COMP2b	contr-comp2b[c] -->
CKM	cr-p + adp <==> cr + atp
Spont	cr-p <==> cr + p
Nut_cr	cr <==>
ATP2UTP	atp --> utp
UDP2ADP	udp --> adp
PGM1	g6p[c] <==> g1p[c]
UGP2	g1p[c] + utp --> udp-g1p[c] + (2) p
mGYG	udp-g1p[c] <==> glycogen[c] + udp
mGYS1	glycogen[c] -->
G6PD	g6p[c] + nadp[c] <==> gluc-lac-6p[c] + nadph[c]
PGLS	gluc-lac-6p[c] + h2o <==> 6pgluconate[c]
PGD	6pgluconate[c] + nadp[c] <==> ru5p[c] + co2 + nadph[c]
PGD	6pgluconate[c] + nad[c] <==> ru5p[c] + co2 + nadh[c]
RPE	ru5p[c] <==> x5p[c]
RPIA	ru5p[c] <==> r5p[c]
TKT1	e4p[c] + x5p[c] <==> f6p[c] + g3p[c]
TKT2	s7p[c] + g3p[c] <==> r5p[c] + x5p[c]
TALDO	s7p[c] + g3p[c] <==> e4p[c] + f6p[c]
RBSK	rib[c] + atp <==> r5p[c] + adp + p
GCL	cys[c] + glu[c] + atp --> glu-cys[c] + p + adp
GSS	glu-cys[c] + gly[c] + atp --> glutath[c] + adp + p
glutathxport	glutath[c] <==> glutath[m]
GSR	2 glutath[c] + nadp[c] --> gssg[c] + nadph[c]
GSR	2 glutath[m] + nadp[m] --> gssg[m] + nadph[m]
GlutathLeak	gssg[c] -->
GlutathLeak	gssg[m] -->
Recycle_nadp	nadph[c] --> nadp[c]
fa-c6 leak	6c-3ketoacyl-coa[m] -->
acetyLeak(mit)	acetyl-coa[m] -->

lys	met
0	6.52058E-06
0	0
0	5
0	5
0	0
0	0
0	0.125
0	0
0	0
0	0
0	0
0	0
0	0
0	0
0	0
0	0.23
0	0
0	0
0	0
0	0
0	0
0	0
0	0
0	0
0	38.41780876
0	-36.0574855
0	-287.286786
0	-14.9999983
0	0.039775523
0	0.23
0	0
0	0
0	0
0	0
0	0
0	0
0	-5
0	0
0	0
0	-0.125
0	0
0	0
0	0
0	0
0	0
0	0

0	0
0	0
0	-0.23
0	0
0	0
0	-0.23
0	0
0	0
0	0
0	0
0	-5
0	-4.999995
0	-4.99999833
0	0
0	-4.99999833
0	4.999998333
0	-9.99999833
0	-9.99999833
0	-9.79823591
0	-9.79823591
0	-9.74687638
0	0.229204473
0	0
0	-0.17784495
0	0
0	-10.2318173
0	-10.4096623
0	0
0	-10.2318173
0	-10.9633417
0	-9.99999833
0	-10.9633417
0	0
0	-9.99999833
0	-12.5841021
0	-12.5841021
0	0
0	-12.5841021
0	-13.288703
0	-13.2901376
0	-13.3156265
0	-13.3156265
0	-23.5474438
0	-48.7454862
0	-28.0901313
0	-76.8356175
0	-38.4178088

0	-202.416721
0	-0.19219252
0	-0.125
0	-0.125
0	-0.125
0	-0.125
0	0
0	0
0	0
0	0
0	-0.125
0	-0.125
0	-0.125
0	-0.125
0	0
0	0
0	0
0	0
0	-0.355
0	-0.355
0	-0.355
0	-0.355
0	-0.355
0	-0.355
0	-0.355
0	-0.355
0	-0.355
0	-0.355
0	-0.355
0	-0.355
0	-0.355
0	-0.355
0	-0.355
0	-0.355
0	-0.355
0	-0.355
0	-0.355
0	-0.355
0	-0.355
0	-0.30941967
0	-0.30941967
0	-0.33490861
0	0
0	0
0	0
0	0
0	0

0	0
0	0
0	0
0	0
0	0
0	0
0	0
0	0
0	0
0	-0.23
0	0
0	0
0	0
0	0
0	0
0	0
0	0
0	0
0	0
0	-0.23
0	0
0	0
0	0
0	0
0	0
0	0
0	0
0	0
0	0
0	-0.23
0	0
0	0
0	0
0	0
0	0
0	0
0	0
0	0
0	0
0	-0.23
0	0
0	0
0	0
0	0
0	0
0	0
0	0

0	0
0	0
0	0
0	0
0	0
0	0
0	0
0	0
0	0
0	-0.01390839
0	-0.03483918
0	0
0	0
0	-0.07252335
0	-0.00748562
0	-0.00888755
0	-0.01417574
0	-0.00148017
0	0
0	0
0	-0.57815767
0	0
0	-0.00307771
0	0
0	0
0	-0.0441636
0	0
0	-0.18552646
0	-0.00520342
0	-0.0524124
0	-0.01263688
0	-0.01263688
0	-0.01263688
0	-0.01263688
0	-0.01263688
0	-0.01263688
0	-0.01263688
0	-0.01263688
0	-0.01263688
0	-0.02692346
0	-0.02692346
0	-0.02692346
0	-0.01428659
0	-0.01428659
0	-0.01428659
0	-0.01428659
0	-0.01428659

0	-0.01428659
0	-0.01428659
0	-0.02548894
0	-0.02548894
0	-0.02548894
0	-0.02548894
0	-0.02548894
0	-0.02548894
0	-0.02548894
0	-0.02548894
0	-0.02548894
0	0
0	0.06567172
0	0.125764828
0	0.677957554
0	0.023917478
0	0
0	0
0	0
0	0
0	0
0	0
0	0.045580329
0	0.045580329
0	0.045580329
0	0.045580329
0	0.045580329
0	0.045580329
0	0.045580329
0	-0.00852892
0	-12.5269593
0	-0.00852892
0	0
0	0
0	0
0	0
0	0
0	0
0	0
0	-0.03004655
0	0
0	0
0	0.030046554
0	0.030046554
0	0.030046554
0	0.030046554
0	0.030046554

0	0.030046554
0	0
0	-0.56777691
0	-0.20176243
0	-0.20176243
0	-0.20176243
0	0
0	0
0	0
0	-12.5184304
0	0
0	0
0	0
0	-0.03545238
0	-2.7535E-16
0	0.005203421
0	0.001480171
0	0.014175736
0	0.025488938
0	0.014286585
0	0.026943026
0	0.023917478
0	0.012636879
0	0.007485623
0	0.013810583
0	0.014097489
0	0.01411705
0	0.008528915
0	0.003077713
0	0.032576805
0	0.01558418
0	0.013908392
0	0.008887547
0	0.004792624
0	0.010380759
0	4.5644E-05
0	0
0	9.12881E-05
0	0
0	9.12881E-05
0	9.12881E-05
0	0
0	4.5644E-05
0	0
0	0
0	0
0	1.30412E-05

0	0
0	6.52058E-06
0	0
0	6.52058E-06
0	0
0	6.52058E-06
0	0
0	6.52058E-06
0	0
0	6.52058E-06
0	0
0	6.52058E-06
0	0
0	0
0	0
0	6.52058E-06
0	0
0	0
0	225.376275
0	-225.376275
0	6.99E-17
0	0
0	0
0	0
0	0
0	0
0	0
0	-0.000005
0	-0.000005
0	0.000005
0	-0.00001
0	-3.3333E-06
0	-1.6667E-06
0	-1.6667E-06
0	1.66667E-06
0	-1.6667E-06
0	0
0	0
0	0
0	0
0	0
0	0
0	0
0	0
0	0
0	0
0	-0.02548894

0	0

Flux	**control**	**arg**	**his**	**ile**	**leu**	**lys**	**met**	**phe**
**Prot Synth**	**2.18E-05**	**2.18E-05**	**2.18E-05**	**2.18E-05**	**2.18E-05**	**2.18E-05**	**2.83E-05**	**2.18E-05**
Obj_atp	0	0	0	0	0	0	0	0
Ex_ins	-5	-5	-5	-5	-5	-5	1.88E-18	-5
Ex_gluc	-5	-5	-5	-5	-5	-5	1.88E-18	-5
Ex_lac	0	0	0	0	0	0	0	0
Ex_pyr	0	0	0	0	0	0	0	0
Ex_pal	-0.125	-0.125	-0.125	-0.125	-0.125	-0.125	0	-0.125
Ex_prop	0	0	0	0	0	0	0	0
Ex_but	0	0	0	0	0	0	0	0
Ex_5c	0	0	0	0	0	0	0	0
Ex_6c	0	0	0	0	0	0	0	0
Ex_8c	0	0	0	0	0	0	0	0
Ex_9c	0	0	0	0	0	0	0	0
Ex_10c	0	0	0	0	0	0	0	0
Ex_12c	0	0	0	0	0	0	0	0
Ex_14c	-0.23	-0.23	-0.23	-0.23	-0.23	-0.23	0	-0.23
Ex_18c	0	0	0	0	0	0	0	0
Ex_glyc	0	0	0	0	0	0	0	0
Ex_tag	0	0	0	0	0	0	0	0
Ex_dag	0	0	0	0	0	0	0	0
Ex_mag	0	0	0	0	0	0	0	0
Ex_akg	0	0	0	0	0	0	0	0
Ex_oaa	0	0	0	0	0	0	0	0
Ex_rib	0	0	0	0	0	0	0	0
Nut_o2	-39.3733	-39.3733	-39.3733	-84.0943	-40.2626	-39.3733	-0.95554	-39.3733
Nut_co2	36.40954	36.40954	36.40954	76.16154	37.12092	36.40954	0.352055	36.40954
Nut_h2o	292.4733	292.4733	292.4733	610.4893	298.1643	292.4733	5.186478	292.4733
Nut_p	15	15	15	15	15	15	9.38E-16	15
Nut_hco3	-0.05216	-0.05216	-0.05216	-9.99016	-0.23001	-0.05216	-0.01238	-0.05216
Nut_coa	-0.23	-0.23	-0.23	-0.23	-0.23	-0.23	0	-0.23
Nut_carn	0	0	0	0	0	0	0	0
Nut_dhf	0	0	0	0	0	0	0	0
LPL	0	0	0	0	0	0	0	0
DAGL	0	0	0	0	0	0	0	0
12DAG	0	0	0	0	0	0	0	0
MAGL	0	0	0	0	0	0	0	0
GLUT4	5	5	5	5	5	5	-1.9E-18	5
MCT4	0	0	0	0	0	0	0	0
MCT_pyr	0	0	0	0	0	0	0	0
FAT/CD36	0.125	0.125	0.125	0.125	0.125	0.125	0	0.125
FATprop	0	0	0	0	0	0	0	0
FATbut	0	0	0	0	0	0	0	0
FAT5c	0	0	0	0	0	0	0	0
FAT6c	0	0	0	0	0	0	0	0
FAT8c	0	0	0	0	0	0	0	0
FAT9c	0	0	0	0	0	0	0	0

FAT10c	0	0	0	0	0	0	0	0
FAT12c	0	0	0	0	0	0	0	0
FAT14c	0.23	0.23	0.23	0.23	0.23	0.23	0	0.23
FAT18c	0	0	0	0	0	0	0	0
Alc_glyc	0	0	0	0	0	0	0	0
COA	0.23	0.23	0.23	0.23	0.23	0.23	0	0.23
SLC22A5/O	0	0	0	0	0	0	0	0
Xport_rib	0	0	0	0	0	0	0	0
Dr_akg	0	0	0	0	0	0	0	0
Dr_oaa	0	0	0	0	0	0	0	0
Hex	5	5	5	5	5	5	-1.9E-18	5
PGI	4.999995	4.999995	4.999995	4.999995	4.999995	4.999995	-1.9E-18	4.999995
PFK	4.999998	4.999998	4.999998	4.999998	4.999998	4.999998	-1.9E-18	4.999998
FBP	0	0	0	0	0	0	0	0
Ald	4.999998	4.999998	4.999998	4.999998	4.999998	4.999998	-1.9E-18	4.999998
TPI	-5	-5	-5	-5	-5	-5	1.88E-18	-5
D3PDeh	9.999998	9.999998	9.999998	9.999998	9.999998	9.999998	-3.8E-18	9.999998
PGK	9.999998	9.999998	9.999998	9.999998	9.999998	9.999998	-3.8E-18	9.999998
PGM	9.569031	9.569031	9.569031	-0.36897	9.746876	9.569031	-0.2292	9.569031
Eno	9.569031	9.569031	9.569031	-0.36897	9.746876	9.569031	-0.2292	9.569031
PyrK	9.746876	9.746876	9.746876	9.746876	9.746876	9.746876	0	9.746876
PEPSynth	0	0	0	0	0	0	0.229204	0
PEPCarbK	0	0	0	0	0	0	0	0
PEPCarbl	0.177845	0.177845	0.177845	10.11584	0	0.177845	0	0.177845
LacDeh	0	0	0	0	0	0	0	0
MalDehC(n	10.43098	10.43098	10.43098	20.36898	10.25313	10.43098	0.199158	10.43098
ASTC	10.60882	10.60882	10.60882	30.48482	10.25313	10.60882	0.199158	10.60882
G3PDehC	0	0	0	0	0	0	0	0
Mal-AKGEX	10.43098	10.43098	10.43098	20.36898	10.25313	10.43098	0.199158	10.43098
Glu-AspEx	11.22473	11.22473	11.22473	31.10073	11.04688	11.22473	0.261387	11.22473
MCT	9.999998	9.999998	9.999998	9.999998	9.999998	9.999998	0	9.999998
ASTM	11.22473	11.22473	11.22473	31.10073	11.04688	11.22473	0.261387	11.22473
G3PDehM	0	0	0	0	0	0	0	0
PyrSynth/P	9.999998	9.999998	9.999998	9.999998	9.999998	9.999998	0	9.999998
CitSynth	12.70003	12.70003	12.70003	22.63803	13.05572	12.70003	0.11593	12.70003
Acon	12.70003	12.70003	12.70003	22.63803	13.05572	12.70003	0.11593	12.70003
IsoDeh	0	0	0	0	0	0	0	0
IsoDehnad	12.70003	12.70003	12.70003	22.63803	13.05572	12.70003	0.11593	12.70003
AKGDeh	13.2887	13.2887	13.2887	23.2267	13.64439	13.2887	0	13.2887
SunCoaSyn	13.45591	13.45591	13.45591	33.33191	13.63376	13.45591	0.165775	13.45591
SucDeh(I)	13.49379	13.49379	13.49379	33.36979	13.84948	13.49379	0.178159	13.49379
Fum	13.49379	13.49379	13.49379	33.36979	13.84948	13.49379	0.178159	13.49379
MalDehM	23.92476	23.92476	23.92476	53.73876	24.10261	23.92476	0.377317	23.92476
NADHDeh(	49.89181	49.89181	49.89181	109.5198	50.60319	49.89181	1.146327	49.89181
ETFUO	28.85488	28.85488	28.85488	58.66888	29.92195	28.85488	0.764746	28.85488
UCytC(III)	78.74669	78.74669	78.74669	168.1887	80.52514	78.74669	1.911073	78.74669
CytCO(IV)	39.37335	39.37335	39.37335	84.09435	40.26257	39.37335	0.955537	39.37335

ATPSynth(\	207.3852	207.3852	207.3852	445.8972	211.6535	207.3852	4.968474	207.3852
AMP_ADPs	1.603164	1.603164	1.603164	1.603164	1.781009	1.603164	1.410971	1.603164
PalTK	0.125	0.125	0.125	0.125	0.125	0.125	0	0.125
CPT1B/CHK	0.125	0.125	0.125	0.125	0.125	0.125	0	0.125
SLC25A20/	0.125	0.125	0.125	0.125	0.125	0.125	0	0.125
CPT2	0.125	0.125	0.125	0.125	0.125	0.125	0	0.125
CRAT(mit)	0	0	0	0	0	0	0	0
CRAT Trans	0	0	0	0	0	0	0	0
CRAT(cyt)	0	0	0	0	0	0	0	0
Acetyl-coaL	0	0	0	0	0	0	0	0
AcylCoADe	0.125	0.125	0.125	0.125	0.125	0.125	0	0.125
EnoCoADel	0.125	0.125	0.125	0.125	0.125	0.125	0	0.125
3HA-CoADe	0.125	0.125	0.125	0.125	0.125	0.125	0	0.125
AcylCoAAT	0.125	0.125	0.125	0.125	0.125	0.125	0	0.125
AcylCoADe	0	0	0	0	0	0	0	0
EnoCoADel	0	0	0	0	0	0	0	0
3HA-CoADe	0	0	0	0	0	0	0	0
AcylCoAAT-	0	0	0	0	0	0	0	0
AcylCoADe	0.355	0.355	0.355	0.355	0.355	0.355	0	0.355
EnoCoADel	0.355	0.355	0.355	0.355	0.355	0.355	0	0.355
3HA-CoADe	0.355	0.355	0.355	0.355	0.355	0.355	0	0.355
AcylCoAAT-	0.355	0.355	0.355	0.355	0.355	0.355	0	0.355
AcylCoADe	0.355	0.355	0.355	0.355	0.355	0.355	0	0.355
EnoCoADel	0.355	0.355	0.355	0.355	0.355	0.355	0	0.355
3HA-CoADe	0.355	0.355	0.355	0.355	0.355	0.355	0	0.355
AcylCoAAT-	0.355	0.355	0.355	0.355	0.355	0.355	0	0.355
AcylCoADe	0.355	0.355	0.355	0.355	0.355	0.355	0	0.355
EnoCoADel	0.355	0.355	0.355	0.355	0.355	0.355	0	0.355
3HA-CoADe	0.355	0.355	0.355	0.355	0.355	0.355	0	0.355
AcylCoAAT-	0.355	0.355	0.355	0.355	0.355	0.355	0	0.355
AcylCoADe	0.355	0.355	0.355	0.355	0.355	0.355	0	0.355
EnoCoADel	0.355	0.355	0.355	0.355	0.355	0.355	0	0.355
3HA-CoADe	0.355	0.355	0.355	0.355	0.355	0.355	0	0.355
AcylCoAAT-	0.355	0.355	0.355	0.355	0.355	0.355	0	0.355
AcylCoADe	0.355	0.355	0.355	0.355	0.355	0.355	0	0.355
EnoCoADel	0.355	0.355	0.355	0.355	0.355	0.355	0	0.355
3HA-CoADe	0.355	0.355	0.355	0.355	0.355	0.355	0	0.355
AcylCoAAT-	0.355	0.355	0.355	0.355	0.355	0.355	0	0.355
ButyrlCoAD	0.355	0.355	0.355	0.355	0.355	0.355	0	0.355
EnoCoADel	0.355	0.355	0.355	0.355	0.355	0.355	0.04558	0.355
3HA-CoADe	0.355	0.355	0.355	0.355	0.355	0.355	0.04558	0.355
AcylCoAAT-	0.392874	0.392874	0.392874	0.392874	0.570719	0.392874	0.057965	0.392874
GK	0	0	0	0	0	0	0	0
GPAT4	0	0	0	0	0	0	0	0
ABHD5	0	0	0	0	0	0	0	0
DAGZ/DAG	0	0	0	0	0	0	0	0
DGAT1	0	0	0	0	0	0	0	0

St_TAG	0	0	0	0	0	0	0	0
ACAS2	0	0	0	0	0	0	0	0
ACSM-But	0	0	0	0	0	0	0	0
ASCM-5c	0	0	0	0	0	0	0	0
ASCM-6c	0	0	0	0	0	0	0	0
ACSM-8c	0	0	0	0	0	0	0	0
ACSM-9c	0	0	0	0	0	0	0	0
ACSM-10c	0	0	0	0	0	0	0	0
ACSM-12c	0	0	0	0	0	0	0	0
ACSM-14c	0.23	0.23	0.23	0.23	0.23	0.23	0	0.23
ACSM-18c	0	0	0	0	0	0	0	0
CPT1B/CHK	0	0	0	0	0	0	0	0
CPT1B/CHK	0	0	0	0	0	0	0	0
CPT1B/CHK	0	0	0	0	0	0	0	0
CPT1B/CHK	0	0	0	0	0	0	0	0
CPT1B/CHK	0	0	0	0	0	0	0	0
CPT1B/CHK	0	0	0	0	0	0	0	0
CPT1B/CHK	0	0	0	0	0	0	0	0
CPT1B/CHK	0	0	0	0	0	0	0	0
CPT1B/CHK	0.23	0.23	0.23	0.23	0.23	0.23	0	0.23
CPT1B/CHK	0	0	0	0	0	0	0	0
SLC25A20/	0	0	0	0	0	0	0	0
SLC25A20/	0	0	0	0	0	0	0	0
SLC25A20/	0	0	0	0	0	0	0	0
SLC25A20/	0	0	0	0	0	0	0	0
SLC25A20/	0	0	0	0	0	0	0	0
SLC25A20/	0	0	0	0	0	0	0	0
SLC25A20/	0	0	0	0	0	0	0	0
SLC25A20/	0	0	0	0	0	0	0	0
SLC25A20/	0.23	0.23	0.23	0.23	0.23	0.23	0	0.23
SLC25A20/	0	0	0	0	0	0	0	0
CPT2	0	0	0	0	0	0	0	0
CPT2	0	0	0	0	0	0	0	0
CPT2	0	0	0	0	0	0	0	0
CPT2	0	0	0	0	0	0	0	0
CPT2	0	0	0	0	0	0	0	0
CPT2	0	0	0	0	0	0	0	0
CPT2	0	0	0	0	0	0	0	0
CPT2	0	0	0	0	0	0	0	0
CPT2	0.23	0.23	0.23	0.23	0.23	0.23	0	0.23
CPT2	0	0	0	0	0	0	0	0
AcylCoADe	0	0	0	0	0	0	0	0
EnoCoADel	0	0	0	0	0	0	0	0
3HA-CoADe	0	0	0	0	0	0	0	0
AcylCoAAT	0	0	0	0	0	0	0	0
AcylCoADe	0	0	0	0	0	0	0	0
EnoCoADel	0	0	0	0	0	0	0	0

3HA-CoADe	0	0	0	0	0	0	0	0
AcylCoAAT-	0	0	0	0	0	0	0	0
AcylCoADe	0	0	0	0	0	0	0	0
EnoCoADel	0	0	0	0	0	0	0	0
3HA-CoADe	0	0	0	0	0	0	0	0
AcylCoAAT-	0	0	0	0	0	0	0	0
AKR1A1	0	0	0	0	0	0	0	0
ALDH3A2	0	0	0	0	0	0	0	0
GLYCTK	0	0	0	0	0	0	0	0
EAA_arg	-0.04645	-0.04645	-0.04645	-0.04645	-0.04645	-0.04645	-0.06036	-0.04645
EAA_his	-0.01601	-0.01601	-0.01601	-0.01601	-0.01601	-0.01601	-0.05085	-0.01601
EAA_ile	-0.062	-0.062	-0.062	-10	-0.062	-0.062	-0.062	-0.062
EAA_leu	-0.123	-0.123	-0.123	-0.123	-0.30084	-0.123	-0.123	-0.123
EAA_lys	-0.08998	-0.08998	-0.08998	-0.08998	-0.08998	-0.08998	-0.16251	-0.08998
EAA_met	-0.025	-0.025	-0.025	-0.025	-0.025	-0.025	-0.03249	-0.025
EAA_phe	-0.02968	-0.02968	-0.02968	-0.02968	-0.02968	-0.02968	-0.03857	-0.02968
EAA_thr	-0.04734	-0.04734	-0.04734	-0.04734	-0.04734	-0.04734	-0.06152	-0.04734
EAA_trp	-0.00494	-0.00494	-0.00494	-0.00494	-0.00494	-0.00494	-0.00642	-0.00494
EAA_val	-0.233	-0.233	-0.233	-0.233	-0.233	-0.233	-0.233	-0.233
NEAA_ala	-0.333	-0.333	-0.333	-0.333	-0.333	-0.333	-0.333	-0.333
NEAA_asn	0.537158	0.537158	0.537158	0.537158	0.715003	0.537158	-0.041	0.537158
NEAA_asp	-0.003	-0.003	-0.003	-0.003	-0.003	-0.003	-0.003	-0.003
NEAA_cys	-0.01028	-0.01028	-0.01028	-0.01028	-0.01028	-0.01028	-0.01336	-0.01028
NEAA_glu	-0.024	-0.024	-0.024	-0.024	-0.024	-0.024	-0.024	-0.024
NEAA_gln	-0.586	-0.586	-0.586	-0.586	-0.586	-0.586	-0.586	-0.586
NEAA_gly	-0.04715	-0.04715	-0.04715	-0.04715	-0.04715	-0.04715	-0.09131	-0.04715
NEAA_pro	-0.168	-0.168	-0.168	-0.168	-0.168	-0.168	-0.168	-0.168
NEAA_ser	0.384843	0.384843	0.384843	10.32284	0.206998	0.384843	0.199317	0.384843
NEAA_tyr	-0.01738	-0.01738	-0.01738	-0.01738	-0.01738	-0.01738	-0.02258	-0.01738
DLDH	0.242956	0.242956	0.242956	10.18096	0.420801	0.242956	0.190544	0.242956
MT_val	0.190796	0.190796	0.190796	0.190796	0.190796	0.190796	0.178159	0.190796
BCAT2-val	0.190796	0.190796	0.190796	0.190796	0.190796	0.190796	0.178159	0.190796
BCKDHB-va	0.190796	0.190796	0.190796	0.190796	0.190796	0.190796	0.178159	0.190796
BCKADE2-v	0.190796	0.190796	0.190796	0.190796	0.190796	0.190796	0.178159	0.190796
ACADSB	0.190796	0.190796	0.190796	0.190796	0.190796	0.190796	0.178159	0.190796
ECHS1	0.190796	0.190796	0.190796	0.190796	0.190796	0.190796	0.178159	0.190796
HIBCH	0.190796	0.190796	0.190796	0.190796	0.190796	0.190796	0.178159	0.190796
HIBADH	0.190796	0.190796	0.190796	0.190796	0.190796	0.190796	0.178159	0.190796
ALDH6A1	0.190796	0.190796	0.190796	0.190796	0.190796	0.190796	0.178159	0.190796
PCCB	0.205083	0.205083	0.205083	10.14308	0.205083	0.205083	0.178159	0.205083
MCEE	0.205083	0.205083	0.205083	10.14308	0.205083	0.205083	0.178159	0.205083
MUT	0.205083	0.205083	0.205083	10.14308	0.205083	0.205083	0.178159	0.205083
MT_ile	0.014287	0.014287	0.014287	9.952287	0.014287	0.014287	0	0.014287
BCAT-ile	0.014287	0.014287	0.014287	9.952287	0.014287	0.014287	0	0.014287
BCKDHB-ile	0.014287	0.014287	0.014287	9.952287	0.014287	0.014287	0	0.014287
BCKADE2-il	0.014287	0.014287	0.014287	9.952287	0.014287	0.014287	0	0.014287
ECHS1	0.014287	0.014287	0.014287	9.952287	0.014287	0.014287	0	0.014287

HSD17B10	0.014287	0.014287	0.014287	9.952287	0.014287	0.014287	0	0.014287
ACAT1	0.014287	0.014287	0.014287	9.952287	0.014287	0.014287	0	0.014287
MT_leu	0.037874	0.037874	0.037874	0.037874	0.215719	0.037874	0.012385	0.037874
BCAT2-leu	0.037874	0.037874	0.037874	0.037874	0.215719	0.037874	0.012385	0.037874
BCKDHB-le	0.037874	0.037874	0.037874	0.037874	0.215719	0.037874	0.012385	0.037874
BCKADE2-l	0.037874	0.037874	0.037874	0.037874	0.215719	0.037874	0.012385	0.037874
IVD	0.037874	0.037874	0.037874	0.037874	0.215719	0.037874	0.012385	0.037874
MCC1,MCC	0.037874	0.037874	0.037874	0.037874	0.215719	0.037874	0.012385	0.037874
MGCA	0.037874	0.037874	0.037874	0.037874	0.215719	0.037874	0.012385	0.037874
HMGCL	0.037874	0.037874	0.037874	0.037874	0.215719	0.037874	0.012385	0.037874
OXCT	0.037874	0.037874	0.037874	0.037874	0.215719	0.037874	0.012385	0.037874
HMGCS2	0	0	0	0	0	0	0	0
GluDeh	0.037874	0.037874	0.037874	0.037874	0.215719	0.037874	0.103545	0.037874
GlnSynth	0.037874	0.037874	0.037874	0.037874	0.215719	0.037874	0.163639	0.037874
GLN_Elim	0	0	0	0	0	0	0.677958	0
ALT	-0.25312	-0.25312	-0.25312	-0.25312	-0.25312	-0.25312	-0.2292	-0.25312
ALA_Elim	0	0	0	0	0	0	0	0
SDS	0	0	0	0	0	0	0	0
CBS	0	0	0	0	0	0	0	0
CTH	0	0	0	0	0	0	0	0
ILVBL	0	0	0	0	0	0	0	0
2a2hButLe	0	0	0	0	0	0	0	0
MT_lys	0	0	0	0	0	0	0.04558	0
AASS	0	0	0	0	0	0	0.04558	0
AASS	0	0	0	0	0	0	0.04558	0
aAASADeh	0	0	0	0	0	0	0.04558	0
KATII	0	0	0	0	0	0	0.04558	0
lysdeg	0	0	0	0	0	0	0.04558	0
GCDH	0	0	0	0	0	0	0.04558	0
MT_pro	0.139516	0.139516	0.139516	0.139516	0.139516	0.139516	0.130987	0.139516
PRODH	12.87742	12.87742	12.87742	22.81542	13.41096	12.87742	0.350462	12.87742
ALDH4a1	0.139516	0.139516	0.139516	0.139516	0.139516	0.139516	0.130987	0.139516
GATM	0	0	0	0	0	0	0	0
Dr_orn	0	0	0	0	0	0	0	0
GAMT	0	0	0	0	0	0	0	0
Dr_cr	0	0	0	0	0	0	0	0
AdoHcyase	0	0	0	0	0	0	0	0
MTR	0	0	0	0	0	0	0	0
AdoMet	0	0	0	0	0	0	0	0
SHMT	0	0	0	0	0	0	-0.03005	0
MTHFR	0	0	0	0	0	0	0	0
DHFR	0	0	0	0	0	0	0	0
HAL	0	0	0	0	0	0	0.030047	0
FLJ31300	0	0	0	0	0	0	0.030047	0
MGC35366	0	0	0	0	0	0	0.030047	0
FTCD1	0	0	0	0	0	0	0.030047	0
FTCD2	0	0	0	0	0	0	0.030047	0

FT	0	0	0	0	0	0	0.030047	0
ASNase	0	0	0	0	0	0	0	0
ASNSynth	0.571827	0.571827	0.571827	0.571827	0.749672	0.571827	0.00405	0.571827
PHGDH	0.430967	0.430967	0.430967	10.36897	0.253122	0.430967	0.229204	0.430967
PSA	0.430967	0.430967	0.430967	10.36897	0.253122	0.430967	0.229204	0.430967
PSPH	0.430967	0.430967	0.430967	10.36897	0.253122	0.430967	0.229204	0.430967
P5CS	0	0	0	0	0	0	0	0
PYCS	0	0	0	0	0	0	0	0
Spont	0	0	0	0	0	0	0	0
PYRCR1	12.73791	12.73791	12.73791	22.67591	13.27144	12.73791	0.219476	12.73791
PAG	0	0	0	0	0	0	0	0
PCBD	0	0	0	0	0	0	0	0
QDPR	0	0	0	0	0	0	0	0
MT_glu	0.344598	0.344598	0.344598	10.2826	0.344598	0.344598	0.309146	0.344598
tRNA	1.22E-16	1.22E-16	8.68E-17	1.24E-16	4.28E-17	1.22E-16	-1.5E-16	1.22E-16
YARS	0.017378	0.017378	0.017378	0.017378	0.017378	0.017378	0.022581	0.017378
WARS	0.004943	0.004943	0.004943	0.004943	0.004943	0.004943	0.006424	0.004943
TARS	0.047343	0.047343	0.047343	0.047343	0.047343	0.047343	0.061519	0.047343
LARS	0.085126	0.085126	0.085126	0.085126	0.085126	0.085126	0.110615	0.085126
IARS	0.047713	0.047713	0.047713	0.047713	0.047713	0.047713	0.062	0.047713
KARS	0.089983	0.089983	0.089983	0.089983	0.089983	0.089983	0.116926	0.089983
AARS	0.079878	0.079878	0.079878	0.079878	0.079878	0.079878	0.103796	0.079878
VARS	0.042204	0.042204	0.042204	0.042204	0.042204	0.042204	0.054841	0.042204
MARS	0.025	0.025	0.025	0.025	0.025	0.025	0.032486	0.025
SARS	0.046124	0.046124	0.046124	0.046124	0.046124	0.046124	0.059934	0.046124
DARS	0.047082	0.047082	0.047082	0.047082	0.047082	0.047082	0.061179	0.047082
GARS	0.047147	0.047147	0.047147	0.047147	0.047147	0.047147	0.061264	0.047147
PARS	0.028484	0.028484	0.028484	0.028484	0.028484	0.028484	0.037013	0.028484
CARS	0.010279	0.010279	0.010279	0.010279	0.010279	0.010279	0.013356	0.010279
EPRS	0.108798	0.108798	0.108798	0.108798	0.108798	0.108798	0.141375	0.108798
QARS	0.052047	0.052047	0.052047	0.052047	0.052047	0.052047	0.067631	0.052047
RARS	0.04645	0.04645	0.04645	0.04645	0.04645	0.04645	0.060359	0.04645
FARS	0.029682	0.029682	0.029682	0.029682	0.029682	0.029682	0.03857	0.029682
HARS	0.016006	0.016006	0.016006	0.016006	0.016006	0.016006	0.020799	0.016006
NARS	0.034669	0.034669	0.034669	0.034669	0.034669	0.034669	0.04505	0.034669
ACT	0.000152	0.000152	0.000152	0.000152	0.000152	0.000152	0.000198	0.000152
MYOHC1-2	0	0	0	0	0	0	0	0
MYOHC2a	0.000305	0.000305	0.000305	0.000305	0.000305	0.000305	0.000396	0.000305
MYOHC2b	0	0	0	0	0	0	0	0
MYOLCk	0.000305	0.000305	0.000305	0.000305	0.000305	0.000305	0.000396	0.000305
MYOLCp	0.000305	0.000305	0.000305	0.000305	0.000305	0.000305	0.000396	0.000305
MYO1-2x	0	0	0	0	0	0	0	0
MYO2a	0.000152	0.000152	0.000152	0.000152	0.000152	0.000152	0.000198	0.000152
MYO2x	0	0	0	0	0	0	0	0
MYO2b	0	0	0	0	0	0	0	0
TMN1	0	0	0	0	0	0	0	0
TMN2	4.36E-05	4.36E-05	4.36E-05	4.36E-05	4.36E-05	4.36E-05	5.66E-05	4.36E-05

DIM-TMN1	0	0	0	0	0	0	0	0
DIM-TMN2	2.18E-05	2.18E-05	2.18E-05	2.18E-05	2.18E-05	2.18E-05	2.83E-05	2.18E-05
TPNC1	0	0	0	0	0	0	0	0
TPNC2	2.18E-05	2.18E-05	2.18E-05	2.18E-05	2.18E-05	2.18E-05	2.83E-05	2.18E-05
TPNI1	0	0	0	0	0	0	0	0
TPNI2	2.18E-05	2.18E-05	2.18E-05	2.18E-05	2.18E-05	2.18E-05	2.83E-05	2.18E-05
TPNT1	0	0	0	0	0	0	0	0
TPNT2	2.18E-05	2.18E-05	2.18E-05	2.18E-05	2.18E-05	2.18E-05	2.83E-05	2.18E-05
TPN1	0	0	0	0	0	0	0	0
TPN2	2.18E-05	2.18E-05	2.18E-05	2.18E-05	2.18E-05	2.18E-05	2.83E-05	2.18E-05
CONTR-COI	0	0	0	0	0	0	0	0
CONTR-COI	2.18E-05	2.18E-05	2.18E-05	2.18E-05	2.18E-05	2.18E-05	2.83E-05	2.18E-05
CONTR-COI	0	0	0	0	0	0	0	0
CONTR-COI	0	0	0	0	0	0	0	0
Str_CONTR	0	0	0	0	0	0	0	0
Str_CONTR	2.18E-05	2.18E-05	2.18E-05	2.18E-05	2.18E-05	2.18E-05	2.83E-05	2.18E-05
Str_CONT-(	0	0	0	0	0	0	0	0
Str_CONTR	0	0	0	0	0	0	0	0
CKM	-227.568	-227.568	-227.568	-476.018	-231.48	-227.568	-2.19138	-227.568
Spont	227.5677	227.5677	227.5677	476.0177	231.4802	227.5677	2.191377	227.5677
Nut_cr	0	0	0	0	0	0	6.99E-17	0
ATP2UTP	0	0	0	0	0	0	0	0
UDP2ADP	0	0	0	0	0	0	0	0
PGM1	0	0	0	0	0	0	0	0
UGP2	0	0	0	0	0	0	0	0
mGYG	0	0	0	0	0	0	0	0
mGYS1	0	0	0	0	0	0	0	0
G6PD	0.000005	0.000005	0.000005	0.000005	0.000005	0.000005	0	0.000005
PGLS	0.000005	0.000005	0.000005	0.000005	0.000005	0.000005	0	0.000005
PGD	-5E-06	-5E-06	-5E-06	-5E-06	-5E-06	-5E-06	0	-5E-06
PGD	0.00001	0.00001	0.00001	0.00001	0.00001	0.00001	0	0.00001
RPE	3.33E-06	3.33E-06	3.33E-06	3.33E-06	3.33E-06	3.33E-06	0	3.33E-06
RPIA	1.67E-06	1.67E-06	1.67E-06	1.67E-06	1.67E-06	1.67E-06	0	1.67E-06
TKT1	1.67E-06	1.67E-06	1.67E-06	1.67E-06	1.67E-06	1.67E-06	0	1.67E-06
TKT2	-1.7E-06	-1.7E-06	-1.7E-06	-1.7E-06	-1.7E-06	-1.7E-06	0	-1.7E-06
TALDO	1.67E-06	1.67E-06	1.67E-06	1.67E-06	1.67E-06	1.67E-06	0	1.67E-06
RBSK	0	0	0	0	0	0	0	0
GCL	0	0	0	0	0	0	0	0
GSS	0	0	0	0	0	0	0	0
glutathxpoı	0	0	0	0	0	0	0	0
GSR	0	0	0	0	0	0	0	0
GSR	0	0	0	0	0	0	0	0
GlutathLea	0	0	0	0	0	0	0	0
GlutathLea	0	0	0	0	0	0	0	0
Recycle_na	0	0	0	0	0	0	0	0
fa-c6 leak	0	0	0	0	0	0	0	0
acetyLeak(ı	0.037874	0.037874	0.037874	0.037874	0.215719	0.037874	0.012385	0.037874

0	0	0	0	0	0	0	0

thr	trp	val	ala	asn	asp	cys	glu	gln
**2.18E-05**	**2.18E-05**	**2.18E-05**	**2.18E-05**	**2.18E-05**	**2.18E-05**	**2.18E-05**	**2.18E-05**	**2.18E-05**
0	0	0	0	0	0	0	0	0
-5	-5	-5	-5	-5	-5	-5	-5	-5
-5	-5	-5	-5	-5	-5	-5	-5	-5
0	0	0	0	0	0	0	0	0
0	0	0	0	0	0	0	0	0
-0.125	-0.125	-0.125	-0.125	-0.125	-0.125	-0.125	-0.125	-0.125
0	0	0	0	0	0	0	0	0
0	0	0	0	0	0	0	0	0
0	0	0	0	0	0	0	0	0
0	0	0	0	0	0	0	0	0
0	0	0	0	0	0	0	0	0
0	0	0	0	0	0	0	0	0
0	0	0	0	0	0	0	0	0
0	0	0	0	0	0	0	0	0
-0.23	-0.23	-0.23	-0.23	-0.23	-0.23	-0.23	-0.23	-0.23
0	0	0	0	0	0	0	0	0
0	0	0	0	0	0	0	0	0
0	0	0	0	0	0	0	0	0
0	0	0	0	0	0	0	0	0
0	0	0	0	0	0	0	0	0
0	0	0	0	0	0	0	0	0
0	0	0	0	0	0	0	0	0
0	0	0	0	0	0	0	0	0
-39.3733	-39.3733	-73.5578	-44.2068	-39.3733	-44.3718	-39.3733	-59.3253	-53.4943
36.40954	36.40954	55.94354	36.40954	36.40954	46.40654	36.40954	56.36154	45.82354
292.4733	292.4733	517.1143	311.8073	292.4733	322.4643	292.4733	442.1133	396.0273
15	15	15	15	15	15	15	15	15
-0.05216	-0.05216	-0.05216	-0.05216	-0.05216	-0.05216	-0.05216	-0.05216	-0.05216
-0.23	-0.23	-0.23	-0.23	-0.23	-0.23	-0.23	-0.23	-0.23
0	0	0	0	0	0	0	0	0
0	0	0	0	0	0	0	0	0
0	0	0	0	0	0	0	0	0
0	0	0	0	0	0	0	0	0
0	0	0	0	0	0	0	0	0
0	0	0	0	0	0	0	0	0
5	5	5	5	5	5	5	5	5
0	0	0	0	0	0	0	0	0
0	0	0	0	0	0	0	0	0
0.125	0.125	0.125	0.125	0.125	0.125	0.125	0.125	0.125
0	0	0	0	0	0	0	0	0
0	0	0	0	0	0	0	0	0
0	0	0	0	0	0	0	0	0
0	0	0	0	0	0	0	0	0
0	0	0	0	0	0	0	0	0
0	0	0	0	0	0	0	0	0

0	0	0	0	0	0	0	0	0
0	0	0	0	0	0	0	0	0
0.23	0.23	0.23	0.23	0.23	0.23	0.23	0.23	0.23
0	0	0	0	0	0	0	0	0
0	0	0	0	0	0	0	0	0
0.23	0.23	0.23	0.23	0.23	0.23	0.23	0.23	0.23
0	0	0	0	0	0	0	0	0
0	0	0	0	0	0	0	0	0
0	0	0	0	0	0	0	0	0
0	0	0	0	0	0	0	0	0
5	5	5	5	5	5	5	5	5
4.999995	4.999995	5	4.999995	4.999995	5	4.999995	4.999995	4.999995
4.999998	4.999998	5	4.999998	4.999998	5	4.999998	4.999998	4.999998
0	0	0	0	0	0	0	0	0
4.999998	4.999998	5	4.999998	4.999998	5	4.999998	4.999998	4.999998
-5	-5	-5	-5	-5	-5	-5	-5	-5
9.999998	9.999998	10	9.999998	9.999998	10	9.999998	9.999998	9.999998
9.999998	9.999998	10	9.999998	9.999998	10	9.999998	9.999998	9.999998
9.569031	9.569031	-0.19797	-0.09797	9.569031	-0.42797	9.569031	-0.40697	9.569031
9.569031	9.569031	-0.19797	-0.09797	9.569031	-0.42797	9.569031	-0.40697	9.569031
9.746876	9.746876	9.746878	0.079876	9.746876	9.746878	9.746876	9.746876	9.746876
0	0	0	0	0	0	0	0	0
0	0	0	0	0	0	0	0	0
0.177845	0.177845	9.944845	0.177845	0.177845	10.17484	0.177845	10.15384	0.177845
0	0	0	0	0	0	0	0	0
10.43098	10.43098	20.19797	20.09798	10.43098	20.42797	10.43098	20.40698	10.43098
10.60882	10.60882	30.14281	20.27582	10.60882	30.60281	10.60882	30.56082	10.60882
0	0	0	0	0	0	0	0	0
10.43098	10.43098	20.19797	20.09798	10.43098	20.42797	10.43098	20.40698	10.43098
11.22473	11.22473	30.75872	20.89173	11.22473	21.22172	11.22473	31.17673	20.63873
9.999998	9.999998	10	9.999998	9.999998	10	9.999998	9.999998	9.999998
11.22473	11.22473	30.75872	20.89173	11.22473	21.22172	11.22473	31.17673	20.63873
0	0	0	0	0	0	0	0	0
9.999998	9.999998	10	9.999998	9.999998	10	9.999998	9.999998	9.999998
12.70003	12.70003	12.70003	12.70003	12.70003	12.70003	12.70003	12.70003	12.70003
12.70003	12.70003	12.70003	12.70003	12.70003	12.70003	12.70003	12.70003	12.70003
0	0	0	0	0	0	0	0	0
12.70003	12.70003	12.70003	12.70003	12.70003	12.70003	12.70003	12.70003	12.70003
13.2887	13.2887	13.2887	13.2887	13.2887	13.2887	13.2887	23.2647	22.7027
13.45591	13.45591	23.22291	13.45591	13.45591	13.45591	13.45591	23.43191	22.86991
13.49379	13.49379	23.26079	13.49379	13.49379	13.49379	13.49379	23.46979	22.90779
13.49379	13.49379	23.26079	13.49379	13.49379	13.49379	13.49379	23.46979	22.90779
23.92476	23.92476	43.45875	33.59176	23.92476	33.92175	23.92476	43.87676	33.33876
49.89181	49.89181	98.72681	59.55881	49.89181	59.88881	49.89181	79.81981	68.71981
28.85488	28.85488	48.38888	28.85488	28.85488	28.85488	28.85488	38.83088	38.26888
78.74669	78.74669	147.1157	88.41369	78.74669	88.74369	78.74669	118.6507	106.9887
39.37335	39.37335	73.55785	44.20685	39.37335	44.37185	39.37335	59.32535	53.49435

207.3852	207.3852	392.9582	236.3862	207.3852	237.3762	207.3852	317.1212	282.6972
1.603164	1.603164	1.603164	1.603164	1.603164	1.603164	1.603164	1.603164	11.01716
0.125	0.125	0.125	0.125	0.125	0.125	0.125	0.125	0.125
0.125	0.125	0.125	0.125	0.125	0.125	0.125	0.125	0.125
0.125	0.125	0.125	0.125	0.125	0.125	0.125	0.125	0.125
0.125	0.125	0.125	0.125	0.125	0.125	0.125	0.125	0.125
0	0	0	0	0	0	0	0	0
0	0	0	0	0	0	0	0	0
0	0	0	0	0	0	0	0	0
0	0	0	0	0	0	0	0	0
0.125	0.125	0.125	0.125	0.125	0.125	0.125	0.125	0.125
0.125	0.125	0.125	0.125	0.125	0.125	0.125	0.125	0.125
0.125	0.125	0.125	0.125	0.125	0.125	0.125	0.125	0.125
0.125	0.125	0.125	0.125	0.125	0.125	0.125	0.125	0.125
0	0	0	0	0	0	0	0	0
0	0	0	0	0	0	0	0	0
0	0	0	0	0	0	0	0	0
0	0	0	0	0	0	0	0	0
0.355	0.355	0.355	0.355	0.355	0.355	0.355	0.355	0.355
0.355	0.355	0.355	0.355	0.355	0.355	0.355	0.355	0.355
0.355	0.355	0.355	0.355	0.355	0.355	0.355	0.355	0.355
0.355	0.355	0.355	0.355	0.355	0.355	0.355	0.355	0.355
0.355	0.355	0.355	0.355	0.355	0.355	0.355	0.355	0.355
0.355	0.355	0.355	0.355	0.355	0.355	0.355	0.355	0.355
0.355	0.355	0.355	0.355	0.355	0.355	0.355	0.355	0.355
0.355	0.355	0.355	0.355	0.355	0.355	0.355	0.355	0.355
0.355	0.355	0.355	0.355	0.355	0.355	0.355	0.355	0.355
0.355	0.355	0.355	0.355	0.355	0.355	0.355	0.355	0.355
0.355	0.355	0.355	0.355	0.355	0.355	0.355	0.355	0.355
0.355	0.355	0.355	0.355	0.355	0.355	0.355	0.355	0.355
0.355	0.355	0.355	0.355	0.355	0.355	0.355	0.355	0.355
0.355	0.355	0.355	0.355	0.355	0.355	0.355	0.355	0.355
0.355	0.355	0.355	0.355	0.355	0.355	0.355	0.355	0.355
0.355	0.355	0.355	0.355	0.355	0.355	0.355	0.355	0.355
0.355	0.355	0.355	0.355	0.355	0.355	0.355	0.355	0.355
0.355	0.355	0.355	0.355	0.355	0.355	0.355	0.355	0.355
0.355	0.355	0.355	0.355	0.355	0.355	0.355	0.355	0.355
0.355	0.355	0.355	0.355	0.355	0.355	0.355	0.355	0.355
0.355	0.355	0.355	0.355	0.355	0.355	0.355	0.355	0.355
0.355	0.355	0.355	0.355	0.355	0.355	0.355	0.355	0.355
0.355	0.355	0.355	0.355	0.355	0.355	0.355	0.355	0.355
0.392874	0.392874	0.392874	0.392874	0.392874	0.392874	0.392874	0.392874	0.392874
0	0	0	0	0	0	0	0	0
0	0	0	0	0	0	0	0	0
0	0	0	0	0	0	0	0	0
0	0	0	0	0	0	0	0	0
0	0	0	0	0	0	0	0	0

0	0	0	0	0	0	0	0	0
0	0	0	0	0	0	0	0	0
0	0	0	0	0	0	0	0	0
0	0	0	0	0	0	0	0	0
0	0	0	0	0	0	0	0	0
0	0	0	0	0	0	0	0	0
0	0	0	0	0	0	0	0	0
0	0	0	0	0	0	0	0	0
0	0	0	0	0	0	0	0	0
0.23	0.23	0.23	0.23	0.23	0.23	0.23	0.23	0.23
0	0	0	0	0	0	0	0	0
0	0	0	0	0	0	0	0	0
0	0	0	0	0	0	0	0	0
0	0	0	0	0	0	0	0	0
0	0	0	0	0	0	0	0	0
0	0	0	0	0	0	0	0	0
0	0	0	0	0	0	0	0	0
0	0	0	0	0	0	0	0	0
0	0	0	0	0	0	0	0	0
0.23	0.23	0.23	0.23	0.23	0.23	0.23	0.23	0.23
0	0	0	0	0	0	0	0	0
0	0	0	0	0	0	0	0	0
0	0	0	0	0	0	0	0	0
0	0	0	0	0	0	0	0	0
0	0	0	0	0	0	0	0	0
0	0	0	0	0	0	0	0	0
0	0	0	0	0	0	0	0	0
0	0	0	0	0	0	0	0	0
0	0	0	0	0	0	0	0	0
0.23	0.23	0.23	0.23	0.23	0.23	0.23	0.23	0.23
0	0	0	0	0	0	0	0	0
0	0	0	0	0	0	0	0	0
0	0	0	0	0	0	0	0	0
0	0	0	0	0	0	0	0	0
0	0	0	0	0	0	0	0	0
0	0	0	0	0	0	0	0	0
0	0	0	0	0	0	0	0	0
0	0	0	0	0	0	0	0	0
0	0	0	0	0	0	0	0	0
0.23	0.23	0.23	0.23	0.23	0.23	0.23	0.23	0.23
0	0	0	0	0	0	0	0	0
0	0	0	0	0	0	0	0	0
0	0	0	0	0	0	0	0	0
0	0	0	0	0	0	0	0	0
0	0	0	0	0	0	0	0	0
0	0	0	0	0	0	0	0	0
0	0	0	0	0	0	0	0	0

0	0	0	0	0	0	0	0	0
0	0	0	0	0	0	0	0	0
0	0	0	0	0	0	0	0	0
0	0	0	0	0	0	0	0	0
0	0	0	0	0	0	0	0	0
0	0	0	0	0	0	0	0	0
0	0	0	0	0	0	0	0	0
0	0	0	0	0	0	0	0	0
0	0	0	0	0	0	0	0	0
-0.04645	-0.04645	-0.04645	-0.04645	-0.04645	-0.04645	-0.04645	-0.04645	-0.04645
-0.01601	-0.01601	-0.01601	-0.01601	-0.01601	-0.01601	-0.01601	-0.01601	-0.01601
-0.062	-0.062	-0.062	-0.062	-0.062	-0.062	-0.062	-0.062	-0.062
-0.123	-0.123	-0.123	-0.123	-0.123	-0.123	-0.123	-0.123	-0.123
-0.08998	-0.08998	-0.08998	-0.08998	-0.08998	-0.08998	-0.08998	-0.08998	-0.08998
-0.025	-0.025	-0.025	-0.025	-0.025	-0.025	-0.025	-0.025	-0.025
-0.02968	-0.02968	-0.02968	-0.02968	-0.02968	-0.02968	-0.02968	-0.02968	-0.02968
-0.04734	-0.04734	-0.04734	-0.04734	-0.04734	-0.04734	-0.04734	-0.04734	-0.04734
-0.00494	-0.00494	-0.00494	-0.00494	-0.00494	-0.00494	-0.00494	-0.00494	-0.00494
-0.233	-0.233	-10	-0.233	-0.233	-0.233	-0.233	-0.233	-0.233
-0.333	-0.333	-0.333	-10	-0.333	-0.333	-0.333	-0.333	-0.333
0.537158	0.537158	0.537158	0.537158	0.537158	0.537158	0.537158	0.537158	9.951158
-0.003	-0.003	-0.003	-0.003	-0.003	-10	-0.003	-0.003	-0.003
-0.01028	-0.01028	-0.01028	-0.01028	-0.01028	-0.01028	-0.01028	-0.01028	-0.01028
-0.024	-0.024	-0.024	-0.024	-0.024	-0.024	-0.024	-10	-0.024
-0.586	-0.586	-0.586	-0.586	-0.586	-0.586	-0.586	-0.586	-10
-0.04715	-0.04715	-0.04715	-0.04715	-0.04715	-0.04715	-0.04715	-0.04715	-0.04715
-0.168	-0.168	-0.168	-0.168	-0.168	-0.168	-0.168	-0.168	-0.168
0.384843	0.384843	10.15184	10.05184	0.384843	10.38184	0.384843	10.36084	0.384843
-0.01738	-0.01738	-0.01738	-0.01738	-0.01738	-0.01738	-0.01738	-0.01738	-0.01738
0.242956	0.242956	10.00996	0.242956	0.242956	0.242956	0.242956	0.242956	0.242956
0.190796	0.190796	9.957796	0.190796	0.190796	0.190796	0.190796	0.190796	0.190796
0.190796	0.190796	9.957796	0.190796	0.190796	0.190796	0.190796	0.190796	0.190796
0.190796	0.190796	9.957796	0.190796	0.190796	0.190796	0.190796	0.190796	0.190796
0.190796	0.190796	9.957796	0.190796	0.190796	0.190796	0.190796	0.190796	0.190796
0.190796	0.190796	9.957796	0.190796	0.190796	0.190796	0.190796	0.190796	0.190796
0.190796	0.190796	9.957796	0.190796	0.190796	0.190796	0.190796	0.190796	0.190796
0.190796	0.190796	9.957796	0.190796	0.190796	0.190796	0.190796	0.190796	0.190796
0.190796	0.190796	9.957796	0.190796	0.190796	0.190796	0.190796	0.190796	0.190796
0.190796	0.190796	9.957796	0.190796	0.190796	0.190796	0.190796	0.190796	0.190796
0.205083	0.205083	9.972083	0.205083	0.205083	0.205083	0.205083	0.205083	0.205083
0.205083	0.205083	9.972083	0.205083	0.205083	0.205083	0.205083	0.205083	0.205083
0.205083	0.205083	9.972083	0.205083	0.205083	0.205083	0.205083	0.205083	0.205083
0.014287	0.014287	0.014287	0.014287	0.014287	0.014287	0.014287	0.014287	0.014287
0.014287	0.014287	0.014287	0.014287	0.014287	0.014287	0.014287	0.014287	0.014287
0.014287	0.014287	0.014287	0.014287	0.014287	0.014287	0.014287	0.014287	0.014287
0.014287	0.014287	0.014287	0.014287	0.014287	0.014287	0.014287	0.014287	0.014287
0.014287	0.014287	0.014287	0.014287	0.014287	0.014287	0.014287	0.014287	0.014287

0.014287	0.014287	0.014287	0.014287	0.014287	0.014287	0.014287	0.014287	0.014287
0.014287	0.014287	0.014287	0.014287	0.014287	0.014287	0.014287	0.014287	0.014287
0.037874	0.037874	0.037874	0.037874	0.037874	0.037874	0.037874	0.037874	0.037874
0.037874	0.037874	0.037874	0.037874	0.037874	0.037874	0.037874	0.037874	0.037874
0.037874	0.037874	0.037874	0.037874	0.037874	0.037874	0.037874	0.037874	0.037874
0.037874	0.037874	0.037874	0.037874	0.037874	0.037874	0.037874	0.037874	0.037874
0.037874	0.037874	0.037874	0.037874	0.037874	0.037874	0.037874	0.037874	0.037874
0.037874	0.037874	0.037874	0.037874	0.037874	0.037874	0.037874	0.037874	0.037874
0.037874	0.037874	0.037874	0.037874	0.037874	0.037874	0.037874	0.037874	0.037874
0.037874	0.037874	0.037874	0.037874	0.037874	0.037874	0.037874	0.037874	0.037874
0.037874	0.037874	0.037874	0.037874	0.037874	0.037874	0.037874	0.037874	0.037874
0	0	0	0	0	0	0	0	0
0.037874	0.037874	0.037874	0.037874	0.037874	0.037874	0.037874	0.037874	0.037874
0.037874	0.037874	0.037874	0.037874	0.037874	0.037874	0.037874	0.037874	0.037874
0	0	0	0	0	0	0	0	0
-0.25312	-0.25312	-0.25312	-9.92012	-0.25312	-0.25312	-0.25312	-0.25312	-0.25312
0	0	0	0	0	0	0	0	0
0	0	0	0	0	0	0	0	0
0	0	0	0	0	0	0	0	0
0	0	0	0	0	0	0	0	0
0	0	0	0	0	0	0	0	0
0	0	0	0	0	0	0	0	0
0	0	0	0	0	0	0	0	0
0	0	0	0	0	0	0	0	0
0	0	0	0	0	0	0	0	0
0	0	0	0	0	0	0	0	0
0	0	0	0	0	0	0	0	0
0	0	0	0	0	0	0	0	0
0	0	0	0	0	0	0	0	0
0.139516	0.139516	0.139516	0.139516	0.139516	0.139516	0.139516	0.139516	0.139516
12.87742	12.87742	12.87742	12.87742	12.87742	12.87742	12.87742	12.87742	12.87742
0.139516	0.139516	0.139516	0.139516	0.139516	0.139516	0.139516	0.139516	0.139516
0	0	0	0	0	0	0	0	0
0	0	0	0	0	0	0	0	0
0	0	0	0	0	0	0	0	0
0	0	0	0	0	0	0	0	0
0	0	0	0	0	0	0	0	0
0	0	0	0	0	0	0	0	0
0	0	0	0	0	0	0	0	0
0	0	0	0	0	0	0	0	0
0	0	0	0	0	0	0	0	0
0	0	0	0	0	0	0	0	0
0	0	0	0	0	0	0	0	0
0	0	0	0	0	0	0	0	0
0	0	0	0	0	0	0	0	0
0	0	0	0	0	0	0	0	0
0	0	0	0	0	0	0	0	0

0	0	0	0	0	0	0	0	0
0	0	0	0	0	0	0	0	0
0.571827	0.571827	0.571827	0.571827	0.571827	0.571827	0.571827	0.571827	9.985827
0.430967	0.430967	10.19797	10.09797	0.430967	10.42797	0.430967	10.40697	0.430967
0.430967	0.430967	10.19797	10.09797	0.430967	10.42797	0.430967	10.40697	0.430967
0.430967	0.430967	10.19797	10.09797	0.430967	10.42797	0.430967	10.40697	0.430967
0	0	0	0	0	0	0	0	0
0	0	0	0	0	0	0	0	0
0	0	0	0	0	0	0	0	0
12.73791	12.73791	12.73791	12.73791	12.73791	12.73791	12.73791	12.73791	12.73791
0	0	0	0	0	0	0	0	0
0	0	0	0	0	0	0	0	0
0	0	0	0	0	0	0	0	0
0.344598	0.344598	10.1116	0.344598	0.344598	0.344598	0.344598	0.344598	0.344598
1.22E-16	1.22E-16	4.57E-17	1.91E-16	1.94E-16	2.05E-16	1.22E-16	1.69E-16	1.15E-16
0.017378	0.017378	0.017378	0.017378	0.017378	0.017378	0.017378	0.017378	0.017378
0.004943	0.004943	0.004943	0.004943	0.004943	0.004943	0.004943	0.004943	0.004943
0.047343	0.047343	0.047343	0.047343	0.047343	0.047343	0.047343	0.047343	0.047343
0.085126	0.085126	0.085126	0.085126	0.085126	0.085126	0.085126	0.085126	0.085126
0.047713	0.047713	0.047713	0.047713	0.047713	0.047713	0.047713	0.047713	0.047713
0.089983	0.089983	0.089983	0.089983	0.089983	0.089983	0.089983	0.089983	0.089983
0.079878	0.079878	0.079878	0.079878	0.079878	0.079878	0.079878	0.079878	0.079878
0.042204	0.042204	0.042204	0.042204	0.042204	0.042204	0.042204	0.042204	0.042204
0.025	0.025	0.025	0.025	0.025	0.025	0.025	0.025	0.025
0.046124	0.046124	0.046124	0.046124	0.046124	0.046124	0.046124	0.046124	0.046124
0.047082	0.047082	0.047082	0.047082	0.047082	0.047082	0.047082	0.047082	0.047082
0.047147	0.047147	0.047147	0.047147	0.047147	0.047147	0.047147	0.047147	0.047147
0.028484	0.028484	0.028484	0.028484	0.028484	0.028484	0.028484	0.028484	0.028484
0.010279	0.010279	0.010279	0.010279	0.010279	0.010279	0.010279	0.010279	0.010279
0.108798	0.108798	0.108798	0.108798	0.108798	0.108798	0.108798	0.108798	0.108798
0.052047	0.052047	0.052047	0.052047	0.052047	0.052047	0.052047	0.052047	0.052047
0.04645	0.04645	0.04645	0.04645	0.04645	0.04645	0.04645	0.04645	0.04645
0.029682	0.029682	0.029682	0.029682	0.029682	0.029682	0.029682	0.029682	0.029682
0.016006	0.016006	0.016006	0.016006	0.016006	0.016006	0.016006	0.016006	0.016006
0.034669	0.034669	0.034669	0.034669	0.034669	0.034669	0.034669	0.034669	0.034669
0.000152	0.000152	0.000152	0.000152	0.000152	0.000152	0.000152	0.000152	0.000152
0	0	0	0	0	0	0	0	0
0.000305	0.000305	0.000305	0.000305	0.000305	0.000305	0.000305	0.000305	0.000305
0	0	0	0	0	0	0	0	0
0.000305	0.000305	0.000305	0.000305	0.000305	0.000305	0.000305	0.000305	0.000305
0.000305	0.000305	0.000305	0.000305	0.000305	0.000305	0.000305	0.000305	0.000305
0	0	0	0	0	0	0	0	0
0.000152	0.000152	0.000152	0.000152	0.000152	0.000152	0.000152	0.000152	0.000152
0	0	0	0	0	0	0	0	0
0	0	0	0	0	0	0	0	0
0	0	0	0	0	0	0	0	0
4.36E-05	4.36E-05	4.36E-05	4.36E-05	4.36E-05	4.36E-05	4.36E-05	4.36E-05	4.36E-05

0	0	0	0	0	0	0	0	0
2.18E-05	2.18E-05	2.18E-05	2.18E-05	2.18E-05	2.18E-05	2.18E-05	2.18E-05	2.18E-05
0	0	0	0	0	0	0	0	0
2.18E-05	2.18E-05	2.18E-05	2.18E-05	2.18E-05	2.18E-05	2.18E-05	2.18E-05	2.18E-05
0	0	0	0	0	0	0	0	0
2.18E-05	2.18E-05	2.18E-05	2.18E-05	2.18E-05	2.18E-05	2.18E-05	2.18E-05	2.18E-05
0	0	0	0	0	0	0	0	0
2.18E-05	2.18E-05	2.18E-05	2.18E-05	2.18E-05	2.18E-05	2.18E-05	2.18E-05	2.18E-05
0	0	0	0	0	0	0	0	0
2.18E-05	2.18E-05	2.18E-05	2.18E-05	2.18E-05	2.18E-05	2.18E-05	2.18E-05	2.18E-05
0	0	0	0	0	0	0	0	0
2.18E-05	2.18E-05	2.18E-05	2.18E-05	2.18E-05	2.18E-05	2.18E-05	2.18E-05	2.18E-05
0	0	0	0	0	0	0	0	0
0	0	0	0	0	0	0	0	0
0	0	0	0	0	0	0	0	0
2.18E-05	2.18E-05	2.18E-05	2.18E-05	2.18E-05	2.18E-05	2.18E-05	2.18E-05	2.18E-05
0	0	0	0	0	0	0	0	0
0	0	0	0	0	0	0	0	0
-227.568	-227.568	-413.141	-246.902	-227.568	-257.559	-227.568	-347.28	-302.88
227.5677	227.5677	413.1407	246.9017	227.5677	257.5587	227.5677	347.2797	302.8797
0	0	0	0	0	0	0	0	0
0	0	0	0	0	0	0	0	0
0	0	0	0	0	0	0	0	0
0	0	0	0	0	0	0	0	0
0	0	0	0	0	0	0	0	0
0	0	0	0	0	0	0	0	0
0	0	0	0	0	0	0	0	0
0.000005	0.000005	0	0.000005	0.000005	0	0.000005	0.000005	0.000005
0.000005	0.000005	0	0.000005	0.000005	0	0.000005	0.000005	0.000005
-5E-06	-5E-06	0	-5E-06	-5E-06	0	-5E-06	-5E-06	-5E-06
0.00001	0.00001	0	0.00001	0.00001	0	0.00001	0.00001	0.00001
3.33E-06	3.33E-06	0	3.33E-06	3.33E-06	0	3.33E-06	3.33E-06	3.33E-06
1.67E-06	1.67E-06	0	1.67E-06	1.67E-06	0	1.67E-06	1.67E-06	1.67E-06
1.67E-06	1.67E-06	0	1.67E-06	1.67E-06	0	1.67E-06	1.67E-06	1.67E-06
-1.7E-06	-1.7E-06	0	-1.7E-06	-1.7E-06	0	-1.7E-06	-1.7E-06	-1.7E-06
1.67E-06	1.67E-06	0	1.67E-06	1.67E-06	0	1.67E-06	1.67E-06	1.67E-06
0	0	0	0	0	0	0	0	0
0	0	0	0	0	0	0	0	0
0	0	0	0	0	0	0	0	0
0	0	0	0	0	0	0	0	0
0	0	0	0	0	0	0	0	0
0	0	0	0	0	0	0	0	0
0	0	0	0	0	0	0	0	0
0	0	0	0	0	0	0	0	0
0	0	0	0	0	0	0	0	0
0	0	0	0	0	0	0	0	0
0.037874	0.037874	0.037874	0.037874	0.037874	0.037874	0.037874	0.037874	0.037874

0	0	0	0	0	0	0	0	0

gly	pro	ser	tyr
**2.18E-05**	**2.18E-05**	**2.18E-05**	**2.18E-05**
0	0	0	0
-5	-5	-5	-5
-5	-5	-5	-5
0	0	0	0
0	0	0	0
-0.125	-0.125	-0.125	-0.125
0	0	0	0
0	0	0	0
0	0	0	0
0	0	0	0
0	0	0	0
0	0	0	0
0	0	0	0
0	0	0	0
-0.23	-0.23	-0.23	-0.23
0	0	0	0
0	0	0	0
0	0	0	0
0	0	0	0
0	0	0	0
0	0	0	0
0	0	0	0
0	0	0	0
-39.3733	-68.8693	-39.3733	-39.3733
36.40954	56.07354	36.40954	36.40954
292.4733	489.1133	292.4733	292.4733
15	15	15	15
-0.05216	-0.05216	-0.05216	-0.05216
-0.23	-0.23	-0.23	-0.23
0	0	0	0
0	0	0	0
0	0	0	0
0	0	0	0
0	0	0	0
0	0	0	0
5	5	5	5
0	0	0	0
0	0	0	0
0.125	0.125	0.125	0.125
0	0	0	0
0	0	0	0
0	0	0	0
0	0	0	0
0	0	0	0
0	0	0	0

0	0	0	0
0	0	0	0
0.23	0.23	0.23	0.23
0	0	0	0
0	0	0	0
0.23	0.23	0.23	0.23
0	0	0	0
0	0	0	0
0	0	0	0
0	0	0	0
5	5	5	5
4.999995	4.999995	4.999995	4.999995
4.999998	4.999998	4.999998	4.999998
0	0	0	0
4.999998	4.999998	4.999998	4.999998
-5	-5	-5	-5
9.999998	9.999998	9.999998	9.999998
9.999998	9.999998	9.999998	9.999998
9.569031	-0.26297	9.569031	9.569031
9.569031	-0.26297	9.569031	9.569031
9.746876	9.746876	9.746876	9.746876
0	0	0	0
0	0	0	0
0.177845	10.00984	0.177845	0.177845
0	0	0	0
10.43098	20.26298	10.43098	10.43098
10.60882	30.27282	10.60882	10.60882
0	0	0	0
10.43098	20.26298	10.43098	10.43098
11.22473	30.88873	11.22473	11.22473
9.999998	9.999998	9.999998	9.999998
11.22473	30.88873	11.22473	11.22473
0	0	0	0
9.999998	9.999998	9.999998	9.999998
12.70003	12.70003	12.70003	12.70003
12.70003	12.70003	12.70003	12.70003
0	0	0	0
12.70003	12.70003	12.70003	12.70003
13.2887	23.1207	13.2887	13.2887
13.45591	23.28791	13.45591	13.45591
13.49379	23.32579	13.49379	13.49379
13.49379	23.32579	13.49379	13.49379
23.92476	43.58876	23.92476	23.92476
49.89181	89.21981	49.89181	49.89181
28.85488	48.51888	28.85488	28.85488
78.74669	137.7387	78.74669	78.74669
39.37335	68.86935	39.37335	39.37335

207.3852	364.6972	207.3852	207.3852
1.603164	1.603164	1.603164	1.603164
0.125	0.125	0.125	0.125
0.125	0.125	0.125	0.125
0.125	0.125	0.125	0.125
0.125	0.125	0.125	0.125
0	0	0	0
0	0	0	0
0	0	0	0
0	0	0	0
0.125	0.125	0.125	0.125
0.125	0.125	0.125	0.125
0.125	0.125	0.125	0.125
0.125	0.125	0.125	0.125
0	0	0	0
0	0	0	0
0	0	0	0
0	0	0	0
0.355	0.355	0.355	0.355
0.355	0.355	0.355	0.355
0.355	0.355	0.355	0.355
0.355	0.355	0.355	0.355
0.355	0.355	0.355	0.355
0.355	0.355	0.355	0.355
0.355	0.355	0.355	0.355
0.355	0.355	0.355	0.355
0.355	0.355	0.355	0.355
0.355	0.355	0.355	0.355
0.355	0.355	0.355	0.355
0.355	0.355	0.355	0.355
0.355	0.355	0.355	0.355
0.355	0.355	0.355	0.355
0.355	0.355	0.355	0.355
0.355	0.355	0.355	0.355
0.355	0.355	0.355	0.355
0.355	0.355	0.355	0.355
0.355	0.355	0.355	0.355
0.355	0.355	0.355	0.355
0.355	0.355	0.355	0.355
0.355	0.355	0.355	0.355
0.355	0.355	0.355	0.355
0.392874	0.392874	0.392874	0.392874
0	0	0	0
0	0	0	0
0	0	0	0
0	0	0	0
0	0	0	0

0	0	0	0
0	0	0	0
0	0	0	0
0	0	0	0
0	0	0	0
0	0	0	0
0	0	0	0
0	0	0	0
0	0	0	0
0.23	0.23	0.23	0.23
0	0	0	0
0	0	0	0
0	0	0	0
0	0	0	0
0	0	0	0
0	0	0	0
0	0	0	0
0	0	0	0
0	0	0	0
0.23	0.23	0.23	0.23
0	0	0	0
0	0	0	0
0	0	0	0
0	0	0	0
0	0	0	0
0	0	0	0
0	0	0	0
0	0	0	0
0	0	0	0
0.23	0.23	0.23	0.23
0	0	0	0
0	0	0	0
0	0	0	0
0	0	0	0
0	0	0	0
0	0	0	0
0	0	0	0
0	0	0	0
0	0	0	0
0.23	0.23	0.23	0.23
0	0	0	0
0	0	0	0
0	0	0	0
0	0	0	0
0	0	0	0
0	0	0	0
0	0	0	0

0	0	0	0
0	0	0	0
0	0	0	0
0	0	0	0
0	0	0	0
0	0	0	0
0	0	0	0
0	0	0	0
0	0	0	0
-0.04645	-0.04645	-0.04645	-0.04645
-0.01601	-0.01601	-0.01601	-0.01601
-0.062	-0.062	-0.062	-0.062
-0.123	-0.123	-0.123	-0.123
-0.08998	-0.08998	-0.08998	-0.08998
-0.025	-0.025	-0.025	-0.025
-0.02968	-0.02968	-0.02968	-0.02968
-0.04734	-0.04734	-0.04734	-0.04734
-0.00494	-0.00494	-0.00494	-0.00494
-0.233	-0.233	-0.233	-0.233
-0.333	-0.333	-0.333	-0.333
0.537158	0.537158	0.537158	0.537158
-0.003	-0.003	-0.003	-0.003
-0.01028	-0.01028	-0.01028	-0.01028
-0.024	-0.024	-0.024	-0.024
-0.586	-0.586	-0.586	-0.586
-0.04715	-0.04715	-0.04715	-0.04715
-0.168	-10	-0.168	-0.168
0.384843	10.21684	0.384843	0.384843
-0.01738	-0.01738	-0.01738	-0.01738
0.242956	0.242956	0.242956	0.242956
0.190796	0.190796	0.190796	0.190796
0.190796	0.190796	0.190796	0.190796
0.190796	0.190796	0.190796	0.190796
0.190796	0.190796	0.190796	0.190796
0.190796	0.190796	0.190796	0.190796
0.190796	0.190796	0.190796	0.190796
0.190796	0.190796	0.190796	0.190796
0.190796	0.190796	0.190796	0.190796
0.190796	0.190796	0.190796	0.190796
0.205083	0.205083	0.205083	0.205083
0.205083	0.205083	0.205083	0.205083
0.205083	0.205083	0.205083	0.205083
0.014287	0.014287	0.014287	0.014287
0.014287	0.014287	0.014287	0.014287
0.014287	0.014287	0.014287	0.014287
0.014287	0.014287	0.014287	0.014287
0.014287	0.014287	0.014287	0.014287

0.014287	0.014287	0.014287	0.014287
0.014287	0.014287	0.014287	0.014287
0.037874	0.037874	0.037874	0.037874
0.037874	0.037874	0.037874	0.037874
0.037874	0.037874	0.037874	0.037874
0.037874	0.037874	0.037874	0.037874
0.037874	0.037874	0.037874	0.037874
0.037874	0.037874	0.037874	0.037874
0.037874	0.037874	0.037874	0.037874
0.037874	0.037874	0.037874	0.037874
0.037874	0.037874	0.037874	0.037874
0	0	0	0
0.037874	0.037874	0.037874	0.037874
0.037874	0.037874	0.037874	0.037874
0	0	0	0
-0.25312	-0.25312	-0.25312	-0.25312
0	0	0	0
0	0	0	0
0	0	0	0
0	0	0	0
0	0	0	0
0	0	0	0
0	0	0	0
0	0	0	0
0	0	0	0
0	0	0	0
0	0	0	0
0	0	0	0
0	0	0	0
0.139516	9.971516	0.139516	0.139516
12.87742	22.70942	12.87742	12.87742
0.139516	9.971516	0.139516	0.139516
0	0	0	0
0	0	0	0
0	0	0	0
0	0	0	0
0	0	0	0
0	0	0	0
0	0	0	0
0	0	0	0
0	0	0	0
0	0	0	0
0	0	0	0
0	0	0	0
0	0	0	0
0	0	0	0
0	0	0	0

0	0	0	0
0	0	0	0
0.571827	0.571827	0.571827	0.571827
0.430967	10.26297	0.430967	0.430967
0.430967	10.26297	0.430967	0.430967
0.430967	10.26297	0.430967	0.430967
0	0	0	0
0	0	0	0
0	0	0	0
12.73791	12.73791	12.73791	12.73791
0	0	0	0
0	0	0	0
0	0	0	0
0.344598	10.1766	0.344598	0.344598
1.22E-16	9E-17	1.22E-16	1.22E-16
0.017378	0.017378	0.017378	0.017378
0.004943	0.004943	0.004943	0.004943
0.047343	0.047343	0.047343	0.047343
0.085126	0.085126	0.085126	0.085126
0.047713	0.047713	0.047713	0.047713
0.089983	0.089983	0.089983	0.089983
0.079878	0.079878	0.079878	0.079878
0.042204	0.042204	0.042204	0.042204
0.025	0.025	0.025	0.025
0.046124	0.046124	0.046124	0.046124
0.047082	0.047082	0.047082	0.047082
0.047147	0.047147	0.047147	0.047147
0.028484	0.028484	0.028484	0.028484
0.010279	0.010279	0.010279	0.010279
0.108798	0.108798	0.108798	0.108798
0.052047	0.052047	0.052047	0.052047
0.04645	0.04645	0.04645	0.04645
0.029682	0.029682	0.029682	0.029682
0.016006	0.016006	0.016006	0.016006
0.034669	0.034669	0.034669	0.034669
0.000152	0.000152	0.000152	0.000152
0	0	0	0
0.000305	0.000305	0.000305	0.000305
0	0	0	0
0.000305	0.000305	0.000305	0.000305
0.000305	0.000305	0.000305	0.000305
0	0	0	0
0.000152	0.000152	0.000152	0.000152
0	0	0	0
0	0	0	0
0	0	0	0
4.36E-05	4.36E-05	4.36E-05	4.36E-05

0	0	0	0
2.18E-05	2.18E-05	2.18E-05	2.18E-05
0	0	0	0
2.18E-05	2.18E-05	2.18E-05	2.18E-05
0	0	0	0
2.18E-05	2.18E-05	2.18E-05	2.18E-05
0	0	0	0
2.18E-05	2.18E-05	2.18E-05	2.18E-05
0	0	0	0
2.18E-05	2.18E-05	2.18E-05	2.18E-05
0	0	0	0
2.18E-05	2.18E-05	2.18E-05	2.18E-05
0	0	0	0
0	0	0	0
0	0	0	0
2.18E-05	2.18E-05	2.18E-05	2.18E-05
0	0	0	0
0	0	0	0
-227.568	-394.712	-227.568	-227.568
227.5677	394.7117	227.5677	227.5677
0	0	0	0
0	0	0	0
0	0	0	0
0	0	0	0
0	0	0	0
0	0	0	0
0	0	0	0
0.000005	0.000005	0.000005	0.000005
0.000005	0.000005	0.000005	0.000005
-5E-06	-5E-06	-5E-06	-5E-06
0.00001	0.00001	0.00001	0.00001
3.33E-06	3.33E-06	3.33E-06	3.33E-06
1.67E-06	1.67E-06	1.67E-06	1.67E-06
1.67E-06	1.67E-06	1.67E-06	1.67E-06
-1.7E-06	-1.7E-06	-1.7E-06	-1.7E-06
1.67E-06	1.67E-06	1.67E-06	1.67E-06
0	0	0	0
0	0	0	0
0	0	0	0
0	0	0	0
0	0	0	0
0	0	0	0
0	0	0	0
0	0	0	0
0	0	0	0
0	0	0	0
0.037874	0.037874	0.037874	0.037874

0	0	0	0

Flux	**control**	**arg**	**his**	**ile**	**leu**	**lys**	**met**	**phe**
**Prot Synth**	**2.18E-05**	**2.18E-05**	**2.18E-05**	**2.18E-05**	**2.18E-05**	**2.18E-05**	**2.83E-05**	**2.18E-05**
Obj_atp	0	0	0	0	0	0	0	0
Ex_ins	-5	-5	-5	-5	-5	-5	1.88E-18	-5
Ex_gluc	-5	-5	-5	-5	-5	-5	1.88E-18	-5
Ex_lac	0	0	0	0	0	0	0	0
Ex_pyr	0	0	0	0.00001	0	0	0	0
Ex_pal	-0.125	-0.125	-0.125	0	-0.125	-0.125	0	-0.125
Ex_prop	0	0	0	0	0	0	0	0
Ex_but	0	0	0	0	0	0	0	0
Ex_5c	0	0	0	0	0	0	0	0
Ex_6c	0	0	0	0	0	0	0	0
Ex_8c	0	0	0	0	0	0	0	0
Ex_9c	0	0	0	0	0	0	0	0
Ex_10c	0	0	0	0	0	0	0	0
Ex_12c	0	0	0	0	0	0	0	0
Ex_14c	-0.23	-0.23	-0.23	0	-0.23	-0.23	0	-0.23
Ex_18c	0	0	0	0	0	0	0	0
Ex_glyc	0	0	0	0	0	0	0	0
Ex_tag	0	0	0	0	0	0	0	0
Ex_dag	0	0	0	0	0	0	0	0
Ex_mag	0	0	0	0	0	0	0	0
Ex_akg	0	0	0	9.99999	0	0	0	0
Ex_oaa	0	0	0	0	0	0	0	0
Ex_rib	0	0	0	0	0	0	0	0
Nut_o2	-39.3733	-39.3733	-39.8624	-179999	-40.2626	-39.3733	-0.95554	-39.3733
Nut_co2	36.40954	36.40954	36.67631	159987.4	37.12092	36.40954	0.352055	36.40954
Nut_h2o	292.4733	292.4733	295.4077	999999	298.1643	292.4733	5.186478	-999697
Nut_p	15	15	15	15	15	15	9.38E-16	15
Nut_hco3	-0.05216	-0.05216	-0.05216	-40001.7	-0.23001	-0.05216	-0.01238	-0.05216
Nut_coa	-0.23	-0.23	-0.23	0	-0.23	-0.23	0	-0.23
Nut_carn	0	0	0	0	0	0	0	0
Nut_dhf	0	0	0	0	0	0	0	0
LPL	0	0	0	0	0	0	0	0
DAGL	0	0	0	0	0	0	0	0
12DAG	0	0	0	0	0	0	0	0
MAGL	0	0	0	0	0	0	0	0
GLUT4	5	5	5	5	5	5	-1.9E-18	5
MCT4	0	0	0	0	0	0	0	0
MCT_pyr	0	0	0	-0.00001	0	0	0	0
FAT/CD36	0.125	0.125	0.125	0	0.125	0.125	0	0.125
FATprop	0	0	0	0	0	0	0	0
FATbut	0	0	0	0	0	0	0	0
FAT5c	0	0	0	0	0	0	0	0
FAT6c	0	0	0	0	0	0	0	0
FAT8c	0	0	0	0	0	0	0	0
FAT9c	0	0	0	0	0	0	0	0

FAT10c	0	0	0	0	0	0	0	0
FAT12c	0	0	0	0	0	0	0	0
FAT14c	0.23	0.23	0.23	0	0.23	0.23	0	0.23
FAT18c	0	0	0	0	0	0	0	0
Alc_glyc	0	0	0	0	0	0	0	0
COA	0.23	0.23	0.23	0	0.23	0.23	0	0.23
SLC22A5/O	0	0	0	0	0	0	0	0
Xport_rib	0	0	0	0	0	0	0	0
Dr_akg	0	0	0	9.99999	0	0	0	0
Dr_oaa	0	0	0	0	0	0	0	0
Hex	5	5	5	5	5	5	-1.9E-18	5
PGI	4.999995	4.999995	5	5	4.999995	4.999995	-1.9E-18	4.999995
PFK	4.999998	4.999998	5	5	4.999998	4.999998	-1.9E-18	4.999998
FBP	0	0	0	0	0	0	0	0
Ald	4.999998	4.999998	5	5	4.999998	4.999998	-1.9E-18	4.999998
TPI	-5	-5	-5	-5	-5	-5	1.88E-18	-5
D3PDeh	9.999998	9.999998	10	10	9.999998	9.999998	-3.8E-18	9.999998
PGK	9.999998	9.999998	10	10	9.999998	9.999998	-3.8E-18	9.999998
PGM	9.569031	9.569031	9.746878	-39992.2	9.746876	9.569031	-0.2292	9.569031
Eno	9.569031	9.569031	9.746878	-39992.2	9.746876	9.569031	-0.2292	9.569031
PyrK	9.746876	9.746876	9.746878	279994.6	9.746876	9.746876	0	999999
PEPSynth	0	0	0	279994.9	0	0	0.229204	999989.3
PEPCarbK	0	0	0	0	0	0	0	0
PEPCarbl	0.177845	0.177845	0	39991.94	0	0.177845	0	0.177845
LacDeh	0	0	0	0	0	0	0	0
MalDehC(n	10.43098	10.43098	10.25312	40012.19	10.25313	10.43098	0.199158	10.43098
ASTC	10.60882	10.60882	10.25312	80004.14	10.25313	10.60882	0.199158	10.60882
G3PDehC	0	0	0	0	0	0	0	0
Mal-AKGEX	10.43098	10.43098	10.25312	40012.19	10.25313	10.43098	0.199158	10.43098
Glu-AspEx	11.22473	11.22473	11.04688	80004.17	11.04688	11.22473	0.261387	11.22473
MCT	9.999998	9.999998	10	0	9.999998	9.999998	0	9.999998
ASTM	11.22473	11.22473	11.04688	80004.17	11.04688	11.22473	0.261387	11.22473
G3PDehM	0	0	0	0	0	0	0	0
PyrSynth/P	9.999998	9.999998	10	0	9.999998	9.999998	0	9.999998
CitSynth	12.70003	12.70003	12.87788	40001.74	13.05572	12.70003	0.11593	12.70003
Acon	12.70003	12.70003	12.87788	40001.74	13.05572	12.70003	0.11593	12.70003
IsoDeh	0	0	0	40001.74	0	0	0	0
IsoDehnad	12.70003	12.70003	12.87788	0	13.05572	12.70003	0.11593	12.70003
AKGDeh	13.2887	13.2887	13.46655	39991.79	13.64439	13.2887	0	13.2887
SunCoaSyn	13.45591	13.45591	13.63376	79993.72	13.63376	13.45591	0.165775	13.45591
SucDeh(I)	13.49379	13.49379	13.67163	79993.72	13.84948	13.49379	0.178159	13.49379
Fum	13.49379	13.49379	13.67163	79993.72	13.84948	13.49379	0.178159	13.49379
MalDehM	23.92476	23.92476	23.92475	120005.9	24.10261	23.92476	0.377317	23.92476
NADHDeh(	49.89181	49.89181	50.2475	280003.6	50.60319	49.89181	1.146327	49.89181
ETFUO	28.85488	28.85488	29.47734	79994.05	29.92195	28.85488	0.764746	28.85488
UCytC(III)	78.74669	78.74669	79.72484	359997.7	80.52514	78.74669	1.911073	78.74669
CytCO(IV)	39.37335	39.37335	39.86242	179998.8	40.26257	39.37335	0.955537	39.37335

ATPSynth(\	207.3852	207.3852	209.6972	999999	211.6535	207.3852	4.968474	207.3852
AMP_ADPs	1.603164	1.603164	1.781009	299990.7	1.781009	1.603164	1.410971	999990.9
PalTK	0.125	0.125	0.125	0	0.125	0.125	0	0.125
CPT1B/CHK	0.125	0.125	0.125	0	0.125	0.125	0	0.125
SLC25A20/	0.125	0.125	0.125	0	0.125	0.125	0	0.125
CPT2	0.125	0.125	0.125	0	0.125	0.125	0	0.125
CRAT(mit)	0	0	0	0	0	0	0	0
CRAT Trans	0	0	0	0	0	0	0	0
CRAT(cyt)	0	0	0	0	0	0	0	0
Acetyl-coaL	0	0	0	0	0	0	0	0
AcylCoADe	0.125	0.125	0.125	0	0.125	0.125	0	0.125
EnoCoADel	0.125	0.125	0.125	0	0.125	0.125	0	0.125
3HA-CoADe	0.125	0.125	0.125	0	0.125	0.125	0	0.125
AcylCoAAT	0.125	0.125	0.125	0	0.125	0.125	0	0.125
AcylCoADe	0	0	0	0	0	0	0	0
EnoCoADel	0	0	0	0	0	0	0	0
3HA-CoADe	0	0	0	0	0	0	0	0
AcylCoAAT	0	0	0	0	0	0	0	0
AcylCoADe	0.355	0.355	0.355	0	0.355	0.355	0	0.355
EnoCoADel	0.355	0.355	0.355	0	0.355	0.355	0	0.355
3HA-CoADe	0.355	0.355	0.355	0	0.355	0.355	0	0.355
AcylCoAAT	0.355	0.355	0.355	0	0.355	0.355	0	0.355
AcylCoADe	0.355	0.355	0.355	0	0.355	0.355	0	0.355
EnoCoADel	0.355	0.355	0.355	0	0.355	0.355	0	0.355
3HA-CoADe	0.355	0.355	0.355	0	0.355	0.355	0	0.355
AcylCoAAT	0.355	0.355	0.355	0	0.355	0.355	0	0.355
AcylCoADe	0.355	0.355	0.355	0	0.355	0.355	0	0.355
EnoCoADel	0.355	0.355	0.355	0	0.355	0.355	0	0.355
3HA-CoADe	0.355	0.355	0.355	0	0.355	0.355	0	0.355
AcylCoAAT	0.355	0.355	0.355	0	0.355	0.355	0	0.355
AcylCoADe	0.355	0.355	0.355	0	0.355	0.355	0	0.355
EnoCoADel	0.355	0.355	0.355	0	0.355	0.355	0	0.355
3HA-CoADe	0.355	0.355	0.355	0	0.355	0.355	0	0.355
AcylCoAAT	0.355	0.355	0.355	0	0.355	0.355	0	0.355
AcylCoADe	0.355	0.355	0.355	0	0.355	0.355	0	0.355
EnoCoADel	0.355	0.355	0.355	0	0.355	0.355	0	0.355
3HA-CoADe	0.355	0.355	0.355	0	0.355	0.355	0	0.355
AcylCoAAT	0.355	0.355	0.355	0	0.355	0.355	0	0.355
ButyrlCoAD	0.355	0.355	0.355	0	0.355	0.355	0	0.355
EnoCoADel	0.355	0.355	0.443922	0	0.355	0.355	0.04558	0.355
3HA-CoADe	0.355	0.355	0.443922	0	0.355	0.355	0.04558	0.355
AcylCoAAT	0.392874	0.392874	0.481796	0	0.570719	0.392874	0.057965	0.392874
GK	0	0	0	0	0	0	0	0
GPAT4	0	0	0	0	0	0	0	0
ABHD5	0	0	0	0	0	0	0	0
DAGZ/DAG	0	0	0	0	0	0	0	0
DGAT1	0	0	0	0	0	0	0	0

St_TAG	0	0	0	0	0	0	0	0
ACAS2	0	0	0	0	0	0	0	0
ACSM-But	0	0	0	0	0	0	0	0
ASCM-5c	0	0	0	0	0	0	0	0
ASCM-6c	0	0	0	0	0	0	0	0
ACSM-8c	0	0	0	0	0	0	0	0
ACSM-9c	0	0	0	0	0	0	0	0
ACSM-10c	0	0	0	0	0	0	0	0
ACSM-12c	0	0	0	0	0	0	0	0
ACSM-14c	0.23	0.23	0.23	0	0.23	0.23	0	0.23
ACSM-18c	0	0	0	0	0	0	0	0
CPT1B/CHK	0	0	0	0	0	0	0	0
CPT1B/CHK	0	0	0	0	0	0	0	0
CPT1B/CHK	0	0	0	0	0	0	0	0
CPT1B/CHK	0	0	0	0	0	0	0	0
CPT1B/CHK	0	0	0	0	0	0	0	0
CPT1B/CHK	0	0	0	0	0	0	0	0
CPT1B/CHK	0	0	0	0	0	0	0	0
CPT1B/CHK	0	0	0	0	0	0	0	0
CPT1B/CHK	0.23	0.23	0.23	0	0.23	0.23	0	0.23
CPT1B/CHK	0	0	0	0	0	0	0	0
SLC25A20/	0	0	0	0	0	0	0	0
SLC25A20/	0	0	0	0	0	0	0	0
SLC25A20/	0	0	0	0	0	0	0	0
SLC25A20/	0	0	0	0	0	0	0	0
SLC25A20/	0	0	0	0	0	0	0	0
SLC25A20/	0	0	0	0	0	0	0	0
SLC25A20/	0	0	0	0	0	0	0	0
SLC25A20/	0	0	0	0	0	0	0	0
SLC25A20/	0.23	0.23	0.23	0	0.23	0.23	0	0.23
SLC25A20/	0	0	0	0	0	0	0	0
CPT2	0	0	0	0	0	0	0	0
CPT2	0	0	0	0	0	0	0	0
CPT2	0	0	0	0	0	0	0	0
CPT2	0	0	0	0	0	0	0	0
CPT2	0	0	0	0	0	0	0	0
CPT2	0	0	0	0	0	0	0	0
CPT2	0	0	0	0	0	0	0	0
CPT2	0	0	0	0	0	0	0	0
CPT2	0.23	0.23	0.23	0	0.23	0.23	0	0.23
CPT2	0	0	0	0	0	0	0	0
AcylCoADe	0	0	0	0	0	0	0	0
EnoCoADel	0	0	0	0	0	0	0	0
3HA-CoADe	0	0	0	0	0	0	0	0
AcylCoAAT	0	0	0	0	0	0	0	0
AcylCoADe	0	0	0	0	0	0	0	0
EnoCoADel	0	0	0	0	0	0	0	0

3HA-CoADe	0	0	0	0	0	0	0	0
AcylCoAAT-	0	0	0	0	0	0	0	0
AcylCoADe	0	0	0	0	0	0	0	0
EnoCoADel	0	0	0	0	0	0	0	0
3HA-CoADe	0	0	0	0	0	0	0	0
AcylCoAAT-	0	0	0	0	0	0	0	0
AKR1A1	0	0	0	0	0	0	0	0
ALDH3A2	0	0	0	0	0	0	0	0
GLYCTK	0	0	0	0	0	0	0	0
EAA_arg	-0.04645	-0.04645	-0.04645	-0.04645	-0.04645	-0.04645	-0.06036	-0.04645
EAA_his	-0.01601	-0.01601	-0.01601	-0.01601	-0.01601	-0.01601	-0.05085	-0.01601
EAA_ile	-0.062	-0.062	-0.062	-40001.8	-0.062	-0.062	-0.062	-0.062
EAA_leu	-0.123	-0.123	-0.123	-0.08513	-0.30084	-0.123	-0.123	-0.123
EAA_lys	-0.08998	-0.08998	-0.17891	-0.08998	-0.08998	-0.08998	-0.16251	-0.08998
EAA_met	-0.025	-0.025	-0.025	-0.025	-0.025	-0.025	-0.03249	-0.025
EAA_phe	-0.02968	-0.02968	-0.02968	-0.02968	-0.02968	-0.02968	-0.03857	-0.02968
EAA_thr	-0.04734	-0.04734	-0.04734	-0.04734	-0.04734	-0.04734	-0.06152	-0.04734
EAA_trp	-0.00494	-0.00494	-0.00494	-0.00494	-0.00494	-0.00494	-0.00642	-0.00494
EAA_val	-0.233	-0.233	-0.233	-0.233	-0.233	-0.233	-0.233	-0.233
NEAA_ala	-0.333	-0.333	-0.333	-0.333	-0.333	-0.333	-0.333	-0.333
NEAA_asn	0.537158	0.537158	0.715003	-0.041	0.715003	0.537158	-0.041	0.537158
NEAA_asp	-0.003	-0.003	-0.003	-0.003	-0.003	-0.003	-0.003	-0.003
NEAA_cys	-0.01028	-0.01028	-0.01028	-0.01028	-0.01028	-0.01028	-0.01336	-0.01028
NEAA_glu	-0.024	-0.024	-0.024	-0.024	-0.024	-0.024	-0.024	-0.024
NEAA_gln	-0.586	-0.586	-0.586	-0.586	-0.586	-0.586	-0.586	-0.586
NEAA_gly	-0.04715	-0.04715	-0.04715	-0.04715	-0.04715	-0.04715	-0.09131	-0.04715
NEAA_pro	-0.168	-0.168	-0.168	-0.168	-0.168	-0.168	-0.168	-0.168
NEAA_ser	0.384843	0.384843	0.206998	40002.15	0.206998	0.384843	0.199317	0.384843
NEAA_tyr	-0.01738	-0.01738	-0.01738	-0.01738	-0.01738	-0.01738	-0.02258	-0.01738
DLDH	0.242956	0.242956	0.242956	40001.93	0.420801	0.242956	0.190544	0.242956
MT_val	0.190796	0.190796	0.190796	0.190796	0.190796	0.190796	0.178159	0.190796
BCAT2-val	0.190796	0.190796	0.190796	0.190796	0.190796	0.190796	0.178159	0.190796
BCKDHB-va	0.190796	0.190796	0.190796	0.190796	0.190796	0.190796	0.178159	0.190796
BCKADE2-v	0.190796	0.190796	0.190796	0.190796	0.190796	0.190796	0.178159	0.190796
ACADSB	0.190796	0.190796	0.190796	0.190796	0.190796	0.190796	0.178159	0.190796
ECHS1	0.190796	0.190796	0.190796	0.190796	0.190796	0.190796	0.178159	0.190796
HIBCH	0.190796	0.190796	0.190796	0.190796	0.190796	0.190796	0.178159	0.190796
HIBADH	0.190796	0.190796	0.190796	0.190796	0.190796	0.190796	0.178159	0.190796
ALDH6A1	0.190796	0.190796	0.190796	0.190796	0.190796	0.190796	0.178159	0.190796
PCCB	0.205083	0.205083	0.205083	40001.93	0.205083	0.205083	0.178159	0.205083
MCEE	0.205083	0.205083	0.205083	40001.93	0.205083	0.205083	0.178159	0.205083
MUT	0.205083	0.205083	0.205083	40001.93	0.205083	0.205083	0.178159	0.205083
MT_ile	0.014287	0.014287	0.014287	40001.74	0.014287	0.014287	0	0.014287
BCAT-ile	0.014287	0.014287	0.014287	40001.74	0.014287	0.014287	0	0.014287
BCKDHB-ile	0.014287	0.014287	0.014287	40001.74	0.014287	0.014287	0	0.014287
BCKADE2-il	0.014287	0.014287	0.014287	40001.74	0.014287	0.014287	0	0.014287
ECHS1	0.014287	0.014287	0.014287	40001.74	0.014287	0.014287	0	0.014287

HSD17B10	0.014287	0.014287	0.014287	40001.74	0.014287	0.014287	0	0.014287
ACAT1	0.014287	0.014287	0.014287	40001.74	0.014287	0.014287	0	0.014287
MT_leu	0.037874	0.037874	0.037874	-1.5E-13	0.215719	0.037874	0.012385	0.037874
BCAT2-leu	0.037874	0.037874	0.037874	-1.5E-13	0.215719	0.037874	0.012385	0.037874
BCKDHB-le	0.037874	0.037874	0.037874	-1.5E-13	0.215719	0.037874	0.012385	0.037874
BCKADE2-l	0.037874	0.037874	0.037874	0	0.215719	0.037874	0.012385	0.037874
IVD	0.037874	0.037874	0.037874	0	0.215719	0.037874	0.012385	0.037874
MCC1,MCC	0.037874	0.037874	0.037874	0	0.215719	0.037874	0.012385	0.037874
MGCA	0.037874	0.037874	0.037874	0	0.215719	0.037874	0.012385	0.037874
HMGCL	0.037874	0.037874	0.037874	0	0.215719	0.037874	0.012385	0.037874
OXCT	0.037874	0.037874	0.037874	0	0.215719	0.037874	0.012385	0.037874
HMGCS2	0	0	0	0	0	0	0	0
GluDeh	0.037874	0.037874	0.215719	-8.3E-12	0.215719	0.037874	0.103545	0.037874
GlnSynth	0.037874	0.037874	0.215719	19994.86	0.215719	0.037874	0.163639	0.037874
GLN_Elim	0	0	0	0.540284	0	0	0.677958	0
ALT	-0.25312	-0.25312	-0.25312	-0.25312	-0.25312	-0.25312	-0.2292	-0.25312
ALA_Elim	0	0	0	0	0	0	0	0
SDS	0	0	0	0	0	0	0	0
CBS	0	0	0	0	0	0	0	0
CTH	0	0	0	0	0	0	0	0
ILVBL	0	0	0	0	0	0	0	0
2a2hButLe	0	0	0	0	0	0	0	0
MT_lys	0	0	0.088922	0	0	0	0.04558	0
AASS	0	0	0.088922	0	0	0	0.04558	0
AASS	0	0	0.088922	0	0	0	0.04558	0
aAASADeh	0	0	0.088922	0	0	0	0.04558	0
KATII	0	0	0.088922	0	0	0	0.04558	0
lysdeg	0	0	0.088922	0	0	0	0.04558	0
GCDH	0	0	0.088922	0	0	0	0.04558	0
MT_pro	0.139516	0.139516	0.139516	0.139516	0.139516	0.139516	0.130987	0.139516
PRODH	12.87742	12.87742	13.23311	0.139516	13.41096	12.87742	0.350462	12.87742
ALDH4a1	0.139516	0.139516	0.139516	0.139516	0.139516	0.139516	0.130987	0.139516
GATM	0	0	0	0	0	0	0	0
Dr_orn	0	0	0	0	0	0	0	0
GAMT	0	0	0	0	0	0	0	0
Dr_cr	0	0	0	0	0	0	0	0
AdoHcyase	0	0	0	0	0	0	0	0
MTR	0	0	0	0	0	0	0	0
AdoMet	0	0	0	0	0	0	0	0
SHMT	0	0	0	0	0	0	-0.03005	0
MTHFR	0	0	0	0	0	0	0	0
DHFR	0	0	0	0	0	0	0	0
HAL	0	0	0	0	0	0	0.030047	0
FLJ31300	0	0	0	0	0	0	0.030047	0
MGC35366	0	0	0	0	0	0	0.030047	0
FTCD1	0	0	0	0	0	0	0.030047	0
FTCD2	0	0	0	0	0	0	0.030047	0

FT	0	0	0	0	0	0	0.030047	0
ASNase	0	0	0	19994.86	0	0	0	0
ASNSynth	0.571827	0.571827	0.749672	19994.86	0.749672	0.571827	0.00405	0.571827
PHGDH	0.430967	0.430967	0.253122	40002.19	0.253122	0.430967	0.229204	0.430967
PSA	0.430967	0.430967	0.253122	40002.19	0.253122	0.430967	0.229204	0.430967
PSPH	0.430967	0.430967	0.253122	40002.19	0.253122	0.430967	0.229204	0.430967
P5CS	0	0	0	0	0	0	0	0
PYCS	0	0	0	0	0	0	0	0
Spont	0	0	0	0	0	0	0	0
PYRCR1	12.73791	12.73791	13.0936	-8.3E-12	13.27144	12.73791	0.219476	12.73791
PAG	0	0	0	0	0	0	0	0
PCBD	0	0	0	0	0	0	0	0
QDPR	0	0	0	0	0	0	0	0
MT_glu	0.344598	0.344598	0.344598	40002.07	0.344598	0.344598	0.309146	0.344598
tRNA	1.22E-16	1.05E-16	-2.7E-16	-2.1E-16	-1.6E-16	2.1E-16	-1.5E-16	-3.4E-17
YARS	0.017378	0.017378	0.017378	0.017378	0.017378	0.017378	0.022581	0.017378
WARS	0.004943	0.004943	0.004943	0.004943	0.004943	0.004943	0.006424	0.004943
TARS	0.047343	0.047343	0.047343	0.047343	0.047343	0.047343	0.061519	0.047343
LARS	0.085126	0.085126	0.085126	0.085126	0.085126	0.085126	0.110615	0.085126
IARS	0.047713	0.047713	0.047713	0.047713	0.047713	0.047713	0.062	0.047713
KARS	0.089983	0.089983	0.089983	0.089983	0.089983	0.089983	0.116926	0.089983
AARS	0.079878	0.079878	0.079878	0.079878	0.079878	0.079878	0.103796	0.079878
VARS	0.042204	0.042204	0.042204	0.042204	0.042204	0.042204	0.054841	0.042204
MARS	0.025	0.025	0.025	0.025	0.025	0.025	0.032486	0.025
SARS	0.046124	0.046124	0.046124	0.046124	0.046124	0.046124	0.059934	0.046124
DARS	0.047082	0.047082	0.047082	0.047082	0.047082	0.047082	0.061179	0.047082
GARS	0.047147	0.047147	0.047147	0.047147	0.047147	0.047147	0.061264	0.047147
PARS	0.028484	0.028484	0.028484	0.028484	0.028484	0.028484	0.037013	0.028484
CARS	0.010279	0.010279	0.010279	0.010279	0.010279	0.010279	0.013356	0.010279
EPRS	0.108798	0.108798	0.108798	0.108798	0.108798	0.108798	0.141375	0.108798
QARS	0.052047	0.052047	0.052047	0.052047	0.052047	0.052047	0.067631	0.052047
RARS	0.04645	0.04645	0.04645	0.04645	0.04645	0.04645	0.060359	0.04645
FARS	0.029682	0.029682	0.029682	0.029682	0.029682	0.029682	0.03857	0.029682
HARS	0.016006	0.016006	0.016006	0.016006	0.016006	0.016006	0.020799	0.016006
NARS	0.034669	0.034669	0.034669	0.034669	0.034669	0.034669	0.04505	0.034669
ACT	0.000152	0.000152	0.000152	0.000152	0.000152	0.000152	0.000198	0.000152
MYOHC1-2	0	0	0	0	0	0	0	0
MYOHC2a	0.000305	0.000305	0.000305	0.000305	0.000305	0.000305	0.000396	0.000305
MYOHC2b	0	0	0	0	0	0	0	0
MYOLCk	0.000305	0.000305	0.000305	0.000305	0.000305	0.000305	0.000396	0.000305
MYOLCp	0.000305	0.000305	0.000305	0.000305	0.000305	0.000305	0.000396	0.000305
MYO1-2x	0	0	0	0	0	0	0	0
MYO2a	0.000152	0.000152	0.000152	0.000152	0.000152	0.000152	0.000198	0.000152
MYO2x	0	0	0	0	0	0	0	0
MYO2b	0	0	0	0	0	0	0	0
TMN1	0	0	0	0	0	0	0	0
TMN2	4.36E-05	4.36E-05	4.36E-05	4.36E-05	4.36E-05	4.36E-05	5.66E-05	4.36E-05

DIM-TMN1	0	0	0	0	0	0	0	0
DIM-TMN2	2.18E-05	2.18E-05	2.18E-05	2.18E-05	2.18E-05	2.18E-05	2.83E-05	2.18E-05
TPNC1	0	0	0	0	0	0	0	0
TPNC2	2.18E-05	2.18E-05	2.18E-05	2.18E-05	2.18E-05	2.18E-05	2.83E-05	2.18E-05
TPNI1	0	0	0	0	0	0	0	0
TPNI2	2.18E-05	2.18E-05	2.18E-05	2.18E-05	2.18E-05	2.18E-05	2.83E-05	2.18E-05
TPNT1	0	0	0	0	0	0	0	0
TPNT2	2.18E-05	2.18E-05	2.18E-05	2.18E-05	2.18E-05	2.18E-05	2.83E-05	2.18E-05
TPN1	0	0	0	0	0	0	0	0
TPN2	2.18E-05	2.18E-05	2.18E-05	2.18E-05	2.18E-05	2.18E-05	2.83E-05	2.18E-05
CONTR-CO	0	0	0	0	0	0	0	0
CONTR-CO	2.18E-05	2.18E-05	2.18E-05	2.18E-05	2.18E-05	2.18E-05	2.83E-05	2.18E-05
CONTR-CO	0	0	0	0	0	0	0	0
CONTR-CO	0	0	0	0	0	0	0	0
Str_CONTR	0	0	0	0	0	0	0	0
Str_CONTR	2.18E-05	2.18E-05	2.18E-05	2.18E-05	2.18E-05	2.18E-05	2.83E-05	2.18E-05
Str_CONT-(	0	0	0	0	0	0	0	0
Str_CONTR	0	0	0	0	0	0	0	0
CKM	-227.568	-227.568	-229.702	-999999	-231.48	-227.568	-2.19138	-227.568
Spont	227.5677	227.5677	229.7018	999999	231.4802	227.5677	2.191377	227.5677
Nut_cr	0	0	0	0	0	0	6.99E-17	0
ATP2UTP	0	0	0	0	0	0	0	0
UDP2ADP	0	0	0	0	0	0	0	0
PGM1	0	0	0	0	0	0	0	0
UGP2	0	0	0	0	0	0	0	0
mGYG	0	0	0	0	0	0	0	0
mGYS1	0	0	0	0	0	0	0	0
G6PD	0.000005	0.000005	0	0	0.000005	0.000005	0	0.000005
PGLS	0.000005	0.000005	0	0	0.000005	0.000005	0	0.000005
PGD	-5E-06	-5E-06	0	0	-5E-06	-5E-06	0	-5E-06
PGD	0.00001	0.00001	0	0	0.00001	0.00001	0	0.00001
RPE	3.33E-06	3.33E-06	0	0	3.33E-06	3.33E-06	0	3.33E-06
RPIA	1.67E-06	1.67E-06	0	0	1.67E-06	1.67E-06	0	1.67E-06
TKT1	1.67E-06	1.67E-06	0	0	1.67E-06	1.67E-06	0	1.67E-06
TKT2	-1.7E-06	-1.7E-06	0	0	-1.7E-06	-1.7E-06	0	-1.7E-06
TALDO	1.67E-06	1.67E-06	0	0	1.67E-06	1.67E-06	0	1.67E-06
RBSK	0	0	0	0	0	0	0	0
GCL	0	0	0	0	0	0	0	0
GSS	0	0	0	0	0	0	0	0
glutathxpo	0	0	0	0	0	0	0	0
GSR	0	0	0	0	0	0	0	0
GSR	0	0	0	0	0	0	0	0
GlutathLea	0	0	0	0	0	0	0	0
GlutathLea	0	0	0	0	0	0	0	0
Recycle_na	0	0	0	0	0	0	0	0
fa-c6 leak	0	0	0	0	0	0	0	0
acetyLeak(	0.037874	0.037874	0.037874	0	0.215719	0.037874	0.012385	0.037874

0	0	0	0	0	0	0	0

thr	trp	val	ala	asn	asp	cys	glu	gln
**2.18E-05**	**2.18E-05**	**2.18E-05**	**2.18E-05**	**2.18E-05**	**2.18E-05**	**2.18E-05**	**2.18E-05**	**2.18E-05**
0	0	0	0	0	0	0	0	0
-5	-5	-5	0	-5	0	-5	-0.02083	-5
-5	-5	-5	0	-5	0	-5	-0.02083	-5
0	0	0	0.069004	0	0	0	0.457859	0
0	0	0.00001	0	0	0	0	0	0
-0.125	-0.125	0	0	-0.125	-0.125	-0.125	-0.125	-0.125
0	0	0	0	0	0	0	0	0
0	0	0	0	0	0	0	0	0
0	0	0	0	0	0	0	0	0
0	0	0	0	0	0	0	0	0
0	0	0	0	0	0	0	0	0
0	0	0	0	0	0	0	0	0
0	0	0	0	0	0	0	0	0
0	0	0	0	0	0	0	0	0
-0.23	-0.23	0	0	-0.23	-0.23	-0.23	0	-0.23
0	0	0	0	0	0	0	0	0
0	0	0	0	0	0	0	0	0
0	0	0	0	0	0	0	0	0
0	0	0	0	0	0	0	0	0
0	0	0	0	0	0	0	0	0
0	0	0.069004	0	0	0	0	0	0
0	0	9.930986	0	0	0	0	0	0
0	0	0	0	0	0	0	0	0
-39.3733	-39.3733	-184210	-166667	-39.8624	-187500	-39.3733	-187500	-187500
36.40954	36.40954	105249.8	0.072655	36.67631	249994.5	36.40954	125000.1	125010.3
292.4733	292.4733	999999	333334	295.4077	312500.2	292.4733	999999	999999
15	15	15	2.38E-10	15	2.23E-10	15	0.020833	15
-0.05216	-0.05216	-0.01429	-0.01429	-0.05216	-0.05216	-0.05216	-0.01429	-0.01429
-0.23	-0.23	0	0	-0.23	-0.23	-0.23	1.25E-17	-0.23
0	0	0	0	0	0	0	0	0
0	0	0	0	0	0	0	0	0
0	0	0	0	0	0	0	0	0
0	0	0	0	0	0	0	0	0
0	0	0	0	0	0	0	0	0
0	0	0	0	0	0	0	0	0
5	5	5	0	5	0	5	0.020833	5
0	0	0	-0.069	0	0	0	-0.45786	0
0	0	-0.00001	0	0	0	0	0	0
0.125	0.125	0	0	0.125	0.125	0.125	0.125	0.125
0	0	0	0	0	0	0	0	0
0	0	0	0	0	0	0	0	0
0	0	0	0	0	0	0	0	0
0	0	0	0	0	0	0	0	0
0	0	0	0	0	0	0	0	0
0	0	0	0	0	0	0	0	0

0	0	0	0	0	0	0	0	0
0	0	0	0	0	0	0	0	0
0.23	0.23	0	0	0.23	0.23	0.23	0	0.23
0	0	0	0	0	0	0	0	0
0	0	0	0	0	0	0	0	0
0.23	0.23	0	0	0.23	0.23	0.23	-1.2E-17	0.23
0	0	0	0	0	0	0	0	0
0	0	0	0	0	0	0	0	0
0	0	0.069004	0	0	0	0	0	0
0	0	9.930986	0	0	0	0	0	0
5	5	5	0	5	0	5	0.020833	5
4.999995	4.999995	5	0	5	0	4.999995	0.020833	5
4.999998	4.999998	5	0	5	0	4.999998	0.020833	5
0	0	0	0	0	0	0	0	0
4.999998	4.999998	5	0	5	0	4.999998	0.020833	5
-5	-5	-5	0	-5	0	-5	0.020833	-5
9.999998	9.999998	10	0	10	0	9.999998	0	10
9.999998	9.999998	10	0	10	0	9.999998	0	10
9.569031	9.569031	-52620.1	-333333	9.746878	-62496.9	9.569031	-0.38998	9.531159
9.569031	9.569031	-52620.1	-333333	9.746878	-62496.9	9.569031	-0.38998	9.531159
9.746876	9.746876	210529	0	9.746878	0	9.746876	375001.4	374996.5
0	0	210529.2	333332.6	0	0.253122	0	375001.7	374986.7
0	0	0	0	0	0	0	0	0
0.177845	0.177845	52619.88	0	0	62496.67	0.177845	0.136861	0.215719
0	0	0	-0.069	0	0	0	-0.45786	0
10.43098	10.43098	52640.14	333332.5	10.25312	62496.83	10.43098	-0.13686	10.46884
10.60882	10.60882	105269.9	333332.5	10.25312	124993.5	10.60882	0	10.68456
0	0	0	0	0	0	0	0.041667	0
10.43098	10.43098	52640.14	333332.5	10.25312	62496.83	10.43098	-0.13686	10.46884
11.22473	11.22473	105270	333332.5	11.04688	62497.5	11.22473	124999.8	124985.3
9.999998	9.999998	0	0	10	62497.06	9.999998	0	10
11.22473	11.22473	105270	333332.5	11.04688	62497.5	11.22473	124999.8	124985.3
0	0	0	0	0	0	0	0	0
9.999998	9.999998	0	0	10	62497.06	9.999998	0	10
12.70003	12.70003	0.014287	0.014287	12.87788	62499.96	12.70003	0.014287	12.62429
12.70003	12.70003	0.014287	0.014287	12.87788	62499.96	12.70003	0.014287	12.62429
0	0	0	0	0	0	0	0	0
12.70003	12.70003	0.014287	0.014287	12.87788	62499.96	12.70003	0.014287	12.62429
13.2887	13.2887	0	0	13.46655	62500.61	13.2887	124999.9	124987.3
13.45591	13.45591	52629.87	0.058368	13.63376	62500.59	13.45591	124999.9	124987.5
13.49379	13.49379	52629.87	0.058368	13.67163	62500.63	13.49379	124999.9	124987.5
13.49379	13.49379	52629.87	0.058368	13.67163	62500.63	13.49379	124999.9	124987.5
23.92476	23.92476	105270	333332.5	23.92475	124997.5	23.92476	124999.8	124998
49.89181	49.89181	263159.7	333332.8	50.2475	249997.4	49.89181	249999.7	249997.9
28.85488	28.85488	105259.9	0.256253	29.47734	125003.3	28.85488	124999.9	125002.7
78.74669	78.74669	368419.6	333333.1	79.72484	375000.8	78.74669	374999.6	375000.6
39.37335	39.37335	184209.8	166666.5	39.86242	187500.4	39.37335	187499.8	187500.3

207.3852	207.3852	999999	999999	209.6972	999999	207.3852	999999	999999
1.603164	1.603164	210530.1	333333.5	1.781009	531250.1	1.603164	437501.3	499972.5
0.125	0.125	0	0	0.125	0.125	0.125	0.125	0.125
0.125	0.125	0	0	0.125	0.125	0.125	0	0.125
0.125	0.125	0	0	0.125	0.125	0.125	0	0.125
0.125	0.125	0	0	0.125	0.125	0.125	0	0.125
0	0	0	0	0	0	0	0	0
0	0	0	0	0	0	0	0	0
0	0	0	0	0	0	0	0	0
0	0	0	0	0	0	0	0	0
0.125	0.125	0	0	0.125	0.125	0.125	0	0.125
0.125	0.125	0	0	0.125	0.125	0.125	0	0.125
0.125	0.125	0	0	0.125	0.125	0.125	0	0.125
0.125	0.125	0	0	0.125	0.125	0.125	0	0.125
0	0	0	0	0	0	0	0	0
0	0	0	0	0	0	0	0	0
0	0	0	0	0	0	0	0	0
0	0	0	0	0	0	0	0	0
0.355	0.355	0	0	0.355	0.355	0.355	0	0.355
0.355	0.355	0	0	0.355	0.355	0.355	0	0.355
0.355	0.355	0	0	0.355	0.355	0.355	0	0.355
0.355	0.355	0	0	0.355	0.355	0.355	0	0.355
0.355	0.355	0	0	0.355	0.355	0.355	0	0.355
0.355	0.355	0	0	0.355	0.355	0.355	0	0.355
0.355	0.355	0	0	0.355	0.355	0.355	0	0.355
0.355	0.355	0	0	0.355	0.355	0.355	0	0.355
0.355	0.355	0	0	0.355	0.355	0.355	0	0.355
0.355	0.355	0	0	0.355	0.355	0.355	0	0.355
0.355	0.355	0	0	0.355	0.355	0.355	0	0.355
0.355	0.355	0	0	0.355	0.355	0.355	0	0.355
0.355	0.355	0	0	0.355	0.355	0.355	0	0.355
0.355	0.355	0	0	0.355	0.355	0.355	0	0.355
0.355	0.355	0	0	0.355	0.355	0.355	0	0.355
0.355	0.355	0	0	0.355	0.355	0.355	0	0.355
0.355	0.355	0	0	0.355	0.355	0.355	0	0.355
0.355	0.355	0	0	0.355	0.355	0.355	0	0.355
0.355	0.355	0	0	0.355	0.355	0.355	0	0.355
0.355	0.355	0	0	0.355	0.355	0.355	0	0.355
0.355	0.355	0	0	0.355	0.355	0.355	0	0.355
0.355	0.355	0	0	0.443922	0.453017	0.355	0	0.355
0.355	0.355	0	0	0.443922	0.453017	0.355	0	0.355
0.392874	0.392874	0	0	0.481796	0.490891	0.392874	0	0.355
0	0	0	0	0	0	0	0	0
0	0	0	0	0	0	0	0.041667	0
0	0	0	0	0	0	0	0.041667	0
0	0	0	0	0	0	0	0.041667	0
0	0	0	0	0	0	0	0.041667	0

0	0	0	0	0	0	0	0.041667	0
0	0	0	0	0	0	0	0	0
0	0	0	0	0	0	0	0	0
0	0	0	0	0	0	0	0	0
0	0	0	0	0	0	0	0	0
0	0	0	0	0	0	0	0	0
0	0	0	0	0	0	0	0	0
0	0	0	0	0	0	0	0	0
0	0	0	0	0	0	0	0	0
0.23	0.23	0	0	0.23	0.23	0.23	0	0.23
0	0	0	0	0	0	0	0	0
0	0	0	0	0	0	0	0	0
0	0	0	0	0	0	0	0	0
0	0	0	0	0	0	0	0	0
0	0	0	0	0	0	0	0	0
0	0	0	0	0	0	0	0	0
0	0	0	0	0	0	0	0	0
0	0	0	0	0	0	0	0	0
0	0	0	0	0	0	0	0	0
0.23	0.23	0	0	0.23	0.23	0.23	0	0.23
0	0	0	0	0	0	0	0	0
0	0	0	0	0	0	0	0	0
0	0	0	0	0	0	0	0	0
0	0	0	0	0	0	0	0	0
0	0	0	0	0	0	0	0	0
0	0	0	0	0	0	0	0	0
0	0	0	0	0	0	0	0	0
0	0	0	0	0	0	0	0	0
0	0	0	0	0	0	0	0	0
0.23	0.23	0	0	0.23	0.23	0.23	0	0.23
0	0	0	0	0	0	0	0	0
0	0	0	0	0	0	0	0	0
0	0	0	0	0	0	0	0	0
0	0	0	0	0	0	0	0	0
0	0	0	0	0	0	0	0	0
0	0	0	0	0	0	0	0	0
0	0	0	0	0	0	0	0	0
0	0	0	0	0	0	0	0	0
0	0	0	0	0	0	0	0	0
0.23	0.23	0	0	0.23	0.23	0.23	0	0.23
0	0	0	0	0	0	0	0	0
0	0	0	0	0	0	0	0	0
0	0	0	0	0	0	0	0	0
0	0	0	0	0	0	0	0	0
0	0	0	0	0	0	0	0	0
0	0	0	0	0	0	0	0	0
0	0	0	0	0	0	0	0	0

0	0	0	0	0	0	0	0	0
0	0	0	0	0	0	0	0	0
0	0	0	0	0	0	0	0	0
0	0	0	0	0	0	0	0	0
0	0	0	0	0	0	0	0	0
0	0	0	0	0	0	0	0	0
0	0	0	0	0	0	0	0	0
0	0	0	0	0	0	0	0	0
0	0	0	0	0	0	0	0	0
-0.04645	-0.04645	-0.04645	-0.04645	-0.04645	-0.04645	-0.04645	-0.04645	-0.04645
-0.01601	-0.01601	-0.01601	-0.01601	-0.01601	-0.082	-0.01601	-0.01601	-0.01601
-0.062	-0.062	-0.062	-0.062	-0.062	-0.062	-0.062	-0.062	-0.062
-0.123	-0.123	-0.08513	-0.08513	-0.123	-0.123	-0.123	-0.08513	-0.08513
-0.08998	-0.08998	-0.08998	-0.08998	-0.17891	-0.188	-0.08998	-0.08998	-0.08998
-0.025	-0.025	-0.025	-0.025	-0.025	-0.025	-0.025	-0.025	-0.025
-0.02968	-0.02968	-0.02968	-0.057	-0.02968	-0.057	-0.02968	-0.057	-0.02968
-0.04734	-0.04734	-0.04734	-0.04734	-0.04734	-0.04734	-0.04734	-0.04734	-0.04734
-0.00494	-0.00494	-0.00494	-0.00494	-0.00494	-0.00494	-0.00494	-0.00494	-0.00494
-0.233	-0.233	-52629.9	-0.08629	-0.233	-0.0422	-0.233	-0.0422	-0.233
-0.333	-0.333	-0.333	-333333	-0.333	-0.333	-0.333	-0.333	-0.333
0.537158	0.537158	-0.03467	-0.03467	0.715003	62497.93	0.537158	-0.041	124974.6
-0.003	-0.003	-0.003	-0.003	-0.003	-124994	-0.003	124999.7	-0.003
-0.01028	-0.01028	-0.01028	-0.01028	-0.01028	-0.01028	-0.01028	-0.01028	-0.01028
-0.024	-0.024	-0.024	-0.024	-0.024	-0.024	-0.024	-125000	-0.024
-0.586	-0.586	-0.586	-0.586	-0.586	-0.586	-0.586	-0.586	-124975
-0.04715	-0.04715	-0.04715	-0.04715	-0.04715	-0.11314	-0.04715	-0.04715	-0.04715
-0.168	-0.168	-0.168	-0.168	-0.168	-0.168	-0.168	-0.02848	-0.168
0.384843	0.384843	52630.09	333332.5	0.206998	-0.114	0.384843	-0.114	0.422717
-0.01738	-0.01738	-0.01738	0.00994	-0.01738	0.00994	-0.01738	0.00994	-0.01738
0.242956	0.242956	52629.87	0.058368	0.242956	0.05216	0.242956	0.014287	0.205083
0.190796	0.190796	52629.86	0.044082	0.190796	0	0.190796	0	0.190796
0.190796	0.190796	52629.86	0.044082	0.190796	0	0.190796	0	0.190796
0.190796	0.190796	52629.86	0.044082	0.190796	0	0.190796	0	0.190796
0.190796	0.190796	52629.86	0.044082	0.190796	0	0.190796	0	0.190796
0.190796	0.190796	52629.86	0.044082	0.190796	0	0.190796	0	0.190796
0.190796	0.190796	52629.86	0.044082	0.190796	0	0.190796	0	0.190796
0.190796	0.190796	52629.86	0.044082	0.190796	0	0.190796	0	0.190796
0.190796	0.190796	52629.86	0.044082	0.190796	0	0.190796	0	0.190796
0.190796	0.190796	52629.86	0.044082	0.190796	0	0.190796	0	0.190796
0.205083	0.205083	52629.87	0.058368	0.205083	0.014287	0.205083	0.014287	0.205083
0.205083	0.205083	52629.87	0.058368	0.205083	0.014287	0.205083	0.014287	0.205083
0.205083	0.205083	52629.87	0.058368	0.205083	0.014287	0.205083	0.014287	0.205083
0.014287	0.014287	0.014287	0.014287	0.014287	0.014287	0.014287	0.014287	0.014287
0.014287	0.014287	0.014287	0.014287	0.014287	0.014287	0.014287	0.014287	0.014287
0.014287	0.014287	0.014287	0.014287	0.014287	0.014287	0.014287	0.014287	0.014287
0.014287	0.014287	0.014287	0.014287	0.014287	0.014287	0.014287	0.014287	0.014287
0.014287	0.014287	0.014287	0.014287	0.014287	0.014287	0.014287	0.014287	0.014287

0.014287	0.014287	0.014287	0.014287	0.014287	0.014287	0.014287	0.014287	0.014287
0.014287	0.014287	0.014287	0.014287	0.014287	0.014287	0.014287	0.014287	0.014287
0.037874	0.037874	-1.2E-11	-5.4E-18	0.037874	0.037874	0.037874	1.8E-18	-2.7E-17
0.037874	0.037874	-1.2E-11	-5.4E-18	0.037874	0.037874	0.037874	1.8E-18	-2.7E-17
0.037874	0.037874	-1.2E-11	-5.4E-18	0.037874	0.037874	0.037874	1.8E-18	-2.7E-17
0.037874	0.037874	0	0	0.037874	0.037874	0.037874	0	0
0.037874	0.037874	0	0	0.037874	0.037874	0.037874	0	0
0.037874	0.037874	0	0	0.037874	0.037874	0.037874	0	0
0.037874	0.037874	0	0	0.037874	0.037874	0.037874	0	0
0.037874	0.037874	0	0	0.037874	0.037874	0.037874	0	0
0.037874	0.037874	0	0	0.037874	0.037874	0.037874	0	0
0	0	0	0	0	0	0	0	0
0.037874	0.037874	-1.4E-11	3.07E-11	0.215719	0.233909	0.037874	2.04E-12	2.27E-12
0.037874	0.037874	-1.4E-11	0.069004	0.215719	531248.3	0.037874	62499.09	10.1291
0	0	0.533953	0.602957	0	0	0	0.998143	0
-0.25312	-0.25312	-0.25312	-333333	-0.25312	-0.25312	-0.25312	-0.25312	-0.25312
0	0	0	0	0	0	0	0	0
0	0	0	0.069004	0	62497.06	0	0.457859	0
0	0	0	0	0	0	0	0	0
0	0	0	0	0	0	0	0	0
0	0	0	0	0	0	0	0	0
0	0	0	0	0	0	0	0	0
0	0	0	0	0.088922	0.098017	0	0	0
0	0	0	0	0.088922	0.098017	0	0	0
0	0	0	0	0.088922	0.098017	0	0	0
0	0	0	0	0.088922	0.098017	0	0	0
0	0	0	0	0.088922	0.098017	0	0	0
0	0	0	0	0.088922	0.098017	0	0	0
0	0	0	0	0.088922	0.098017	0	0	0
0.139516	0.139516	0.139516	0.139516	0.139516	0.139516	0.139516	0	0.139516
12.87742	12.87742	0.153802	0.153802	13.23311	62500.33	12.87742	0.014287	12.7638
0.139516	0.139516	0.139516	0.139516	0.139516	0.139516	0.139516	0	0.139516
0	0	0	0	0	0	0	0	0
0	0	0	0	0	0	0	0	0
0	0	0	0	0	0	0	0	0
0	0	0	0	0	0	0	0	0
0	0	0	0	0	0	0	0	0
0	0	0	0	0	0	0	0	0
0	0	0	0	0	0	0	0	0
0	0	0	0	0	-0.06599	0	0	0
0	0	0	0	0	0	0	0	0
0	0	0	0	0	0	0	0	0
0	0	0	0	0	0.065994	0	0	0
0	0	0	0	0	0.065994	0	0	0
0	0	0	0	0	0.065994	0	0	0
0	0	0	0	0	0.065994	0	0	0
0	0	0	0	0	0.065994	0	0	0

0	0	0	0	0	0.065994	0	0	0
0	0	0	0	0	468750.9	0	62498.63	10.1291
0.571827	0.571827	0	0	0.749672	531248.8	0.571827	62498.62	124984.7
0.430967	0.430967	52630.14	333332.6	0.253122	62496.93	0.430967	0.389983	0.468841
0.430967	0.430967	52630.14	333332.6	0.253122	62496.93	0.430967	0.389983	0.468841
0.430967	0.430967	52630.14	333332.6	0.253122	62496.93	0.430967	0.389983	0.468841
0	0	0	0	0	0	0	0	0
0	0	0	0	0	0	0	0	0
0	0	0	0	0	0	0	0	0
12.73791	12.73791	0.014287	0.014287	13.0936	62500.19	12.73791	0.014287	12.62429
0	0	0	0.027318	0	0.027318	0	0.027318	0
0	0	0	0.027318	0	0.027318	0	0.027318	0
0	0	0	0.027318	0	0.027318	0	0.027318	0
0.344598	0.344598	52630.01	0.197884	0.344598	0.153802	0.344598	0.014287	0.344598
1.22E-16	1.22E-16	-9.4E-18	3.01E-18	3.43E-17	4.06E-17	8.49E-17	-3.1E-18	-1.2E-16
0.017378	0.017378	0.017378	0.017378	0.017378	0.017378	0.017378	0.017378	0.017378
0.004943	0.004943	0.004943	0.004943	0.004943	0.004943	0.004943	0.004943	0.004943
0.047343	0.047343	0.047343	0.047343	0.047343	0.047343	0.047343	0.047343	0.047343
0.085126	0.085126	0.085126	0.085126	0.085126	0.085126	0.085126	0.085126	0.085126
0.047713	0.047713	0.047713	0.047713	0.047713	0.047713	0.047713	0.047713	0.047713
0.089983	0.089983	0.089983	0.089983	0.089983	0.089983	0.089983	0.089983	0.089983
0.079878	0.079878	0.079878	0.079878	0.079878	0.079878	0.079878	0.079878	0.079878
0.042204	0.042204	0.042204	0.042204	0.042204	0.042204	0.042204	0.042204	0.042204
0.025	0.025	0.025	0.025	0.025	0.025	0.025	0.025	0.025
0.046124	0.046124	0.046124	0.046124	0.046124	0.046124	0.046124	0.046124	0.046124
0.047082	0.047082	0.047082	0.047082	0.047082	0.047082	0.047082	0.047082	0.047082
0.047147	0.047147	0.047147	0.047147	0.047147	0.047147	0.047147	0.047147	0.047147
0.028484	0.028484	0.028484	0.028484	0.028484	0.028484	0.028484	0.028484	0.028484
0.010279	0.010279	0.010279	0.010279	0.010279	0.010279	0.010279	0.010279	0.010279
0.108798	0.108798	0.108798	0.108798	0.108798	0.108798	0.108798	0.108798	0.108798
0.052047	0.052047	0.052047	0.052047	0.052047	0.052047	0.052047	0.052047	0.052047
0.04645	0.04645	0.04645	0.04645	0.04645	0.04645	0.04645	0.04645	0.04645
0.029682	0.029682	0.029682	0.029682	0.029682	0.029682	0.029682	0.029682	0.029682
0.016006	0.016006	0.016006	0.016006	0.016006	0.016006	0.016006	0.016006	0.016006
0.034669	0.034669	0.034669	0.034669	0.034669	0.034669	0.034669	0.034669	0.034669
0.000152	0.000152	0.000152	0.000152	0.000152	0.000152	0.000152	0.000152	0.000152
0	0	0	0	0	0	0	0	0
0.000305	0.000305	0.000305	0.000305	0.000305	0.000305	0.000305	0.000305	0.000305
0	0	0	0	0	0	0	0	0
0.000305	0.000305	0.000305	0.000305	0.000305	0.000305	0.000305	0.000305	0.000305
0.000305	0.000305	0.000305	0.000305	0.000305	0.000305	0.000305	0.000305	0.000305
0	0	0	0	0	0	0	0	0
0.000152	0.000152	0.000152	0.000152	0.000152	0.000152	0.000152	0.000152	0.000152
0	0	0	0	0	0	0	0	0
0	0	0	0	0	0	0	0	0
0	0	0	0	0	0	0	0	0
4.36E-05	4.36E-05	4.36E-05	4.36E-05	4.36E-05	4.36E-05	4.36E-05	4.36E-05	4.36E-05

0	0	0	0	0	0	0	0	0
2.18E-05	2.18E-05	2.18E-05	2.18E-05	2.18E-05	2.18E-05	2.18E-05	2.18E-05	2.18E-05
0	0	0	0	0	0	0	0	0
2.18E-05	2.18E-05	2.18E-05	2.18E-05	2.18E-05	2.18E-05	2.18E-05	2.18E-05	2.18E-05
0	0	0	0	0	0	0	0	0
2.18E-05	2.18E-05	2.18E-05	2.18E-05	2.18E-05	2.18E-05	2.18E-05	2.18E-05	2.18E-05
0	0	0	0	0	0	0	0	0
2.18E-05	2.18E-05	2.18E-05	2.18E-05	2.18E-05	2.18E-05	2.18E-05	2.18E-05	2.18E-05
0	0	0	0	0	0	0	0	0
2.18E-05	2.18E-05	2.18E-05	2.18E-05	2.18E-05	2.18E-05	2.18E-05	2.18E-05	2.18E-05
0	0	0	0	0	0	0	0	0
2.18E-05	2.18E-05	2.18E-05	2.18E-05	2.18E-05	2.18E-05	2.18E-05	2.18E-05	2.18E-05
0	0	0	0	0	0	0	0	0
0	0	0	0	0	0	0	0	0
0	0	0	0	0	0	0	0	0
2.18E-05	2.18E-05	2.18E-05	2.18E-05	2.18E-05	2.18E-05	2.18E-05	2.18E-05	2.18E-05
0	0	0	0	0	0	0	0	0
0	0	0	0	0	0	0	0	0
-227.568	-227.568	-999997	-666665	-229.702	0	-227.568	-999999	-999999
227.5677	227.5677	999996.9	666664.6	229.7018	0	227.5677	999999	999999
0	0	0	0	0	0	0	0	-4.6E-11
0	0	0	0	0	0	0	0	0
0	0	0	0	0	0	0	0	0
0	0	0	0	0	0	0	0	0
0	0	0	0	0	0	0	0	0
0	0	0	0	0	0	0	0	0
0	0	0	0	0	0	0	0	0
0.000005	0.000005	0	0	0	0	0.000005	0	0
0.000005	0.000005	0	0	0	0	0.000005	0	0
-5E-06	-5E-06	0	0	0	0	-5E-06	0	0
0.00001	0.00001	0	0	0	0	0.00001	0	0
3.33E-06	3.33E-06	0	0	0	0	3.33E-06	0	0
1.67E-06	1.67E-06	0	0	0	0	1.67E-06	0	0
1.67E-06	1.67E-06	0	0	0	0	1.67E-06	0	0
-1.7E-06	-1.7E-06	0	0	0	0	-1.7E-06	0	0
1.67E-06	1.67E-06	0	0	0	0	1.67E-06	0	0
0	0	0	0	0	0	0	0	0
0	0	0	0	0	0	0	0	0
0	0	0	0	0	0	0	0	0
0	0	0	0	0	0	0	0	0
0	0	0	0	0	0	0	0	0
0	0	0	0	0	0	0	0	0
0	0	0	0	0	0	0	0	0
0	0	0	0	0	0	0	0	0
0	0	0	0	0	0	0	0	0
0	0	0	0	0	0	0	0	0
0.037874	0.037874	0	0	0.037874	0.037874	0.037874	0	0

0	0	0	0	0	0	0	0	0

gly	pro	ser	tyr
**2.18E-05**	**2.18E-05**	**2.18E-05**	**2.18E-05**
0	0	0	0
-5	0	-5	-5
-5	0	-5	-5
0	0.253122	0	0
0	0	0	0
-0.125	0	-0.125	-0.125
0	0	0	0
0	0	0	0
0	0	0	0
0	0	0	0
0	0	0	0
0	0	0	0
0	0	0	0
0	0	0	0
-0.23	0	-0.23	-0.23
0	0	0	0
0	0	0	0
0	0	0	0
0	0	0	0
0	0	0	0
0	0	0	0
0	0	0	0
0	0	0	0
-39.3733	-200000	-39.3733	-39.3733
36.40954	0.072655	36.40954	36.40954
292.4733	999998.5	292.4733	292.4733
15	-1.6E-10	15	15
-0.05216	-0.01429	-0.05216	-0.05216
-0.23	0	-0.23	-0.23
0	0	0	0
0	0	0	0
0	0	0	0
0	0	0	0
0	0	0	0
0	0	0	0
5	0	5	5
0	-0.25312	0	0
0	0	0	0
0.125	0	0.125	0.125
0	0	0	0
0	0	0	0
0	0	0	0
0	0	0	0
0	0	0	0
0	0	0	0

0	0	0	0
0	0	0	0
0.23	0	0.23	0.23
0	0	0	0
0	0	0	0
0.23	0	0.23	0.23
0	0	0	0
0	0	0	0
0	0	0	0
0	0	0	0
5	0	5	5
4.999995	0	4.999995	4.999995
4.999998	0	4.999998	4.999998
0	0	0	0
4.999998	0	4.999998	4.999998
-5	0	-5	-5
9.999998	0	9.999998	9.999998
9.999998	0	9.999998	9.999998
9.569031	-0.25312	9.569031	9.569031
9.569031	-0.25312	9.569031	9.569031
9.746876	0	9.746876	9.746876
0	0.253122	0	0
0	0	0	0
0.177845	0	0.177845	0.177845
0	-0.25312	0	0
10.43098	0	10.43098	10.43098
10.60882	0	10.60882	10.60882
0	0	0	0
10.43098	0	10.43098	10.43098
11.22473	0.044082	11.22473	11.22473
9.999998	0	9.999998	9.999998
11.22473	0.044082	11.22473	11.22473
0	0	0	0
9.999998	0	9.999998	9.999998
12.70003	0.014287	12.70003	12.70003
12.70003	0.014287	12.70003	12.70003
0	0	0	0
12.70003	0.014287	12.70003	12.70003
13.2887	0.044082	13.2887	13.2887
13.45591	0.058368	13.45591	13.45591
13.49379	0.058368	13.49379	13.49379
13.49379	0.058368	13.49379	13.49379
23.92476	0.058368	23.92476	23.92476
49.89181	199999.8	49.89181	49.89181
28.85488	199999.8	28.85488	28.85488
78.74669	399999.6	78.74669	78.74669
39.37335	199999.8	39.37335	39.37335

207.3852	999999	207.3852	207.3852
1.603164	1.159459	1.603164	1.603164
0.125	0	0.125	0.125
0.125	0	0.125	0.125
0.125	0	0.125	0.125
0.125	0	0.125	0.125
0	0	0	0
0	0	0	0
0	0	0	0
0	0	0	0
0.125	0	0.125	0.125
0.125	0	0.125	0.125
0.125	0	0.125	0.125
0.125	0	0.125	0.125
0	0	0	0
0	0	0	0
0	0	0	0
0	0	0	0
0.355	0	0.355	0.355
0.355	0	0.355	0.355
0.355	0	0.355	0.355
0.355	0	0.355	0.355
0.355	0	0.355	0.355
0.355	0	0.355	0.355
0.355	0	0.355	0.355
0.355	0	0.355	0.355
0.355	0	0.355	0.355
0.355	0	0.355	0.355
0.355	0	0.355	0.355
0.355	0	0.355	0.355
0.355	0	0.355	0.355
0.355	0	0.355	0.355
0.355	0	0.355	0.355
0.355	0	0.355	0.355
0.355	0	0.355	0.355
0.355	0	0.355	0.355
0.355	0	0.355	0.355
0.355	0	0.355	0.355
0.355	0	0.355	0.355
0.355	0	0.355	0.355
0.355	0	0.355	0.355
0.355	0	0.355	0.355
0.392874	0	0.392874	0.392874
0	0	0	0
0	0	0	0
0	0	0	0
0	0	0	0
0	0	0	0

0	0	0	0
0	0	0	0
0	0	0	0
0	0	0	0
0	0	0	0
0	0	0	0
0	0	0	0
0	0	0	0
0	0	0	0
0.23	0	0.23	0.23
0	0	0	0
0	0	0	0
0	0	0	0
0	0	0	0
0	0	0	0
0	0	0	0
0	0	0	0
0	0	0	0
0	0	0	0
0.23	0	0.23	0.23
0	0	0	0
0	0	0	0
0	0	0	0
0	0	0	0
0	0	0	0
0	0	0	0
0	0	0	0
0	0	0	0
0	0	0	0
0.23	0	0.23	0.23
0	0	0	0
0	0	0	0
0	0	0	0
0	0	0	0
0	0	0	0
0	0	0	0
0	0	0	0
0	0	0	0
0	0	0	0
0.23	0	0.23	0.23
0	0	0	0
0	0	0	0
0	0	0	0
0	0	0	0
0	0	0	0
0	0	0	0
0	0	0	0

0	0	0	0
0	0	0	0
0	0	0	0
0	0	0	0
0	0	0	0
0	0	0	0
0	0	0	0
0	0	0	0
0	0	0	0
-0.04645	-0.04645	-0.04645	-0.04645
-0.01601	-0.01601	-0.01601	-0.01601
-0.062	-0.062	-0.062	-0.062
-0.123	-0.08513	-0.123	-0.123
-0.08998	-0.08998	-0.08998	-0.08998
-0.025	-0.025	-0.025	-0.025
-0.02968	-0.02968	-0.02968	-0.02968
-0.04734	-0.04734	-0.04734	-0.04734
-0.00494	-0.00494	-0.00494	-0.00494
-0.233	-0.0422	-0.233	-0.233
-0.333	-0.333	-0.333	-0.333
0.537158	-0.03467	0.537158	0.537158
-0.003	-0.003	-0.003	-0.003
-0.01028	-0.01028	-0.01028	-0.01028
-0.024	199999.3	-0.024	-0.024
-0.586	-0.586	-0.586	-0.586
-0.04715	-0.04715	-0.04715	-0.04715
-0.168	-200000	-0.168	-0.168
0.384843	-0.04612	0.384843	0.384843
-0.01738	-0.01738	-0.01738	-0.01738
0.242956	0.014287	0.242956	0.242956
0.190796	0	0.190796	0.190796
0.190796	0	0.190796	0.190796
0.190796	0	0.190796	0.190796
0.190796	0	0.190796	0.190796
0.190796	0	0.190796	0.190796
0.190796	0	0.190796	0.190796
0.190796	0	0.190796	0.190796
0.190796	0	0.190796	0.190796
0.190796	0	0.190796	0.190796
0.205083	0.014287	0.205083	0.205083
0.205083	0.014287	0.205083	0.205083
0.205083	0.014287	0.205083	0.205083
0.014287	0.014287	0.014287	0.014287
0.014287	0.014287	0.014287	0.014287
0.014287	0.014287	0.014287	0.014287
0.014287	0.014287	0.014287	0.014287
0.014287	0.014287	0.014287	0.014287

0.014287	0.014287	0.014287	0.014287
0.014287	0.014287	0.014287	0.014287
0.037874	1.8E-18	0.037874	0.037874
0.037874	1.8E-18	0.037874	0.037874
0.037874	1.8E-18	0.037874	0.037874
0.037874	0	0.037874	0.037874
0.037874	0	0.037874	0.037874
0.037874	0	0.037874	0.037874
0.037874	0	0.037874	0.037874
0.037874	0	0.037874	0.037874
0.037874	0	0.037874	0.037874
0	0	0	0
0.037874	-2.4E-18	0.037874	0.037874
0.037874	0.253122	0.037874	0.037874
0	0.787075	0	0
-0.25312	-0.25312	-0.25312	-0.25312
0	0	0	0
0	0.253122	0	0
0	0	0	0
0	0	0	0
0	0	0	0
0	0	0	0
0	0	0	0
0	0	0	0
0	0	0	0
0	0	0	0
0	0	0	0
0	0	0	0
0	0	0	0
0.139516	199999.7	0.139516	0.139516
12.87742	199999.7	12.87742	12.87742
0.139516	199999.7	0.139516	0.139516
0	0	0	0
0	0	0	0
0	0	0	0
0	0	0	0
0	0	0	0
0	0	0	0
0	0	0	0
0	0	0	0
0	0	0	0
0	0	0	0
0	0	0	0
0	0	0	0
0	0	0	0
0	0	0	0
0	0	0	0

0	0	0	0
0	0	0	0
0.571827	0	0.571827	0.571827
0.430967	0.253122	0.430967	0.430967
0.430967	0.253122	0.430967	0.430967
0.430967	0.253122	0.430967	0.430967
0	0	0	0
0	0	0	0
0	0	0	0
12.73791	0.014287	12.73791	12.73791
0	0	0	0
0	0	0	0
0	0	0	0
0.344598	199999.7	0.344598	0.344598
1.37E-16	6.84E-17	1.22E-16	1.22E-16
0.017378	0.017378	0.017378	0.017378
0.004943	0.004943	0.004943	0.004943
0.047343	0.047343	0.047343	0.047343
0.085126	0.085126	0.085126	0.085126
0.047713	0.047713	0.047713	0.047713
0.089983	0.089983	0.089983	0.089983
0.079878	0.079878	0.079878	0.079878
0.042204	0.042204	0.042204	0.042204
0.025	0.025	0.025	0.025
0.046124	0.046124	0.046124	0.046124
0.047082	0.047082	0.047082	0.047082
0.047147	0.047147	0.047147	0.047147
0.028484	0.028484	0.028484	0.028484
0.010279	0.010279	0.010279	0.010279
0.108798	0.108798	0.108798	0.108798
0.052047	0.052047	0.052047	0.052047
0.04645	0.04645	0.04645	0.04645
0.029682	0.029682	0.029682	0.029682
0.016006	0.016006	0.016006	0.016006
0.034669	0.034669	0.034669	0.034669
0.000152	0.000152	0.000152	0.000152
0	0	0	0
0.000305	0.000305	0.000305	0.000305
0	0	0	0
0.000305	0.000305	0.000305	0.000305
0.000305	0.000305	0.000305	0.000305
0	0	0	0
0.000152	0.000152	0.000152	0.000152
0	0	0	0
0	0	0	0
0	0	0	0
4.36E-05	4.36E-05	4.36E-05	4.36E-05

0	0	0	0
2.18E-05	2.18E-05	2.18E-05	2.18E-05
0	0	0	0
2.18E-05	2.18E-05	2.18E-05	2.18E-05
0	0	0	0
2.18E-05	2.18E-05	2.18E-05	2.18E-05
0	0	0	0
2.18E-05	2.18E-05	2.18E-05	2.18E-05
0	0	0	0
2.18E-05	2.18E-05	2.18E-05	2.18E-05
0	0	0	0
2.18E-05	2.18E-05	2.18E-05	2.18E-05
0	0	0	0
0	0	0	0
0	0	0	0
2.18E-05	2.18E-05	2.18E-05	2.18E-05
0	0	0	0
0	0	0	0
-227.568	-999997	-227.568	-227.568
227.5677	999996.7	227.5677	227.5677
0	0	0	0
0	0	0	0
0	0	0	0
0	0	0	0
0	0	0	0
0	0	0	0
0	0	0	0
0.000005	0	0.000005	0.000005
0.000005	0	0.000005	0.000005
-5E-06	0	-5E-06	-5E-06
0.00001	0	0.00001	0.00001
3.33E-06	0	3.33E-06	3.33E-06
1.67E-06	0	1.67E-06	1.67E-06
1.67E-06	0	1.67E-06	1.67E-06
-1.7E-06	0	-1.7E-06	-1.7E-06
1.67E-06	0	1.67E-06	1.67E-06
0	0	0	0
0	0	0	0
0	0	0	0
0	0	0	0
0	0	0	0
0	0	0	0
0	0	0	0
0	0	0	0
0	0	0	0
0	0	0	0
0.037874	0	0.037874	0.037874

0	0	0	0
