# Supplementary material for: To Supplement or Not to Supplement: A Metabolic Network Framework for Human Nutritional Supplements
Source: PLoS One. 2013 Aug 5;8(8):e68751. doi: 10.1371/journal.pone.0068751 (PMC3740736; doi:10.1371/journal.pone.0068751)
Supplement: File S2 — Muscle Cell Model. (PDF) [file pone.0068751.s003.pdf]

| 0 | Enzyme | Abrev | EC Number | Location | Pathway | Reaction | Lower Bound | Upper Bound | Thermodynamics |
|---|---|---|---|---|---|---|---|---|---|
| 1 | ATP Objextive Flux | Obj_atp | | | Objecitve | atp --> | 0 | 999999 | |
| 2 | Insulin Availiblity | Ex_ins | | | Signaling/Hormone | insulin[e] <==> | -999999 | 0 | |
| 3 | Glucose Exchange | Ex_gluc | | | Exchange | gluc[e] <==> | -999999 | 999999 | |
| 4 | Lactate Exchange | Ex_lac | | | Exchange | lac[e] <==> | -999999 | 999999 | |
| 5 | Pyruvate Exchange | Ex_pyr | | | Exchange | pyr[e] <==> | 0 | 0 | |
| 6 | Palmitate Exchange | Ex_pal | | | Exchange | pal[e] <==> | -999999 | 999999 | |
| 7 | Propanoate Input | Ex_prop | | | Exchange | prop[e] <==> | 0 | 0 | |
| 8 | Butanoate Input | Ex_but | | | Exchange | but[e] <==> | 0 | 0 | |
| 9 | Pentanoate Input | Ex_5c | | | Exchange | 5c-fa[e] <==> | 0 | 0 | |
| 10 | Hexanoate Input | Ex_6c | | | Exchange | 6c-fa[e] <==> | 0 | 0 | |
| 11 | Octanoate Input | Ex_8c | | | Exchange | 8c-fa[e] <==> | 0 | 0 | |
| 12 | Nonanoate Input | Ex_9c | | | Exchange | 9c-fa[e] <==> | 0 | 0 | |
| 13 | Decanoate Input | Ex_10c | | | Exchange | 10c-fa[e] <==> | 0 | 0 | |
| 14 | Dodecanoate Input | Ex_12c | | | Exchange | 12c-fa[e] <==> | 0 | 0 | |
| 15 | Tetradecanoate Input | Ex_14c | | | Exchange | 14c-fa[e] <==> | 0 | 0 | |
| 16 | Octadecanoate Input | Ex_18c | | | Exchange | 18c-fa[e] <==> | 0 | 0 | |
| 17 | Glycerol Input | Ex_glyc | | | Exchange | glyc[e] <==> | 0 | 0 | |
| 18 | Triacylglycerol Exchange | Ex_tag | | | Exchange | tag[e] <==> | 0 | 0 | |
| 19 | Diacylglycerol Exchange | Ex_dag | | | Exchange | 12dag[e] <==> | 0 | 0 | |
| 20 | Monacylglycerol Exchange | Ex_mag | | | Exchange | 1mag[e] <==> | 0 | 0 | |
| 21 | Alpha-ketogulterate Exchange | Ex_akg | | | Exchange | akg[e] --> | -999999 | 999999 | |
| 22 | Oxaloacetate Exchange | Ex_oaa | | | Exchange | oaa[e] --> | -999999 | 999999 | |
| 23 | Ribose Nutrient | Ex_rib | | Extracellular | Exchange | rib[e] <==> | 0 | 0 | |
| 24 | Oxygen Input | Nut_o2 | | | Nutrient | o2 <==> | -999999 | 999999 | |
| 25 | Carbon Dioxide Input | Nut_co2 | | | Nutrient | co2 <==> | -999999 | 999999 | |
| 26 | Water Nutrient | Nut_h2o | | | Nutrient | h2o <==> | -999999 | 999999 | |
| 27 | Phosphate Nutrient | Nut_p | | | Nutrient | p <==> | -999999 | 999999 | |
| 28 | Bicarbonate | Nut_hco3 | | | Nutrient | hco3 <==> | -999999 | 999999 | |
| 29 | Coenzyme A | Nut_coa | | | Nutrient | coa[e] <==> | -999999 | 999999 | |
| 30 | Carnitine | Nut_carn | | | Nutrient | carn[e] <==> | 0 | 999999 | |
| 31 | Folate Nutrient | Nut_dhf | | | Nutrient | dhf[c] <==> | -999999 | 999999 | |
| 32 | lipoprotein lipase | LPL | 3.1.1.34 | extracellular mer | Fatty Acid | tag[e] + h2o --> pal[e] + 12dag[e] | 0 | 999999 | |
| 33 | diacylglycerol lipase | DAGL | 3.1.1.- | extracellular mer | Fatty Acid | 12dag[e] + h2o --> 2mag[e] + pal[e] | 0 | 999999 | |
| 34 | triacylglycerol degradation | 12DAG | | extracellular mer | Fatty Acid | 2mag[e] <==> 1mag[e] | 0 | 999999 | |
| 35 | monacylglycerol lipase | MAGL | 3.1.1.23 | extracellular mer | Fatty Acid | 1mag[e] + h2o --> glyc[e] + pal[e] | 0 | 999999 | |
| 36 | Glucose Input | GLUT4 | | | Cellular Uptake | insulin[e] + gluc[e] --> gluc[c] | 0 | 999999 | |
| 37 | Lactate Input | MCT4 | | | Cellular Uptake | lac[e] <==> lac[c] | 0 | 999999 | |
| 38 | Pyruvate Uptake | MCT_pyr | | | Cellular Uptake | pyr[e] --> pyr[c] | 0 | 999999 | |
| 39 | Palmitate Input (hexadecanoate) | FAT-CD36 | | | Cellular Uptake | pal[e] --> pal[c] | 0 | 999999 | |
| 40 | Propanoate Input | FATprop | | | Cellular Uptake | prop[e] <==> prop[c] | 0 | 0 | |
| 41 | Butanoate Input | FATbut | | | Cellular Uptake | but[e] <==> but[c] | 0 | 0 | |
| 42 | Pentanoate Input | FAT5c | | | Cellular Uptake | 5c-fa[e] <==> 5c-fa[c] | 0 | 0 | |
| 43 | Hexanoate Input | FAT6c | | | Cellular Uptake | 6c-fa[e] <==> 6c-fa[c] | 0 | 0 | |
| 44 | Octanoate Input | FAT8c | | | Cellular Uptake | 8c-fa[e] <==> 8c-fa[c] | 0 | 0 | |
| 45 | Nonanoate Input | FAT9c | | | Cellular Uptake | 9c-fa[e] <==> 9c-fa[c] | 0 | 0 | |
| 46 | Decanoate Input | FAT10c | | | Cellular Uptake | 10c-fa[e] <==> 10c-fa[c] | 0 | 0 | |
| 47 | Dodecanoate Input | FAT12c | | | Cellular Uptake | 12c-fa[e] <==> 12c-fa[c] | 0 | 0 | |
| 48 | Tetradecanoate Input | FAT14c | | | Cellular Uptake | 14c-fa[e] <==> 14c-fa[c] | 0 | 0 | |
| 49 | Octadecanoate Input | FAT18c | | | Cellular Uptake | 18c-fa[e] <==> 18c-fa[c] | 0 | 0 | |
| 50 | Glycerol Input | Alc_glyc | | | Cellular Uptake | glyc[e] <==> glyc[c] | 0 | 0 | |
| 51 | Coenzme A Transport | COA | | | Cellular Uptake | coa[e] --> coa[c] | 0 | 999999 | |
| 52 | Solute Carrier family 22 member 5 | SLC22A5-OCTN2 | transport | Outer membranc | Cellular Uptake | carn[e] <==> carn[c] | 0 | 999999 | |
| 53 | Ribose Membrane Transport | Xport_rib | | Membrane | Cellular Uptake | rib[e] --> rib[c] | 0 | 999999 | |
| 54 | alpha-ketogluterate drain | Dr_akg | | | Cellular Export | akg[c] --> akg[e] | 0 | 999999 | |
| 55 | Oxaloacetate drain | Dr_oaa | | | Cellular Export | oaa[c] --> oaa[e] | 0 | 999999 | |
| 56 | Hexokinase | Hex | 2.7.1.2 | Cytosolic | Glycolysis | atp + gluc[c] --> adp + p + g6p[c] | 0 | 999999 | IR |
| 57 | Phosphogluocisomerase | PGI | 5.3.1.9 | Cytosolic | Glycolysis | g6p[c] <==> f6p[c] | -999999 | 999999 | |
| 58 | Phosphofructokinase | PFK | 2.7.1.11 | Cytosolic | Glycolysis | atp + f6p[c] --> adp + fbp[c] | 0 | 999999 | IR |
| 59 | Fructosebisphosphatase | FBP | 3.1.3.11 | Cytosolic | Glycolysis | fbp[c] + h2o --> f6p[c] + p | 0 | 999999 | IR |
| 60 | Aldolase | Ald | 4.1.2.13 | Cytosolic | Glycolysis | fbp[c] <==> dhap[c] + g3p[c] | -999999 | 999999 | |
| 61 | Triose-P-isomerase | TPI | 5.3.1.1 | Cytosolic | Glycolysis | g3p[c] <==> dhap[c] | 0 | 999999 | IR |
| 62 | Glyceraldehyde-3-phosphate dehydrogenase | D3PDeh | 1.2.1.12 | Cytosolic | Glycolysis | g3p[c] + nad[c] <==> dpg[c] + nadh[c] | -999999 | 999999 | |
| 63 | Phosphoglycerate kinase | PGK | 2.7.2.3 | Cytosolic | Glycolysis | dpg[c] + adp <==> 3pg[c] + atp | 0 | 999999 | IR |
| 64 | Phosphoglycerate mutase | PGM | 5.4.2.1 | Cytosolic | Glycolysis | 3pg[c] <==> 2pg[c] | -999999 | 999999 | |
| 65 | Enolase | Eno | 4.2.1.11 | Cytosolic | Glycolysis | 2pg[c] <==> pep[c] + h2o | -999999 | 999999 | |
| 66 | Pyruvate kinase | PyrK | 2.7.1.40 | Cytosolic | Glycolysis | pep[c] + adp --> pyr[c] + atp | 0 | 999999 | IR |
| 67 | PEP synthase | PEPSynth | 2.7.9.2 | Cytosolic | Glycolysis | pyr[c] + atp + h2o --> pep[c] + amp + p | 0 | 999999 | IR |

| 0 | Enzyme | Abrev | EC Number | Location | Pathway | Reaction | Lower Bound | Upper Bound | Thermodynamics |
|---|---|---|---|---|---|---|---|---|---|
| 68 | PEP carboxy kinase | PEPCarbK | 4.1.1.49 | Cytosolic | Glycolysis | oaa[c] + atp --> co2 + pep[c] + adp | 0 | 999999 | IR |
| 69 | PEP carboxylase | PEPCarbl | 4.1.1.31 | Cytosolic | Glycolysis | oaa[c] + p --> pep[c] + h2o + co2 | 0 | 999999 | IR |
| 70 | Lactate dehydrognease | LacDeh | 1.1.2.4 | Cytosolic | Glycolysis | lac[c] + nad[c] <==> pyr[c] + nadh[c] | -999999 | 999999 | |
| 71 | Malatate dehydrogenase (isozyme) | MalDehC | | Cytosolic | Shuttle Cytosolic | oaa[c] + nadh[c] <==> mal[c] + nad[c] | -999999 | 999999 | |
| 72 | Aspartate aminotransferase | ASTC | | Cytosolic | Shuttle Cytosolic | asp[c] + akg[c] <==> glu[c] + oaa[c] | -999999 | 999999 | |
| 73 | Cytosolic glycerol-3-P-dehydrogenase | G3PDehC | 1.1.1.8 | Cytosolic | Shuttle Cytosolic | dhap[c] + nadh[c] <==> glyc3p[c] + nad[c] | -999999 | 999999 | |
| 74 | Malate-alpha-ketoglutarate exchange | Mal-AKGEX | | Mitochondrion T | Shuttle Mitochondrial | mal[c] + akg[m] <==> mal[m] + akg[c] | -999999 | 999999 | |
| 75 | Glutamate-aspartate exchange | Glu-AspEx | | Mitochondrion T | Shuttle Mitochondrial | asp[m] + glu[c] <==> asp[c] + glu[m] | -999999 | 999999 | |
| 76 | Pyruvate Transport (MCT) | MCT | | Mitochondrion T | TCA | pyr[c] --> pyr[m] | -999999 | 999999 | |
| 77 | Aspartate aminotransferase | ASTM | 2.6.1.1 | Mitochondrion | Shuttle Mitochondrial | glu[m] + oaa[m] <==> asp[m] + akg[m] | -999999 | 999999 | |
| 78 | Mitochondrionl glyceral-3-P-dehydrogenase | G3PDehM | 1.1.95.5 | Mitochondrion | Shuttle Mitochondrial | glyc3p[m] + fad[m] <==> dhap[m] + fadh2[m] | -999999 | 999999 | |
| 79 | Pyruvate synthase | PyrSynth | 1.2.4.1 | Mitochondrion | TCA | pyr[m] + coa[m] + nad[m] --> acetyl-coa[m] + nadh[m] + co2 | 0 | 999999 | IR |
| 80 | Citrate synthase | CitSynth | 2.3.3.1 | Mitochondrion | TCA | oaa[m] + acetyl-coa[m] + h2o<==> cit[m] + coa[m] | -999999 | 999999 | R |
| 81 | Aconitase | Acon | 4.2.1.3 | Mitochondrion | TCA | cit[m] <==> iso[m] | -999999 | 999999 | R |
| 82 | Isocitrate dehydrognease | IsoDeh | 1.1.1.41 | Mitochondrion | TCA | iso[m] + nad[m] --> akg[m] + co2 + nadh[m] | 0 | 999999 | IR |
| 83 | Isocitrate dehydrognease | IsoDehnadph | 1.1.1.42 | Mitochondrion | TCA | iso[m] + nadp[m] --> akg[m] + co2 + nadph[m] | 0 | 999999 | IR |
| 84 | alpha-Ketoglutarate dehydrogenase | AKGDeh | 1.2.4.2 | Mitochondrion | TCA | akg[m] + coa[m] + nad[m] --> suc-coa[m] + co2 + nadh[m] | 0 | 999999 | IR |
| 85 | Succinly-CoA synthetase | SunCoaSynth | 6.2.1.5 | Mitochondrion | TCA | suc-coa[m] + adp + p <==> suc[m] + coa[m] + atp | -999999 | 999999 | R |
| 86 | Succinate dehydrogenase (complex II) | SucDeh-II | 1.3.99.1 | Mitochondrion | TCA | suc[m] + fad[m] <==> fum[m] + fadh2[m] | -999999 | 999999 | R |
| 87 | Fumarase | Fum | 4.2.1.2 | Mitochondrion | TCA | fum[m] <==> mal[m] + h2o | -999999 | 999999 | R |
| 88 | Malatate dehydrogenase (isozyme) | MalDehM | 1.1.1.37 | Mitochondrion | TCA | mal[m] + nad[m] <==> oaa[m] + nadh[m] | -999999 | 999999 | R |
| 89 | NADH dehyrdogenase (complex I) | NADHDeh-I | 1.6.5.3 | Mitochondrion | Electron Transport Chain | nadh[m] + uq[m] --> nad[m] + uqh2[m] + 4 h[out] | 0 | 999999 | IR |
| 90 | Electron transfer flavoprotein-ubiquinone oxidoreductase | ETFUO | 1.5.5.1 | Mitchondrion | Electron Transport Chain | fadh2[m] + uq[m] --> fad[m] + uqh2[m] | 0 | 999999 | IR |
| 91 | Ubiquinone cytochrome c-oxidoreductase (complex III) | UCytC-III | 1.10.2.2 | Mitochondrion | Electron Transport Chain | 2 ocytC[m] + uqh2[m] --> 2 rcytC[m] + uq[m] + 4 h[out] | 0 | 999999 | IR |
| 92 | Cytrochrome c-oxidase (complex IV) | CytCO-IV | 1.9.3.1 | Mitochondrion | Electron Transport Chain | o2 + 4 rcytC[m] --> 2 h2o + 4 ocytC[m] + 8 h[out] | 0 | 999999 | IR |
| 93 | ATP synthase (complex V) | ATPSynth-V | 3.6.3.14 | Mitochondrion | Electron Transport Chain | adp + p + 4 h[out]<==> atp + h2o | 0 | 999999 | IR |
| 94 | Convert Amp to Adp | AMP_ADPsupply | | Mitochondrion | Electron Transport Chain | amp + p <==> adp | -999999 | 999999 | R |
| 95 | Palmitate thiokinase | PalTK | 6.2.1.3 | Cytosolic | Mitchondrial Transport | pal[c] + coa[c] + atp --> 234pal-coa[c] + 2 p + amp | 0 | 999999 | |
| 96 | Carnitine palmitoyltransferase mitochondrial muscle isoform | CPT1B-CHKL | 2.3.1.21 | outer mitochond | Mitchondrial Transport | carn[c] + 234pal-coa[c] <==> pal-carn[c] + coa[c] | 0 | 999999 | |
| 97 | Mitochondrial carnitine/acylcarnitine carrier protein | SLC25A20-CACT | transport | | Mitchondrial Transport | pal-carn[c] + carn[m] --> carn[c] + pal-carn[m] | 0 | 9999999 | |
| 98 | | CPT2 | 2.3.1.21 | | Fatty Acid | pal-carn[m] + coa[m] --> 234pal-coa[m] + carn[m] | 0 | 999999 | |
| 99 | Acyl-CoA dehyrdogenase | AcylCoADeh | 1.3.99.3 | Mitochondrion | Fatty Acid | 234pal-coa[m] + fad[m] --> fadh2[m] + 23pal-coa[m] | 0 | 999999 | |
| 100 | Enoyl-CoA hydratase | EnoCoADeh | 4.2.1.17 | Mitochondrion | Fatty Acid | 23pal-coa[m] + h2o <==> 3ohpal-coa[m] | -999999 | 999999 | |
| 101 | 3-Hydroxyacyl-CoA dehydrogenase | 3HA-CoADeh | 1.1.1.35 | Mitochondrion | Fatty Acid | 3ohpal-coa[m] + nad[m] <==> 3ketopal-coa[m] + nadh[m] | -999999 | 999999 | |
| 102 | Acyl-CoA acetyltransferase | AcylCoAAT | 2.3.1.16 | Mitochondrion | Fatty Acid | 3ketopal-coa[m] + coa[m] --> 14c-234acyl-coa[m] + acetyl-coa[m] | 0 | 999999 | |
| 103 | Acyl-CoA dehyrdogenase | AcylCoADeh-18-c | 1.3.99.3 | Mitochondrion | Fatty Acid | 18c-234acyl-coa[m] + fad[m] --> 18c-t2enoyl-coa[m] + fadh2[m] | 0 | 999999 | |
| 104 | Enoyl-CoA hydratase | EnoCoADeh-18c | 4.2.1.17 | Mitochondrion | Fatty Acid | 18c-t2enoyl-coa[m] + h2o <==> 18c-3ohacyl-coa[m] | -999999 | 999999 | |
| 105 | 3-Hydroxyacyl-CoA dehydrogenase | 3HA-CoADeh-18c | 1.1.1.35 | Mitochondrion | Fatty Acid | 18c-3ohacyl-coa[m] + nad[m] <==> 18c-3ketoacyl-coa[m] + nadh[m] | -999999 | 999999 | |
| 106 | Acyl-CoA acetyltransferase | AcylCoAAT-18c | 2.3.1.16 | Mitochondrion | Fatty Acid | 18c-3ketoacyl-coa[m] + coa[m] --> 234pal-coa[m] + acetyl-coa[m] | 0 | 999999 | |
| 107 | Acyl-CoA dehyrdogenase | AcylCoADeh-14-c | 1.3.99.3 | Mitochondrion | Fatty Acid | 14c-234acyl-coa[m] + fad[m] --> 14c-t2enoyl-coa[m] + fadh2[m] | 0 | 999999 | |
| 108 | Enoyl-CoA hydratase | EnoCoADeh-14c | 4.2.1.17 | Mitochondrion | Fatty Acid | 14c-t2enoyl-coa[m] + h2o <==> 14c-3ohacyl-coa[m] | -999999 | 999999 | |
| 109 | 3-Hydroxyacyl-CoA dehydrogenase | 3HA-CoADeh-14c | 1.1.1.35 | Mitochondrion | Fatty Acid | 14c-3ohacyl-coa[m] + nad[m] <==> 14c-3ketoacyl-coa[m] + nadh[m] | -999999 | 999999 | |
| 110 | Acyl-CoA acetyltransferase | AcylCoAAT-14c | 2.3.1.16 | Mitochondrion | Fatty Acid | 14c-3ketoacyl-coa[m] + coa[m] --> 12c-234acyl-coa[m] + acetyl-coa[m] | 0 | 999999 | |
| 111 | Acyl-CoA dehyrdogenase | AcylCoADeh-12-c | 1.3.99.3 | Mitochondrion | Fatty Acid | 12c-234acyl-coa[m] + fad[m] --> 12c-t2enoyl-coa[m] + fadh2[m] | 0 | 999999 | |
| 112 | Enoyl-CoA hydratase | EnoCoADeh-12c | 4.2.1.17 | Mitochondrion | Fatty Acid | 12c-t2enoyl-coa[m] + h2o <==> 12c-3ohacyl-coa[m] | -999999 | 999999 | |
| 113 | 3-Hydroxyacyl-CoA dehydrogenase | 3HA-CoADeh-12c | 1.1.1.35 | Mitochondrion | Fatty Acid | 12c-3ohacyl-coa[m] + nad[m] <==> 12c-3ketoacyl-coa[m] + nadh[m] | -999999 | 999999 | |
| 114 | Acyl-CoA acetyltransferase | AcylCoAAT-12c | 2.3.1.16 | Mitochondrion | Fatty Acid | 12c-3ketoacyl-coa[m] + coa[m] --> 10c-234acyl-coa[m] + acetyl-coa[m] | 0 | 999999 | |
| 115 | Acyl-CoA dehyrdogenase | AcylCoADeh-10-c | 1.3.99.3 | Mitochondrion | Fatty Acid | 10c-234acyl-coa[m] + fad[m] --> 10c-t2enoyl-coa[m] + fadh2[m] | 0 | 999999 | |
| 116 | Enoyl-CoA hydratase | EnoCoADeh-10c | 4.2.1.17 | Mitochondrion | Fatty Acid | 10c-t2enoyl-coa[m] + h2o <==> 10c-3ohacyl-coa[m] | -999999 | 999999 | |
| 117 | 3-Hydroxyacyl-CoA dehydrogenase | 3HA-CoADeh-10c | 1.1.1.35 | Mitochondrion | Fatty Acid | 10c-3ohacyl-coa[m] + nad[m] <==> 10c-3ketoacyl-coa[m] + nadh[m] | -999999 | 999999 | |
| 118 | Acyl-CoA acetyltransferase | AcylCoAAT-10c | 2.3.1.16 | Mitochondrion | Fatty Acid | 10c-3ketoacyl-coa[m] + coa[m] --> 8c-234acyl-coa[m] + acetyl-coa[m] | 0 | 999999 | |
| 119 | Acyl-CoA dehyrdogenase | AcylCoADeh-8-c | 1.3.99.3 | Mitochondrion | Fatty Acid | 8c-234acyl-coa[m] + fad[m] --> 8c-t2enoyl-coa[m] + fadh2[m] | 0 | 999999 | |
| 120 | Enoyl-CoA hydratase | EnoCoADeh-8c | 4.2.1.17 | Mitochondrion | Fatty Acid | 8c-t2enoyl-coa[m] + h2o <==> 8c-3ohacyl-coa[m] | -999999 | 999999 | |
| 121 | 3-Hydroxyacyl-CoA dehydrogenase | 3HA-CoADeh-8c | 1.1.1.35 | Mitochondrion | Fatty Acid | 8c-3ohacyl-coa[m] + nad[m] <==> 8c-3ketoacyl-coa[m] + nadh[m] | -999999 | 999999 | |
| 122 | Acyl-CoA acetyltransferase | AcylCoAAT-8c | 2.3.1.16 | Mitochondrion | Fatty Acid | 8c-3ketoacyl-coa[m] + coa[m] --> 6c-234acyl-coa[m] + acetyl-coa[m] | 0 | 999999 | |
| 123 | Acyl-CoA dehyrdogenase | AcylCoADeh-6-c | 1.3.99.3 | Mitochondrion | Fatty Acid | 6c-234acyl-coa[m] + fad[m] --> 6c-t2enoyl-coa[m] + fadh2[m] | 0 | 999999 | |
| 124 | Enoyl-CoA hydratase | EnoCoADeh-6c | 4.2.1.17 | Mitochondrion | Fatty Acid | 6c-t2enoyl-coa[m] + h2o <==> 6c-3ohacyl-coa[m] | -999999 | 999999 | |
| 125 | 3-Hydroxyacyl-CoA dehydrogenase | 3HA-CoADeh-6c | 1.1.1.35 | Mitochondrion | Fatty Acid | 6c-3ohacyl-coa[m] + nad[m] <==> 6c-3ketoacyl-coa[m] + nadh[m] | -999999 | 999999 | |
| 126 | Acyl-CoA acetyltransferase | AcylCoAAT-6c | 2.3.1.16 | Mitochondrion | Fatty Acid | 6c-3ketoacyl-coa[m] + coa[m] --> butyryl-coa[m] + acetyl-coa[m] | 0 | 999999 | |
| 127 | Butyrl-coa Dehyrogenaze | ButyrlCoADeh | 1.3.99.2 | Mitochondrion | Fatty Acid | butyryl-coa[m] + fad[m] --> tbut2enoyl-coa[m] + fadh2[m] | 0 | 999999 | |
| 128 | Enoyl-CoA hydratase | EnoCoADeh-But | 4.2.1.17 | Mitochondrion | Fatty Acid | tbut2enoyl-coa[m] + h2o <==> 3ohbutyryl-coa[m] | -999999 | 999999 | |
| 129 | 3-Hydroxyacyl-CoA dehydrogenase | 3HA-CoADeh-But | 1.1.1.35 | Mitochondrion | Fatty Acid | 3ohbutyryl-coa[m] + coa[m] <==> acetoacetyl-coa[m] | -999999 | 999999 | |
| 130 | Acyl-CoA acetyltransferase | AcylCoAAT-But | 2.3.1.9 | Mitochondrion | Fatty Acid | acetoacetyl-coa[m] --> 2 acetyl-coa[m] | 0 | 999999 | |
| 131 | Glycerol Kinase | GK | 2.7.1.30 | | TAG | glyc[c] + atp <==> glyc3p[c] + adp | 0 | 999999 | IR |
| 132 | Glycerol-3-phophate acyltransferase 4 | GPAT4 | 2.3.1.15 | | TAG | glyc3p[c] + 234pal-coa[c] <==> 1acyl-g3p[c] + coa[c] | -999999 | 999999 | R |
| 133 | 1-acylglycerol-3-phosphate O-acyltransferase | ABHD5 | 2.3.1.51 | | TAG | 1acyl-g3p[c] + 234pal-coa[c] <==> 12dacyl-g3p[c] + coa[c] | -999999 | 999999 | R |
| 134 | Diacylglycerol Kinase zeta | DAGZ-DAGK6 | 2.7.1.107 | | TAG | 12dacyl-g3p[c] + adp <==> 12dag[c] + atp | -999999 | 999999 | R |

| 0 | Enzyme | Abrev | EC Number | Location | Pathway | Reaction | Lower Bound | Upper Bound | Thermodynamics |
|---|---|---|---|---|---|---|---|---|---|
| 135 | diacylglycerol acyl transferase | DGAT1 | 2.3.1.20 | | TAG | 12dag[c] + 234pal-coa[c] <==> tag[c] + coa[c] | -999999 | 999999 | R |
| 136 | Store TAG | St_TAG | | | TAG | tag[c] --> | 0 | 999999 | IR |
| 137 | Acyl-coaenzyme A synthetase | ACAS2 | 6.2.1.1/6.2.1 | Cellular | Fatty Acid | prop[c] + atp + coa[c] --> prop-coa[c] + adp + p | 0 | 999999 | |
| 138 | Acyl-coaenzyme A synthetase | ACSM-But | 6.2.1.2 | Cellular | Fatty Acid | but[c] + atp + coa[c] --> butyryl-coa[c] + adp + p | 0 | 999999 | |
| 139 | Acyl-coaenzyme A synthetase | ASCM-5c | 6.2.1.2 | Cellular | Fatty Acid | 5c-fa[c] + atp + coa[c] --> 5c-234acyl-coa[c] + adp + p | 0 | 999999 | |
| 140 | Acyl-coaenzyme A synthetase | ASCM-6c | 6.2.1.3 | Cellular | Fatty Acid | 6c-fa[c] + atp + coa[c] --> 6c-234acyl-coa[c] + adp + p | 0 | 999999 | |
| 141 | Acyl-coaenzyme A synthetase | ACSM-8c | 6.2.1.4 | Cellular | Fatty Acid | 8c-fa[c] + atp + coa[c] --> 8c-234acyl-coa[c] + adp + p | 0 | 999999 | |
| 142 | Acyl-coaenzyme A synthetase | ACSM-9c | 6.2.1.5 | Cellular | Fatty Acid | 9c-fa[c] + atp + coa[c] --> 9c-234acyl-coa[c] + adp + p | 0 | 999999 | |
| 143 | Acyl-coaenzyme A synthetase | ACSM-10c | 6.2.1.6 | Cellular | Fatty Acid | 10c-fa[c] + atp + coa[c] --> 10c-234acyl-coa[c] + adp + p | 0 | 999999 | |
| 144 | Acyl-coaenzyme A synthetase | ACSM-12c | 6.2.1.7 | Cellular | Fatty Acid | 12c-fa[c] + atp + coa[c] --> 12c-234acyl-coa[c] + adp + p | 0 | 999999 | |
| 145 | Acyl-coaenzyme A synthetase | ACSM-14c | 6.2.1.8 | Cellular | Fatty Acid | 14c-fa[c] + atp + coa[c] --> 14c-234acyl-coa[c] + adp + p | 0 | 999999 | |
| 146 | Acyl-coaenzyme A synthetase | ACSM-18c | 6.2.1.8 | Cellular | Fatty Acid | 18c-fa[c] + atp + coa[c] --> 18c-234acyl-coa[c] + adp + p | 0 | 999999 | |
| 147 | Carnitine palmitoyltransferase mitochondrial muscle isoform | CPT1B-CHKL | 2.3.1.21 | outer mitochond | Mitchondrial Transport | prop-coa[c] + carn[c] --> prop-carn[c] + coa[c] | 0 | 999999 | |
| 148 | Carnitine palmitoyltransferase mitochondrial muscle isoform | CPT1B-CHKL | 2.3.1.21 | outer mitochond | Mitchondrial Transport | butyryl-coa[c] + carn[c] --> butyryl-carn[c] + coa[c] | 0 | 999999 | |
| 149 | Carnitine palmitoyltransferase mitochondrial muscle isoform | CPT1B-CHKL | 2.3.1.21 | outer mitochond | Mitchondrial Transport | 5c-234acyl-coa[c] + carn[c] --> 5c-234acyl-carn[c] + coa[c] | 0 | 999999 | |
| 150 | Carnitine palmitoyltransferase mitochondrial muscle isoform | CPT1B-CHKL | 2.3.1.21 | outer mitochond | Mitchondrial Transport | 6c-234acyl-coa[c] + carn[c] --> 6c-234acyl-carn[c] + coa[c] | 0 | 999999 | |
| 151 | Carnitine palmitoyltransferase mitochondrial muscle isoform | CPT1B-CHKL | 2.3.1.21 | outer mitochond | Mitchondrial Transport | 8c-234acyl-coa[c] + carn[c] --> 8c-234acyl-carn[c] + coa[c] | 0 | 999999 | |
| 152 | Carnitine palmitoyltransferase mitochondrial muscle isoform | CPT1B-CHKL | 2.3.1.21 | outer mitochond | Mitchondrial Transport | 9c-234acyl-coa[c] + carn[c] --> 9c-234acyl-carn[c] + coa[c] | 0 | 999999 | |
| 153 | Carnitine palmitoyltransferase mitochondrial muscle isoform | CPT1B-CHKL | 2.3.1.21 | outer mitochond | Mitchondrial Transport | 10c-234acyl-coa[c] + carn[c] --> 10c-234acyl-carn[c] + coa[c] | 0 | 999999 | |
| 154 | Carnitine palmitoyltransferase mitochondrial muscle isoform | CPT1B-CHKL | 2.3.1.21 | outer mitochond | Mitchondrial Transport | 12c-234acyl-coa[c] + carn[c] --> 12c-234acyl-carn[c] + coa[c] | 0 | 999999 | |
| 155 | Carnitine palmitoyltransferase mitochondrial muscle isoform | CPT1B-CHKL | 2.3.1.21 | outer mitochond | Mitchondrial Transport | 14c-234acyl-coa[c] + carn[c] --> 14c-234acyl-carn[c] + coa[c | 0 | 999999 | |
| 156 | Carnitine palmitoyltransferase mitochondrial muscle isoform | CPT1B-CHKL | 2.3.1.21 | outer mitochond | Mitchondrial Transport | 18c-234acyl-coa[c] + carn[c] --> 18c-234acyl-carn[c] + coa[c | 0 | 999999 | |
| 157 | Mitochondrial carnitine/acylcarnitine carrier protein | SLC25A20-CACT | transport | Mitochondrial M | Mitchondrial Transport | prop-carn[c] + carn[m] --> prop-carn[m] + carn[c] | 0 | 999999 | |
| 158 | Mitochondrial carnitine/acylcarnitine carrier protein | SLC25A20-CACT | transport | Mitochondrial M | Mitchondrial Transport | butyryl-carn[c] + carn[m] --> butyryl-carn[m] + carn[c] | 0 | 999999 | |
| 159 | Mitochondrial carnitine/acylcarnitine carrier protein | SLC25A20-CACT | transport | Mitochondrial M | Mitchondrial Transport | 5c-234acyl-carn[c] + carn[m] --> 5c-234acyl-carn[m] + carn[c] | 0 | 999999 | |
| 160 | Mitochondrial carnitine/acylcarnitine carrier protein | SLC25A20-CACT | transport | Mitochondrial M | Mitchondrial Transport | 6c-234acyl-carn[c] + carn[m] --> 6c-234acyl-carn[m] + carn[c] | 0 | 999999 | |
| 161 | Mitochondrial carnitine/acylcarnitine carrier protein | SLC25A20-CACT | transport | Mitochondrial M | Mitchondrial Transport | 8c-234acyl-carn[c] + carn[m] --> 8c-234acyl-carn[m] + carn[c] | 0 | 999999 | |
| 162 | Mitochondrial carnitine/acylcarnitine carrier protein | SLC25A20-CACT | transport | Mitochondrial M | Mitchondrial Transport | 9c-234acyl-carn[c] + carn[m] --> 9c-234acyl-carn[m] + carn[c] | 0 | 999999 | |
| 163 | Mitochondrial carnitine/acylcarnitine carrier protein | SLC25A20-CACT | transport | Mitochondrial M | Mitchondrial Transport | 10c-234acyl-carn[c] + carn[m] --> 10c-234acyl-carn[m] + carn[c] | 0 | 999999 | |
| 164 | Mitochondrial carnitine/acylcarnitine carrier protein | SLC25A20-CACT | transport | Mitochondrial M | Mitchondrial Transport | 12c-234acyl-carn[c] + carn[m] --> 12c-234acyl-carn[m] + carn[c] | 0 | 999999 | |
| 165 | Mitochondrial carnitine/acylcarnitine carrier protein | SLC25A20-CACT | transport | Mitochondrial M | Mitchondrial Transport | 14c-234acyl-carn[c] + carn[m] --> 14c-234acyl-carn[m] + carn[c] | 0 | 999999 | |
| 166 | Mitochondrial carnitine/acylcarnitine carrier protein | SLC25A20-CACT | transport | Mitochondrial M | Mitchondrial Transport | 18c-234acyl-carn[c] + carn[m] --> 18c-234acyl-carn[m] + carn[c] | 0 | 999999 | |
| 167 | | CPT2 | 2.3.1.21 | | Mitchondrial Transport | prop-carn[m] + coa[m] --> prop-coa[m] + carn[m] | 0 | 999999 | |
| 168 | | CPT2 | 2.3.1.21 | | Mitchondrial Transport | butyryl-carn[m] + coa[m] --> butyryl-coa[m] + carn[m] | 0 | 999999 | |
| 169 | | CPT2 | 2.3.1.21 | | Mitchondrial Transport | 5c-234acyl-carn[m] + coa[m] --> 5c-234acyl-coa[m] + carn[m] | 0 | 999999 | |
| 170 | | CPT2 | 2.3.1.21 | | Mitchondrial Transport | 6c-234acyl-carn[m] + coa[m] --> 6c-234acyl-coa[m] + carn[m] | 0 | 999999 | |
| 171 | | CPT2 | 2.3.1.21 | | Mitchondrial Transport | 8c-234acyl-carn[m] + coa[m] --> 8c-234acyl-coa[m] + carn[m] | 0 | 999999 | |
| 172 | | CPT2 | 2.3.1.21 | | Mitchondrial Transport | 9c-234acyl-carn[m] + coa[m] --> 9c-234acyl-coa[m] + carn[m] | 0 | 999999 | |
| 173 | | CPT2 | 2.3.1.21 | | Mitchondrial Transport | 10c-234acyl-carn[m] + coa[m] --> 10c-234acyl-coa[m] + carn[m] | 0 | 999999 | |
| 174 | | CPT2 | 2.3.1.21 | | Mitchondrial Transport | 12c-234acyl-carn[m] + coa[m] --> 12c-234acyl-coa[m] + carn[m] | 0 | 999999 | |
| 175 | | CPT2 | 2.3.1.21 | | Mitchondrial Transport | 14c-234acyl-carn[m] + coa[m] --> 14c-234acyl-coa[m] + carn[m] | 0 | 999999 | |
| 176 | | CPT2 | 2.3.1.21 | | Mitchondrial Transport | 18c-234acyl-carn[m] + coa[m] --> 18c-234acyl-coa[m] + carn[m] | 0 | 999999 | |
| 177 | Acyl-CoA dehyrdogenase | AcylCoADeh-9-c | 1.3.99.3 | Mitochondrion | Fatty Acid | 9c-234acyl-coa[m] + fad[m] --> 9c-t2enoyl-coa[m] + fadh2[m] | 0 | 999999 | |
| 178 | Enoyl-CoA hydratase | EnoCoADeh-9c | 4.2.1.17 | Mitochondrion | Fatty Acid | 9c-t2enoyl-coa[m] + h2o <==> 9c-3ohacyl-coa[m] | -999999 | 999999 | |
| 179 | 3-Hydroxyacyl-CoA dehydrogenase | 3HA-CoADeh-9c | 1.1.1.35 | Mitochondrion | Fatty Acid | 9c-3ohacyl-coa[m] + nad[m] <==> 9c-3ketoacyl-coa[m] + nadh[m] | -999999 | 999999 | |
| 180 | Acyl-CoA acetyltransferase | AcylCoAAT-9c | 2.3.1.16 | Mitochondrion | Fatty Acid | 9c-3ketoacyl-coa[m] + coa[m] --> 7c-234acyl-coa[m] + acetyl-coa[m] | 0 | 999999 | |
| 181 | Acyl-CoA dehyrdogenase | AcylCoADeh-7-c | 1.3.99.3 | Mitochondrion | Fatty Acid | 7c-234acyl-coa[m] + fad[m] --> 7c-t2enoyl-coa[m] + fadh2[m] | 0 | 999999 | |
| 182 | Enoyl-CoA hydratase | EnoCoADeh-7c | 4.2.1.17 | Mitochondrion | Fatty Acid | 7c-t2enoyl-coa[m] + h2o <==> 7c-3ohacyl-coa[m] | -999999 | 999999 | |
| 183 | 3-Hydroxyacyl-CoA dehydrogenase | 3HA-CoADeh-7c | 1.1.1.35 | Mitochondrion | Fatty Acid | 7c-3ohacyl-coa[m] + nad[m] <==> 7c-3ketoacyl-coa[m] + nadh[m] | -999999 | 999999 | |
| 184 | Acyl-CoA acetyltransferase | AcylCoAAT-7c | 2.3.1.16 | Mitochondrion | Fatty Acid | 7c-3ketoacyl-coa[m] + coa[m] --> 5c-234acyl-coa[m] + acetyl-coa[m] | 0 | 999999 | |
| 185 | Acyl-CoA dehyrdogenase | AcylCoADeh-5-c | 1.3.99.3 | Mitochondrion | Fatty Acid | 5c-234acyl-coa[m] + fad[m] --> 5c-t2enoyl-coa[m] + fadh2[m] | 0 | 999999 | |
| 186 | Enoyl-CoA hydratase | EnoCoADeh-5c | 4.2.1.17 | Mitochondrion | Fatty Acid | 5c-t2enoyl-coa[m] + h2o <==> 5c-3ohacyl-coa[m] | -999999 | 999999 | |
| 187 | 3-Hydroxyacyl-CoA dehydrogenase | 3HA-CoADeh-5c | 1.1.1.35 | Mitochondrion | Fatty Acid | 5c-3ohacyl-coa[m] + nad[m] <==> 5c-3ketoacyl-coa[m] + nadh[m] | -999999 | 999999 | |
| 188 | Acyl-CoA acetyltransferase | AcylCoAAT-5c | 2.3.1.16 | Mitochondrion | Fatty Acid | 5c-3ketoacyl-coa[m] + coa[m] --> acetyl-coa[m] + prop-coa[m] | 0 | 999999 | |
| 189 | Alcohol Dehydrogenase (NADP) | AKR1A1 | 1.1.1.2 | Cytosolic | Fatty Alcohol | glyc[c] + nadp[c] --> glyc-ald[c] + nadph[c] | 0 | 999999 | |
| 190 | Fatty aldehyde dehydrogenase | ALDH3A2 | 1.2.1.3 | Cytosolic | Fatty Alcohol | glyc-ald[c] + nad[c] + h2o --> glyc-ate[c] + nadh[c] | 0 | 999999 | |
| 191 | Glycerate Kinase | GLYCTK | 2.7.1.31 | Cytosolic | Fatty Alcohol | glyc-ate[c] + atp --> 3pg[c] + adp + p | 0 | 999999 | |
| 192 | | EAA_arg | | | Exchange | arg[c] <==> | -999999 | 0 | |
| 193 | | EAA_his | | | Exchange | his[c] <==> | -999999 | 0 | |
| 194 | | EAA_ile | | | Exchange | ile[c] <==> | -999999 | 0 | |
| 195 | | EAA_leu | | | Exchange | leu[c] <==> | -999999 | 0 | |
| 196 | | EAA_lys | | | Exchange | lys[c] <==> | -999999 | 0 | |
| 197 | | EAA_met | | | Exchange | met[c] <==> | -999999 | 0 | |
| 198 | | EAA_phe | | | Exchange | phe[c] <==> | -999999 | 0 | |
| 199 | | EAA_thr | | | Exchange | thr[c] <==> | -999999 | 0 | |
| 200 | | EAA_trp | | | Exchange | trp[c] <==> | -999999 | 0 | |
| 201 | | EAA_val | | | Exchange | val[c] <==> | -999999 | 0 | |

| 0 | Enzyme | Abrev | EC Number | Location | Pathway | Reaction | Lower Bound | Upper Bound | Thermodynamics |
|---|---|---|---|---|---|---|---|---|---|
| 202 | | NEAA_ala | | | Exchange | ala[c] <==> | -999999 | 0 | |
| 203 | | NEAA_asn | | | Exchange | asn[c] <==> | -999999 | 0 | |
| 204 | | NEAA_asp | | | Exchange | asp[c] <==> | -999999 | 0 | |
| 205 | | NEAA_cys | | | Exchange | cys[c] <==> | -999999 | 0 | |
| 206 | | NEAA_glu | | | Exchange | glu[c] <==> | -999999 | 0 | |
| 207 | | NEAA_gln | | | Exchange | gln[c] <==> | -999999 | 0 | |
| 208 | | NEAA_gly | | | Exchange | gly[c] <==> | -999999 | 0 | |
| 209 | | NEAA_pro | | | Exchange | pro[c] <==> | -999999 | 0 | |
| 210 | | NEAA_ser | | | Exchange | ser[c] <==> | -999999 | 0 | |
| 211 | | NEAA_tyr | | | Exchange | tyr[c] <==> | -999999 | 0 | |
| 212 | Dihydrolipoyl dehydrogenase, mitochondrial | DLDH | 1.8.1.4 | Mitochondrion | Amino Acid Catabolism | laN6dhpl[m] + nad[m] <==> laN6ll[m] + nadh[m] | -999999 | 999999 | |
| 213 | Valine mitochondrial transport | MT_val | | | Mitchondrial Transport | val[c] --> val[m] | 0 | 999999 | |
| 214 | Branched-chain amino acid aminotransferase, mitochondrial prec | BCAT2-val | 2.6.1.42 | Mitochondrion | Amino Acid Catabolism | val[m] + akg[m] --> akisov[m] + glu[m] | 0 | 999999 | |
| 215 | Oxoisovalerate dhydrogenase | BCKDHB-val | 1.2.4.4 | Mitochondrion | Amino Acid Catabolism | akisov[m] + laN6ll[m] --> laN6S2mpdhl[m] + co2 | 0 | 999999 | |
| 216 | Lipoamide acyltransferase component of branched-chain alpha-ke | BCKADE2-val | 2.3.1.168 | Mitochondrion | Amino Acid Catabolism | laN6S2mpdhl[m] + coa[m] --> laN6dhpl[m] + isob-coa[m] | 0 | 999999 | |
| 217 | Acyl-coa dehydrogenase, mitochondrial precursor | ACADSB | 1.3.99.12 | Mitochondrion | Amino Acid Catabolism | isob-coa[m] + fad[m] --> methacrl-coa[m] + fadh2[m] | 0 | 999999 | |
| 218 | Enoyl-coa hydratase, mitochondrial precursor | ECHS1 | 4.2.1.17 | Mitochondrion | Amino Acid Catabolism | methacrl-coa[m] + h2o --> 3hisob-coa[m] | 0 | 999999 | |
| 219 | 3-hydroxyiosbutyryl-coenzyme A hydrolase | HIBCH | 3.1.2.4 | Mitochondrion | Amino Acid Catabolism | 3hisob-coa[m] + h2o --> 3hisob[m] + coa[m] | 0 | 999999 | |
| 220 | 3-hydroxyisobutyrate dehydrogenase, mitochondrial percursor | HIBADH | 1.1.1.31 | Mitochondrion | Amino Acid Catabolism | 3hisob[m] + nad[m] --> methmalsemald[m] + nadh[m] | 0 | 999999 | |
| 221 | Methylmalonate-semialdehyde dehyrdogenase (acylating), mitoch | ALDH6A1 | 1.2.1.27 | Mitochondrion | Amino Acid Catabolism | methmalsemald[m] + nad[m] + coa[m] + h2o --> prop-coa[m] + hco3 + nadh[ | 0 | 999999 | |
| 222 | Propionyl-coa carboxylase | PCCB | 6.4.1.3 | Mitochondrion | Amino Acid Catabolism | prop-coa[m] + atp + hco3 --> Smethmal-coa[m] + adp + p | 0 | 999999 | |
| 223 | Methylmalonyl-coa epimerase | MCEE | 5.1.99.1 | Mitochondrion | Amino Acid Catabolism | Smethmal-coa[m] --> Rmethmal-coa[m] | 0 | 999999 | |
| 224 | Methylmalonyl-coa mutase, mitochondrial percursor | MUT | 5.4.99.2 | Mitochondrion | Amino Acid Catabolism | Rmethmal-coa[m] --> suc-coa[m] | 0 | 999999 | |
| 225 | Isoleucine mitochondrial transport | MT_ile | | | Mitchondrial Transport | ile[c] --> ile[m] | 0 | 999999 | |
| 226 | Branched-chain amino acid aminotransferase, mitochondroal prec | BCAT-ile | 2.3.1.42 | Mitochondrion | Amino Acid Catabolism | ile[m] + akg[m] --> ak3mvalt[m] + glu[m] | 0 | 999999 | |
| 227 | Oxoisovalerate dehydrogenase | BCKDHB-ile | 1.2.4.4 | Mitochondrion | Amino Acid Catabolism | ak3mvalt[m] + laN6ll[m] --> eN6S2mbdhpl[m] + co2 | 0 | 999999 | |
| 228 | Lipoamide acyltransferase component of branched-chain alpha-ke | BCKADE2-ile | 2.3.1.168 | Mitochondrion | Amino Acid Catabolism | eN6S2mbdhpl[m] + coa[m] --> laN6dhpl[m] + t2metbut2en-coa[m] | 0 | 999999 | |
| 229 | Enoyl Coa hydratase | ECHS1 | 4.2.1.17 | Mitochondrion | Amino Acid Catabolism | t2metbut2en-coa[m] + h2o --> 3oh2metbut-coa[m] | 0 | 999999 | |
| 230 | Hydroxysteroid dehydrogenase 10 | HSD17B10_ | 1.1.1.178 | Mitochondrion | Amino Acid Catabolism | 3oh2metbut-coa[m] + nad[m] --> 2met-acetoacetyl-coa[m] + nadh[m] | 0 | 999999 | |
| 231 | Acetyl-coa acetyltransferase, mitochondrial | ACAT1 | 2.3.1.9 | Mitochondrion | Amino Acid Catabolism | 2met-acetoacetyl-coa[m] + coa[m] --> prop-coa[m] + acetyl-coa[m] | 0 | 999999 | |
| 232 | leusine mitochondrial transport | MT_leu | | | Mitchondrial Transport | leu[c] --> leu[m] | 0 | 999999 | |
| 233 | Branched-chain amino acid aminotransferase, mitochondrial prec | BCAT2-leu | 2.6.1.42 | Mitochondrion | Amino Acid Catabolism | leu[m] + akg[m] --> akiso[m] + glu[m] | 0 | 999999 | |
| 234 | Oxoisovalerate dehydrogenase | BCKDHB-leu | 1.2.4.4 | Mitochondrion | Amino Acid Catabolism | akiso[m] + laN6ll[m] --> eN6S3mbdhpl[m] + co2 | 0 | 999999 | |
| 235 | Lipoamide acyltransferase component of branched-chain alpha-ke | BCKADE2-leu | 2.3.1.168 | Mitochondrion | Amino Acid Catabolism | eN6S3mbdhpl[m] + coa[m] --> laN6dhpl[m] + isov-coa[m] | 0 | 999999 | |
| 236 | Isovaleryl-coa dehydrogenase, mitochondrial precursor | IVD | 1.3.99.10 | Mitochondrion | Amino Acid Catabolism | isov-coa[m] + fad[m] --> 3mc-coa[m] + fadh2[m] | 0 | 999999 | |
| 237 | Methylcrotonyl-coa carboxylase | MCC1-MCC2 | 6.4.1.4 | Mitochondrion | Amino Acid Catabolism | 3mc-coa[m] + atp + hco3 --> 3mg-coa[m] + adp + p | 0 | 999999 | |
| 238 | Methylglutaconyl-CoA hydratase, mitochondrial | MGCA | 4.2.1.18 | Mitochondrion | Amino Acid Catabolism | 3mg-coa[m] + h2o --> 3h3mg-coa[m] | 0 | 999999 | |
| 239 | hyroxymethylglutaryl-coa lyase, mitochondrial precursor | HMGCL | 4.1.3.4 | Mitochondrion | Amino Acid Catabolism | 3h3mg-coa[m] --> acetacet[m] + acetyl-coa[m] | 0 | 999999 | |
| 240 | Succinyl-coa: 3-ketoacid-coanezyme A transferase, mitochondrial | OXCT | 2.8.3.5 | Mitochondrion | Amino Acid Catabolism | acetacet[m] + suc-coa[m] --> suc[m] + acetoacetyl-coa[m] | 0 | 999999 | |
| 241 | Hydroxymethylglutaryl-coa synthase, mitochondrial percursor | HMGCS2 | 2.3.3.10 | Mitochondrion | Amino Acid Catabolism | acetoacetyl-coa[m] + acetyl-coa[m] + h2o --> 3h3mg-coa[m] + coa[m] | 0 | 999999 | |
| 242 | Glutamate Dehydrogenase | GluDeh | 1.4.1.3 | Mitochondrion | Amino Acid Catabolism | glu[m] + nadp[m] + h2o <==> akg[m] + nadph[m] + nh3 | -999999 | 999999 | R |
| 243 | Glutamine Synthetase | GlnSynth | 6.3.1.2 | Cytosolic | Amino Acid Biosynthesis | nh3 + glu[c] + atp --> gln[c] + adp + p | 0 | 999999 | IR |
| 244 | Glutamin (nitrogen) elimination in muscle | GLN_Elim | | Cytosolic | Cellular Export | gln[c] --> | 0 | 99999 | IR |
| 245 | Alanine amino transferase 2 | ALT | 2.6.1.2 | Cytosolic | Amino Acid Biosynthesis | glu[c] + pyr[c] <==> akg[c] + ala[c] | -999999 | 999999 | R |
| 246 | Alanine nitrogen elimination in muscle | ALA_Elim | | Cytosolic | Cellular Export | ala[c] --> | 0 | 999999 | IR |
| 247 | Serine Dehydratase | SDS | 4.3.1.17 | Cytosolic | Amino Acid Catabolism | ser[c] --> pyr[c] + nh3 | 0 | 999999 | |
| 248 | Cystathionine beta-synthase | CBS | 4.2.1.22 | Cytosolic | Amino Acid Catabolism | ser[c] + hcys[c] --> cyst[c] + h2o | 0 | 999999 | |
| 249 | Cystathionine gamma-lyase | CTH | 4.4.1.1 | Cytosolic | Amino Acid Catabolism | cyst[c] + h2o --> nh3 + cys[c] + 2ob[c] | 0 | 999999 | |
| 250 | ILVB | ILVBL | 2.2.1.6 | Cytosolic | Amino Acid Catabolism | pyr[c] + 2ob[c] --> 2a2hbut[c] + co2 | 0 | 999999 | |
| 251 | 2-aceto-2-hydroxybutyrate Leak | 2a2hButLeak | | | Cellular Export | 2a2hbut[c] --> | 0 | 999999 | |
| 252 | Lysine mitochondrial trasnport | MT_lys | | | Amino Acid Mit Transport | lys[c] --> lys[m] | 0 | 999999 | |
| 253 | Lysine-ketoglutarate reductase/saccharopine dehydrogenase | AASS | 1.5.1.7 | Mitochondrion | Amino Acid Catabolism | lys[m] + akg[m] + nadh[m] <==> sacpn[m] + nad[m] + h2o | 0 | 999999 | |
| 254 | Lysine-ketoglutarate reductase/saccharopine dehydrogenase | AASS | 1.5.1.9 | Mitochondrion | Amino Acid Catabolism | sacpn[m] + nad[m] + h2o <==> 2aap6semald[m] + glu[m] + nadh[m] | 0 | 999999 | |
| 255 | | aAASADeh | 1.2.1.31 | Mitochondrion | Amino Acid Catabolism | 2aap6semald[m] + nad[m] + h2o <==> aaap[m] + nadh[m] | 0 | 999999 | |
| 256 | L-kynurenine/alpha-aminoadipate aminotransferase | KATII | 2.6.1.39 | Mitochondrion | Amino Acid Catabolism | aaap[m] + akg[m] <==> glu[m] + akap[m] | 0 | 999999 | |
| 257 | | lysdeg | | Mitochondrion | Amino Acid Catabolism | akap[m] + nad[m] + coa[m] <==> glut-coa[m] + nadh[m] + co2 | 0 | 999999 | |
| 258 | Glutaryl-coa Dehydrodgenase, mitochondrial precursor | IVD | 1.3.99.7 | Mitochondrion | Amino Acid Catabolism | glut-coa[m] + fad[m] <==> tbut2enoyl-coa[m] + fadh2[m] | 0 | 999999 | |
| 259 | | MT_pro | | | Amino Acid Mit Transport | pro[c] --> pro[m] | 0 | 999999 | |
| 260 | Poline oxidase, mitochondrial precursor | PRODH | 1.5.99.8 | Mitochondrion | Amino Acid Catabolism | pro[m] + fad[m] --> 1pyrr5carb[m] + fadh2[m] | 0 | 999999 | |
| 261 | Aldehyde dehydrogenase 4A1 percursor | ALDH4a1 | 1.5.1.12 | Mitochondrion | Amino Acid Catabolism | 1pyrr5carb[m] + nad[m] + 2 h2o --> glu[m] + nadh[m] | 0 | 999999 | IR |
| 262 | glycine amidinotransferase, Mitochondrial Precursor | GATM | 2.1.4.1 | Mitochondrion | Amino Acid Catabolism | gly[c] + arg[c] --> guan[c] + orn[m] | 0 | 999999 | committed step |
| 263 | ornithine leak | Dr_orn | | | Cellular Export | orn[m] --> | 0 | 999999 | |
| 264 | Guanidinoacetate N-methyltransferase | GAMT | 2.1.1.2 | Cytosolic | Amino Acid Catabolism | guan[c] + sal-met[c] --> cr + sal-hcys[c] | 0 | 999999 | |
| 265 | | Dr_cr | | | Cellular Export | cr --> | 0 | 999999 | |
| 266 | Adenosylhomocysteinase | AdoHcyase | 3.3.1.1 | | Amino Acid Biosynthesis | sal-hcys[c] + h2o --> hcys[c] + amp | 0 | 999999 | |
| 267 | 5-methyltetrahydrofolate-homcysteine-methyltransferase | MTR | 2.1.1.13 | Cytosolic | Amino Acid Biosynthesis | hcys[c] + 5meth-thf[c] <==> met[c] + thf[c] | -999999 | 999999 | |
| 268 | S-adenosylmethionine synthetase | AdoMet | 2.5.1.6 | | Amino Acid Biosynthesis | atp + met[c] + h2o --> sal-met[c] + 2 p | 0 | 999999 | |

| 0 | Enzyme | Abrev | EC Number | Location | Pathway | Reaction | Lower Bound | Upper Bound | Thermodynamics |
|---|---|---|---|---|---|---|---|---|---|
| 269 | Serine hydroxymethyltransferase | SHMT | 2.1.2.1 | Cytosolic | Amino Acid Biosynthesis | ser[c] + thf[c] <==> gly[c] + 5methyln-thf[c] | -999999 | 999999 | R |
| 270 | Methylenetetrahydrofolate reductase | MTHFR | 1.5.1.20 | Cytosolic | Vitamin Metabolism | 5methyln-thf[c] + nadph[c] --> 5meth-thf[c] + nadp[c] | 0 | 999999 | |
| 271 | Dihydrofolate Reductase | DHFR | 1.5.1.3 | Cytosolic | Vitamin Metabolism | dhf[c] + nadph[c] --> thf[c] + nadp[c] | 0 | 999999 | IR |
| 272 | Histidine Ammonia-lyase | HAL | 4.3.1.3 | Cytosolic | Amino Acid Catabolism | his[c] --> uro[c] + nh3 | 0 | 999999 | IR |
| 273 | Probable urocanate hydratase | FLJ31300 | 4.2.1.49 | Cytosolic | Amino Acid Catabolism | uro[c] + h2o --> 4im5prop[c] | 0 | 999999 | IR |
| 274 | Unknown | MGC35366 | 3.5.2.7 | Cytosolic | Amino Acid Catabolism | 4im5prop[c] + h2o --> form-glu[c] | 0 | 999999 | IR |
| 275 | Formimidoyltransferase-cyclodeaminase, pt 1 | FTCD1 | 2.1.2.5 | Cytosolic | Amino Acid Catabolism | form-glu[c] + thf[c] --> glu[c] + form-thf[c] | 0 | 999999 | IR |
| 276 | Formimidoyltransferase-cyclodeaminase, pt 2 | FTCD2 | 4.3.1.4 | Cytosolic | Vitamin Metabolism | form-thf[c] <==> 5methn-thf[c] + nh3 | -999999 | 999999 | R |
| 277 | Folate Transformation | FT | 1.5.1.15 | Cytosolic | Vitamin Metabolism | 5methn-thf[c] + nadh[c] <==> 5methyln-thf[c] + nad[c] | -999999 | 999999 | R |
| 278 | Asparaginase (asparagine amidohydrolase) | ASNase | 3.5.1.1 | Cytosolic | Amino Acid Biosynthesis | asn[c] + h2o --> asp[c] + nh3 | 0 | 999999 | IR |
| 279 | Asparagine synthetase (glutamine-hydrolyzing) | ASNSynth | 6.3.5.4 | Cytosolic | Amino Acid Biosynthesis | gln[c] + asp[c] + atp + h2o --> glu[c] + asn[c] + amp + 2 p | 0 | 999999 | R |
| 280 | Phosphoglycerate dehydrogenase | PHGDH | 1.1.1.95 | Cytosolic | Amino Acid Biosynthesis | 3pg[c] + nad[c] --> 3phdpyr[c] + nadh[c] | 0 | 999999 | IR |
| 281 | Phosphoserine aminotransferase | PSA | 2.6.1.52 | Cytosolic | Amino Acid Biosynthesis | 3phdpyr[c] + glu[c] --> 3pser[c] + akg[c] | 0 | 999999 | IR |
| 282 | Phosphoserine phosphatase | PSPH | 3.1.3.3 | Cytosolic | Amino Acid Biosynthesis | 3pser[c] + h2o --> ser[c] + p | 0 | 999999 | IR |
| 283 | Glutamate 5-kinase | P5CS | 2.7.2.11 | Mitochondrion | Amino Acid Biosynthesis | glu[m] + atp --> glu-5-p[m] + adp + p | 0 | 999999 | |
| 284 | Gamma-glutamyl phosphate reductase | PYCS | 1.2.1.41 | Mitochondrion | Amino Acid Biosynthesis | glu-5-p[m] + nadph[m] --> glu-5-semald[m] + nadp[m] | 0 | 999999 | |
| 285 | Spontaneous | Spont_Glu | | Mitochondrion | Amino Acid Biosynthesis | glu-5-semald[m] --> 1pyrr5carb[m] + h2o | 0 | 999999 | |
| 286 | Pyrroline 5-carboxylate reductase | PYRCR1 | 1.5.1.2 | Mitochondrion | Amino Acid Biosynthesis | 1pyrr5carb[m] + nadph[m] --> pro[m] + nadp[m] | 0 | 999999 | IR |
| 287 | Phenylalanine-4-hydroxylase | PAG | 1.14.16.1 | Cytosolic | Amino Acid Biosynthesis | phe[c] + thb[c] --> tyr[c] + 4ah-thb[c] | 0 | 999999 | IR |
| 288 | Pterin-4-alpha-carinolamine dehydratase | PCBD | 4.2.1.96 | Cytosolic | Amino Acid Biosynthesis | 4ah-thb[c] --> dhb[c] + h2o | 0 | 999999 | IR |
| 289 | Dihydopterin Reductase | QDPR | 1.5.1.34 | Cytosolic | Amino Acid Biosynthesis | dhb[c] + nadh[c] --> thb[c] + nad[c] | 0 | 999999 | IR |
| 290 | | MT_glu | | | Mitchondrial Transport | glu[m] <==> glu[c] | -999999 | 999999 | |
| 291 | | tRNA | | | Nutrient | trna[c] <==> | -999999 | 999999 | R |
| 292 | Tyrosyl-tRNA synthetase | YARS | 6.1.1.1 | | Translation | trna[c] + tyr[c] + atp <==> trna-tyr[c] + amp + 2 p | -999999 | 999999 | R |
| 293 | Tryptophanyl-tRNA synthetase | WARS | 6.1.1.2 | | Translation | trna[c] + trp[c] + atp <==> trna-trp[c] + amp + 2 p | -999999 | 999999 | R |
| 294 | Threonyl-tRNA Syntetase | TARS | 6.1.1.3 | | Translation | trna[c] + thr[c] + atp <==> trna-thr[c] + amp + 2 p | -999999 | 999999 | R |
| 295 | Leucyl-tRNA Syntetase | LARS | 6.1.1.4 | | Translation | trna[c] + leu[c] + atp <==> trna-leu[c] + amp + 2 p | -999999 | 999999 | R |
| 296 | Isoleucyl-tRNA Syntetase | IARS | 6.1.1.5 | | Translation | trna[c] + ile[c] + atp <==> trna-ile[c] + amp + 2 p | -999999 | 999999 | R |
| 297 | Lysyl-tRNA Syntetase | KARS | 6.1.1.6 | | Translation | trna[c] + lys[c] + atp <==> trna-lys[c] + amp + 2 p | -999999 | 999999 | R |
| 298 | Alanyl-tRNA Syntetase | AARS | 6.1.1.7 | | Translation | trna[c] + ala[c] + atp <==> trna-ala[c] + amp + 2 p | -999999 | 999999 | R |
| 299 | Valyl-tRNA Syntetase | VARS | 6.1.1.9 | | Translation | trna[c] + val[c] + atp <==> trna-val[c] + amp + 2 p | -999999 | 999999 | R |
| 300 | Methionyl-tRNA Syntetase | MARS | 6.1.1.10 | | Translation | trna[c] + met[c] + atp <==> trna-met[c] + amp + 2 p | -999999 | 999999 | R |
| 301 | Seryl-tRNA Syntetase | SARS | 6.1.1.11 | | Translation | trna[c] + ser[c] + atp <==> trna-ser[c] + amp + 2 p | -999999 | 999999 | R |
| 302 | Aspartyl-tRNA Syntetase | DARS | 6.1.1.12 | | Translation | trna[c] + asp[c] + atp <==> trna-asp[c] + amp + 2 p | -999999 | 999999 | R |
| 303 | Glycyl-tRNA Syntetase | GARS | 6.1.1.14 | | Translation | trna[c] + gly[c] + atp <==> trna-gly[c] + amp + 2 p | -999999 | 999999 | R |
| 304 | Prolyl-tRNA Syntetase | PARS | 6.1.1.15 | | Translation | trna[c] + pro[c] + atp <==> trna-pro[c] + amp + 2 p | -999999 | 999999 | R |
| 305 | Cysteinyl-tRNA Syntetase | CARS | 6.1.1.16 | | Translation | trna[c] + cys[c] + atp <==> trna-cys[c] + amp + 2 p | -999999 | 999999 | R |
| 306 | Glutamyl-tRNA Syntetase | EPRS | 6.1.1.17 | | Translation | trna[c] + glu[c] + atp <==> trna-glu[c] + amp + 2 p | -999999 | 999999 | R |
| 307 | Glutaminyl-tRNA Syntetase | QARS | 6.1.1.18 | | Translation | trna[c] + gln[c] + atp <==> trna-gln[c] + amp + 2 p | -999999 | 999999 | R |
| 308 | Arginyl-tRNA Syntetase | RARS | 6.1.1.19 | | Translation | trna[c] + arg[c] + atp <==> trna-arg[c] + amp + 2 p | -999999 | 999999 | R |
| 309 | Phenoalanyl-tRNA Syntetase | FARS | 6.1.1.20 | | Translation | trna[c] + phe[c] + atp <==> trna-phe[c] + amp + 2 p | -999999 | 999999 | R |
| 310 | HIstidyl-tRNA Syntetase | HARS | 6.1.1.21 | | Translation | trna[c] + his[c] + atp <==> trna-his[c] + amp + 2 p | -999999 | 999999 | R |
| 311 | Asparaginyl-tRNA Syntetase | NARS | 6.1.1.22 | | Translation | trna[c] + asn[c] + atp <==> trna-asn[c] + amp + 2 p | -999999 | 999999 | R |
| 312 | Actin Synthesis | ACT | 4501881 | | Protein Synthesis | 29 trna-ala[c] + 6 trna-cys[c] + 22 trna-asp[c] + 28 trna-glu[c] + 12 trna-phe[c | 0 | 999999 | IR |
| 313 | Myosin Heavy Chain type 1 and 2x muscle fiber | MYOHC1-2x | 115527082 | | Protein Synthesis | 171 trna-ala[c] + 17 trna-cys[c] + 98 trna-asp[c] + 261 trna-glu[c] + 56 trna-ph | 0 | 999999 | IR |
| 314 | Myosin Heavy Chain type 2a muscle fiber | MYOHC2a | 153792663 | | Protein Synthesis | 172 trna-ala[c] + 18 trna-cys[c] + 93 trna-asp[c] + 265 trna-glu[c] + 58 trna-ph | 0 | 999999 | IR |
| 315 | Myosin Heavy Chain type 2b muscle fiber | MYOHC2b | 110611903 | | Protein Synthesis | 168 trna-ala[c] + 17 trna-cys[c] + 94 trna-asp[c] + 263 trna-glu[c] + 61 trna-ph | 0 | 999999 | IR |
| 316 | Myosin Light Chain Kinase | MYOLCk | 14993776 | | Protein Synthesis | 56 trna-ala[c] + 10 trna-cys[c] + 30 trna-asp[c] + 48 trna-glu[c] + 20 trna-phe[ | 0 | 999999 | IR |
| 317 | Myosin Light Chain Phosphorylated | MYOLCp | 28372499 | | Protein Synthesis | 12 trna-ala[c] + 2 trna-cys[c] + 15 trna-asp[c] + 15 trna-glu[c] + 12 trna-phe[c | 0 | 999999 | IR |
| 318 | Myosin type 1 muscle fiber | MYO1-2x | | | Polymerizatin | 2 myoHC1-2x[c] + 2 myoLCk[c] + 2 myoLCp[c] --> myo1[c] | 0 | 999999 | IR |
| 319 | Myosin type 2a muscle fiber | MYO2a | | | Polymerizatin | 2 myoHC2a[c] + 2 myoLCk[c] + 2 myoLCp[c] --> myo2a[c] | 0 | 999999 | IR |
| 320 | Myosin type 2x muscle fiber | MYO2x | | | Polymerizatin | 2 myoHC1-2x[c] + 2 myoLCk[c] + 2 myoLCp[c] --> myo2x[c] | 0 | 999999 | IR |
| 321 | Myosin type 2b muslce fiber | MYO2b | | | Polymerizatin | 2 myoHC2b[c] + 2 myoLCk[c] + 2 myoLCp[c] --> myo2b[c] | 0 | 999999 | IR |
| 322 | Tropomyosin type 1 muscle fiber | TMN1 | 114155140 | | Protein Synthesis | 38 trna-ala[c] + 1 trna-cys[c] + 19 trna-asp[c] + 61 trna-glu[c] + 1 trna-phe[c] | 0 | 999999 | IR |
| 323 | Tropomyosin type 2 muscle fiber | TMN2 | 114155144 | | Protein Synthesis | 28 trna-ala[c] + 3 trna-cys[c] + 14 trna-asp[c] + 54 trna-glu[c] + 1 trna-phe[c] | 0 | 999999 | IR |
| 324 | Tropomyosin complex for type 1 muscle fibers | DIM-TMN1 | | | Polymerizatin | 2 tmn1[c] --> dim-tmn1[c] | 0 | 999999 | IR |
| 325 | Tropomyosin complex for type 2 muscle fibrers | DIM-TMN2 | | | Polymerizatin | 2 tmn2[c] --> dim-tmn2[c] | 0 | 999999 | IR |
| 326 | Troponin c for type 1 muscle fibers | TPNC1 | 4507615 | | Protein Synthesis | 7 trna-ala[c] + 2 trna-cys[c] + 23 trna-asp[c] + 23 trna-glu[c] + 9 trna-phe[c] + | 0 | 999999 | IR |
| 327 | Troponin c for type 2 muscle fibers | TPNC2 | 4507617 | | Protein Synthesis | 12 trna-ala[c] + 1 trna-cys[c] + 19 trna-asp[c] + 26 trna-glu[c] + 10 trna-phe[c | 0 | 999999 | IR |
| 328 | Troponin I for type 1 muscle fibers | TPNI1 | 56682969 | | Protein Synthesis | 15 trna-ala[c] + 3 trna-cys[c] + 10 trna-asp[c] + 22 trna-glu[c] + 2 trna-phe[c] | 0 | 999999 | IR |
| 329 | Troponin I for type 2 muscle fibers | TPNI2 | 4507621 | | Protein Synthesis | 13 trna-ala[c] + 3 trna-cys[c] + 11 trna-asp[c] + 25 trna-glu[c] + 3 trna-phe[c] | 0 | 999999 | IR |
| 330 | Troponin T for type 1 muscle fibers | TPNT1 | 187173288 | | Protein Synthesis | 20 trna-ala[c] + 1 trna-cys[c] + 12 trna-asp[c] + 54 trna-glu[c] + 6 trna-phe[c] | 0 | 999999 | IR |
| 331 | Troponin T for type 2 muslce fibers | TPNT2 | 5803203 | | Protein Synthesis | 24 trna-ala[c] + 18 trna-asp[c] + 49 trna-glu[c] + 4 trna-phe[c] + 9 trna-gly[c] + | 0 | 999999 | IR |
| 332 | Troponin for type 1 muscle fibers | TPN1 | | | Polymerizatin | tpnc1[c] + tpni1[c] + tpnt1[c] --> tpn1[c] | 0 | 999999 | IR |
| 333 | Troponin for type 2 muslce fibers | TPN2 | | | Polymerizatin | tpnc2[c] + tpni2[c] + tpnt2[c] --> tpn2[c] | 0 | 999999 | IR |
| 334 | Contractile complex for type 1 muscle fibers | CONTR-COMP1 | | | Polymerizatin | 7 act[c] + 7 myo1[c] + dim-tmn1[c] + tpn1[c] --> contr-comp1[c] | 0 | 999999 | IR |
| 335 | Contractile complex for type 2a muscle fibers | CONTR-COMP2a | | | Polymerizatin | 7 act[c] + 7 myo2a[c] + dim-tmn2[c] + tpn2[c] --> contr-comp2a[c] | 0 | 999999 | IR |

| 0 | Enzyme | Abrev | EC Number | Location | Pathway | Reaction | Lower Bound | Upper Bound | Thermodynamics |
|---|---|---|---|---|---|---|---|---|---|
| 336 | Contractile complex for type 2x muscle fibers | CONTR-COMP2x | | | Polymerizatin | 7 act[c] + 7 myo2x[c] + dim-tmn2[c] + tpn2[c] --> contr-comp2x[c] | 0 | 999999 | IR |
| 337 | Contractile complex for type 2b muscle fibers | CONTR-COMP2b | | | Polymerizatin | 7 act[c] + 7 myo2b[c] + dim-tmn2[c] + tpn2[c] --> contr-comp2b[c] | 0 | 999999 | IR |
| 338 | Storage Contractile complex type 1 muscle fibers | Str_CONTR-COMP1 | | | Muscle | contr-comp1[c] --> | 0 | 999999 | IR |
| 339 | Storage Contractile complex type 2a muscle fibers | Str_CONTR-COMP2a | | | Muscle | contr-comp2a[c] --> | 0 | 999999 | IR |
| 340 | Storage Contractile complex type 2x muscle fibers | Str_CONT-COMP2x | | | Muscle | contr-comp2x[c] --> | 0 | 999999 | IR |
| 341 | Storage Contractile complex type 2b muscle fibers | Str_CONTR-COMP2b | | | Muscle | contr-comp2b[c] --> | 0 | 999999 | IR |
| 342 | Creatine Kinase, M Chain | CKM | 2.7.3.2 | | Creatine | cr-p + adp <==> cr + atp | -999999 | 999999 | R |
| 343 | Creatine Supply | Spont_CrP | | | Creatine | cr-p <==> cr + p | -999999 | 999999 | R |
| 344 | | Nut_cr | | | Creatine | cr <==> | 0 | 999999 | IR |
| 345 | Fake Transfer | ATP2UTP | | | Glycogen Synthesis 2 | atp --> utp | 0 | 999999 | |
| 346 | Fake Transfer | UDP2ADP | | | Glycogen Synthesis | udp --> adp | 0 | 999999 | |
| 347 | Phosphoglucomutase | PGM1 | 5.4.2.2 | | Glycogen Synthesis | g6p[c] <==> g1p[c] | -999999 | 999999 | IR |
| 348 | UTP-glucose--1-phosphate uridylyltransferase | UGP2 | 2.7.7.9 | | Glycogen Synthesis 2 | g1p[c] + utp --> udp-g1p[c] + (2) p | -999999 | 999999 | |
| 349 | Modified Glycogenin-1 and 2 for model | mGYG | 2.4.1.186/2.4.1.11 | | Glycogen Synthesis | udp-g1p[c] <==> glycogen[c] + udp | -999999 | 999999 | |
| 350 | Modified Glycogen Synthase - muscle for the model | mGYS1 | | | Glycogen Synthesis | glycogen[c] --> | 0 | 999999 | |
| 351 | glucose-6-phosphate 1-dehydrogenase | G6PD | 1.1.1.49 | cytosol | Pentose Phosphate Pathway | g6p[c] + nadp[c] <==> gluc-lac-6p[c] + nadph[c] | -999999 | 999999 | |
| 352 | 6-phosphogluconalactonase | PGLS | 3.1.1.31 | cytosol | Pentose Phosphate Pathway | gluc-lac-6p[c] + h2o <==> 6pgluconate[c] | -999999 | 999999 | |
| 353 | 6-phosphogluconate dehydrogenase | PGD | 1.1.1.44 | cytosol | Pentose Phosphate Pathway | 6pgluconate[c] + nadp[c] <==> ru5p[c] + co2 + nadph[c] | -999999 | 0 | |
| 354 | 6-phosphogluconate dehydrogenase | PGD | 1.1.1.44 | cytosol | Pentose Phosphate Pathway | 6pgluconate[c] + nad[c] <==> ru5p[c] + co2 + nadh[c] | 0 | 999999 | |
| 355 | ribulose-5-phosphate-3-epimerase | RPE | 5.1.3.1 | cytosol | Pentose Phosphate Pathway | ru5p[c] <==> x5p[c] | -999999 | 999999 | |
| 356 | ribose-5-phosphate isomerase | RPIA | 5.3.1.6 | cytosol | Pentose Phosphate Pathway | ru5p[c] <==> r5p[c] | -999999 | 999999 | |
| 357 | transketolase | TKT1 | 2.2.1.1 | cytosol | Pentose Phosphate Pathway | e4p[c] + x5p[c] <==> f6p[c] + g3p[c] | -999999 | 999999 | |
| 358 | transketolase | TKT2 | 2.2.1.1 | cytosol | Pentose Phosphate Pathway | s7p[c] + g3p[c] <==> r5p[c] + x5p[c] | -999999 | 999999 | |
| 359 | transaldolase | TALDO | 2.2.1.2 | cytosol | Pentose Phosphate Pathway | s7p[c] + g3p[c] <==> e4p[c] + f6p[c] | -999999 | 999999 | |
| 360 | Ribokinase | RBSK | 2.7.1.15 | cytosol | Pentose Phosphate Pathway | rib[c] + atp <==> r5p[c] + adp + p | -999999 | 999999 | |
| 361 | Glutamate-cystein Ligase | GCL | 6.3.2.2 | Cytosol | Glutathione biosysnthesis | cys[c] + glu[c] + atp --> glu-cys[c] + p + adp | 0 | 999999 | |
| 362 | Glutathione synthetase | GSS | 6.3.2.3 | Cytosol | Glutathione biosysnthesis | glu-cys[c] + gly[c] + atp --> glutath[c] + adp + p | 0 | 999999 | |
| 363 | Glutahione transport | glutathxport | | | Mitchondrial Transport | glutath[c] <==> glutath[m] | -999999 | 999999 | |
| 364 | Glutahione reductase, cellular | GSR | 1.8.1.7 | Cytosol | Glutathione Redox | 2 glutath[c] + nadp[c] --> gssg[c] + nadph[c] | 0 | 999999 | |
| 365 | Glutahione reductase | GSR | 1.8.1.7 | Mitochondria | Glutathione Redox | 2 glutath[m] + nadp[m] --> gssg[m] + nadph[m] | 0 | 999999 | |
| 366 | Gluathione leak | GlutathLeak | | | Cellular Export | gssg[c] --> | 0 | 999999 | |
| 367 | Gluathione leak | GlutathLeak | | | Cellular Export | gssg[m] --> | 0 | 999999 | |
| 368 | | Acetyl-coaLeak | | | Cellular Export | acetyl-coa[m] --> | 0 | 999999 | |
