# Supplementary material for: To Supplement or Not to Supplement: A Metabolic Network Framework for Human Nutritional Supplements
Source: PLoS One. 2013 Aug 5;8(8):e68751. doi: 10.1371/journal.pone.0068751 (PMC3740736; doi:10.1371/journal.pone.0068751)
Supplement: File S4 — Results: fasting. (PDF) [file pone.0068751.s005.pdf]

Fasting		Type 2a	Single		Double
	Available	Control	Methionin	Difference	ile-met
Prot Synth	n/a	2.18E-05	2.83E-05	6.52E-06	3.15E-05
ARG	0.08	-0.047	-0.0604	-0.0139	-0.06712
HIS	0.082	-0.016	-0.0208	-0.0048	-0.02313
ILE	0.062	-0.062	-0.062	0.0000	-0.06894
LEU	0.123	-0.123	-0.1106	0.0124	-0.123
LYS	0.188	-0.179	-0.1169	0.0620	-0.13002
MET	0.025	-0.025	-0.0325	-0.0075	-0.03612
PHE	0.057	-0.047	-0.057	-0.0099	-0.057
THR	0.14	-0.047	-0.0615	-0.0142	-0.06841
TRP	0.044	-0.005	-0.0064	-0.0015	-0.00714
VAL	0.233	-0.233	-0.1466	0.0864	-0.17861
ALA	0.333	-0.333	-0.333	0.0000	-0.333
ASN	0.041	0.000	-0.041	-0.0410	-0.041
ASP	0.003	-0.003	0	0.0030	0
CYS	0.052	-0.010	-0.0134	-0.0031	-0.01485
GLU	0.024	-0.024	-0.024	0.0000	-0.024
GLN	0.586	0.000	-0.0717	-0.0717	-0.0843
GLY	0.23	-0.047	-0.0613	-0.0142	-0.06812
PRO	0.168	-0.168	-0.168	0.0000	-0.168
SER	0.114	-0.046	-0.0157	0.0304	-0.02341
TYR	0.059	0.000	-0.0042	-0.0042	-0.011

	Available					
	0	1	2 HR	3	4	0-2 hr diff
ARG	0.061	0.098	0.120	0.106	0.092	0.059
HIS	0.092	0.105	0.155	0.105	0.106	0.063
ILE	0.067	0.105	0.144	0.155	0.137	0.077
LEU	0.14	0.205	0.271	0.282	0.247	0.131
LYS	0.189	0.277	0.328	0.281	0.259	0.139
MET						
PHE	0.049	0.062	0.068	0.069	0.06	0.019
THR	0.149	0.185	0.214	0.212	0.19	0.065
TRP						
VAL	0.238	0.3	0.359	0.395	0.366	0.121
ALA	0.271	0.34	0.345	0.324	0.289	0.074
ASN						
ASP	0.115	0.114	0.114	0.116	0.112	-0.001
CYS						
GLU	0.145	0.148	0.152	0.148	0.149	0.007
GLN	0.658	0.662	0.676	0.653	0.66	0.018
GLY	0.352	0.387	0.377	0.367	0.345	0.025
PRO	0.179	0.223	0.231	0.227	0.211	0.052
SER	0.118	0.209	0.217	0.197	0.183	0.099
TYR	0.063	0.077	0.092	0.093	0.082	0.029

							0.061	
	**Basal**						**Postabsorptive**	
	**Cynober**	**Aoki**	**Pozefsky**	**Tessari**	**AVG**	**STD**		**Aoki**
**ARG**	0.08	0.061	0.075	0.073	**0.072**	0.008		0.120
**HIS**	0.082	0.092	0.076	0.06	**0.078**	0.013		0.155
**ILE**	0.062	0.067	0.056	0.053	**0.060**	0.006		0.144
**LEU**	0.123	0.14	0.12		**0.128**	0.011		0.271
**LYS**	0.188	0.189	0.172	0.144	**0.173**	0.021		0.328
**MET**	0.025		0.021	0.014	**0.020**	0.006		
**PHE**	0.057	0.049	0.049		**0.052**	0.005		0.068
**THR**	0.14	0.149	0.135	0.107	**0.133**	0.018		0.214
**TRP**	0.044		0.038		**0.041**	0.004		
**VAL**	0.233	0.238	0.238	0.211	**0.230**	0.013		0.359
**ALA**	0.333	0.271	0.231	0.24	**0.269**	0.046		0.345
**ASN**	0.041			0.025	**0.033**	0.011		
**ASP**	0.003	0.115		0.016	**0.045**	0.061		0.114
**CYS**	0.052		0.086		**0.069**	0.024		
**GLU**	0.024	0.145		0.152	**0.107**	0.072		0.152
**GLN**	0.586	0.658		0.366	**0.537**	0.152		0.676
**GLY**	0.23	0.352	0.179	0.191	**0.238**	0.079		0.377
**PRO**	0.168	0.179	0.178	0.215	**0.185**	0.021		0.231
**SER**	0.114	0.118	0.107	0.109	**0.112**	0.005		0.217
**TYR**	0.059	0.063	0.054	0.047	**0.056**	0.007		0.092

	Triple		Quadruple	
**Difference**	**Ile-Leu-Met**	**Difference**	**Arg-Ile-Leu-Met**	**Difference**
9.69E-06	3.75E-05	1.57E-05	4.18E-05	2.00E-05
-0.0206	-0.080	-0.0335	-0.089	-0.0427
-0.0071	-0.028	-0.0116	-0.031	-0.0147
-0.0069	-0.108	-0.0459	-0.138	-0.0757
0.0000	-0.147	-0.0236	-0.163	-0.0405
0.0489	-0.155	0.0239	-0.173	0.0061
-0.0111	-0.043	-0.0181	-0.048	-0.0230
-0.0099	-0.057	-0.0099	-0.057	-0.0099
-0.0211	-0.082	-0.0342	-0.091	-0.0436
-0.0022	-0.009	-0.0036	-0.009	-0.0046
0.0544	-0.206	0.0272	-0.223	0.0104
0.0000	-0.333	0.0000	-0.333	0.0000
-0.0410	-0.041	-0.0410	-0.041	-0.0410
0.0030	0.000	0.0030	0.000	0.0030
-0.0046	-0.018	-0.0074	-0.020	-0.0094
0.0000	-0.024	0.0000	-0.024	0.0000
-0.0843	-0.108	-0.1083	-0.126	-0.1255
-0.0210	-0.081	-0.0341	-0.091	-0.0434
0.0000	-0.168	0.0000	-0.168	0.0000
0.0227	-0.046	-0.0001	-0.063	-0.0169
-0.0110	-0.024	-0.0241	-0.033	-0.0334

	Mixed Meal (Tessari et al)	
Cynober - Aoki	Basal	Meal
0.019	0.073	0.11
-0.01	0.06	0.081
-0.005	0.053	0.112
-0.017		
-0.001	0.144	0.26
	0.014	0.03
0.008		
-0.009	0.107	0.149
-0.005	0.211	0.339
0.062	0.24	0.272
	0.025	0.045
-0.112	0.016	0.02
-0.121	0.152	0.152
-0.072	0.366	0.405
-0.122	0.191	0.185
-0.011	0.215	0.367
-0.004	0.109	0.133
-0.004	0.047	0.091

-0.025

(post insulin)

**Posefsky**	**Tessari**	**AVG**	**STD**	**Diff b/n AVG**	**%Dif**
	0.11	**0.115**	0.007	0.043	59%
	0.081	**0.118**	0.052	0.041	52%
	0.112	**0.128**	0.023	0.069	115%
		**0.271**		0.143	112%
	0.26	**0.294**	0.048	0.121	70%
	0.03	**0.030**		0.010	50%
		**0.068**		0.016	32%
	0.149	**0.182**	0.046	0.049	37%
		**0.044**			
	0.339	**0.349**	0.014	0.119	52%
	0.272	**0.309**	0.052	0.040	15%
	0.045	**0.045**		0.012	36%
	0.02	**0.067**	0.066	0.022	50%
0.093		**0.093**			
	0.152	**0.152**	0.000	0.045	42%
	0.405	**0.541**	0.192	0.004	1%
	0.185	**0.281**	0.136	0.043	18%
	0.367	**0.299**	0.096	0.114	62%
	0.133	**0.175**	0.059	0.063	56%
	0.091	**0.092**	0.001	0.036	64%

Quintiple			Hextuple			Heptuple
Arg-Ile-Leu-Met-Phe	Difference		Arg-Ile-Leu-Lys-Met-Phe	Difference		Arg-Ile-Leu
4.55E-05	2.37E-05		6.44E-05	4.26E-05		0.000106
-0.097	-0.0505		-0.137	-0.0909		-0.2266
-0.033	-0.0174		-0.047	-0.0313		-0.07808
-0.238	-0.1763		-0.383	-0.3211		-1.07772
-0.178	-0.0549		-0.252	-0.1287		-0.41527
-0.188	-0.0091		-0.266	-0.0872		-0.43897
-0.052	-0.0272		-0.074	-0.0489		-0.12196
-0.062	-0.0149		-0.088	-0.0407		-0.22958
-0.099	-0.0516		-0.140	-0.0927		-0.23096
-0.010	-0.0054		-0.015	-0.0097		-0.02412
-0.140	0.0932		-0.233	0.0000		-0.233
-0.333	0.0000		-0.333	0.0000		-0.333
-0.041	-0.0410		-0.041	-0.0410		-0.041
-0.003	0.0000		-0.003	0.0000		-0.003
-0.021	-0.0112		-0.030	-0.0201		-0.05014
-0.024	0.0000		-0.024	0.0000		-0.024
-0.140	-0.1402		-0.215	-0.2154		-0.38203
-0.099	-0.0514		-0.139	-0.0923		-0.23
-0.168	0.0000		-0.168	0.0000		-0.168
-0.055	-0.0086		-0.095	-0.0486		-0.114
-0.036	-0.0363		-0.051	-0.0514		0

avg from Cynober

Difference
8.45E-05
-0.1801
-0.0621
-1.0157
-0.2923
-0.2601
-0.0970
-0.1825
-0.1837
-0.0192
0.0000
0.0000
-0.0410
0.0000
-0.0398
0.0000
-0.3820
-0.1829
0.0000
-0.0679
0.0000

		1	2	3
Flux	Control	Met	**ile-met**	**Ile-Leu-Met**
Obj_atp	0	0	0	0
Ex_ins	0	0	-0.02019	-0.03017
Ex_gluc	0	0	-0.02019	-0.03017
Ex_lac	0	0	0	0.00001
Ex_pyr	0	0	0	0
Ex_pal	0	0	-0.125	-0.125
Ex_prop	0	0	0	0
Ex_but	0	0	0	0
Ex_5c	0	0	0	0
Ex_6c	0	0	0	0
Ex_8c	0	0	0	0
Ex_9c	0	0	0	0
Ex_10c	0	0	0	0
Ex_12c	0	0	0	0
Ex_14c	0	0	0	-0.23
Ex_18c	0	0	0	0
Ex_glyc	0	0	0	0
Ex_tag	0	0	0	0
Ex_dag	0	0	0	0
Ex_mag	0	0	0	0
Ex_akg	0	0	0	0
Ex_oaa	0	0	0	0
Ex_rib	0	0	0	0
Nut_o2	-0.3368	-0.44567	-0.50314	-8.64264
Nut_co2	0.130971	0.15363	0.278516	6.075709
Nut_h2o	2.114062	2.77549	3.327205	-999940
Nut_p	0	0	0.020193	0.090499
Nut_hco3	0	0	-0.07139	-0.12421
Nut_coa	0	0	0	-0.23
Nut_carn	0	0	0	0
Nut_dhf	0	0	0	0
LPL	0	0	0	0
DAGL	0	0	0	0
12DAG	0	0	0	0
MAGL	0	0	0	0
GLUT4	0	0	0.020193	0.030166
MCT4	0	0	0	-0.00001
MCT_pyr	0	0	0	0
FAT/CD36	0	0	0.125	0.125
FATprop	0	0	0	0
FATbut	0	0	0	0
FAT5c	0	0	0	0
FAT6c	0	0	0	0
FAT8c	0	0	0	0
FAT9c	0	0	0	0

FAT10c	0	0	0	0
FAT12c	0	0	0	0
FAT14c	0	0	0	0.23
FAT18c	0	0	0	0
Alc_glyc	0	0	0	0
COA	0	0	0	0.23
SLC22A5/O	0	0	0	0
Xport_rib	0	0	0	0
Dr_akg	0	0	0	0
Dr_oaa	0	0	0	0
Hex	0	0	0.020193	0.030166
PGI	0	0	0.020183	0.030166
PFK	0	0	0.02019	0.030166
FBP	0	0	0	0
Ald	0	0	0.02019	0.030166
TPI	0	0	0.020193	-0.03017
D3PDeh	0	0	0	0.060333
PGK	0	0	0	0.060333
PGM	-0.04194	-0.0442	-0.04038	0.060333
Eno	-0.04194	-0.0442	-0.04038	0.060333
PyrK	0	0	-0.04038	999997.3
PEPSynth	0	0	0	999997.3
PEPCarbK	0	0	0	0
PEPCarbl	0.041944	0.0442	0	0
LacDeh	0	0	0	-0.00001
MalDehC(n	0.024566	0.02577	0.00001	0.00001
ASTC	0.066511	0.06997	0.00001	0.00001
G3PDehC	0	0	0.040383	0
Mal-AKGEX	0.024566	0.02577	0.00001	0.00001
Glu-AspEx	0.113593	0.1352	0.074133	0.137807
MCT	0	0	0	0.101062
ASTM	0.113593	0.1352	0.074133	0.137807
G3PDehM	0	0	0	0
PyrSynth/P	0	0	0	0.101062
CitSynth	0	0	0.102191	2.901717
Acon	0	0	0.102191	2.901717
IsoDeh	0	0	0.102191	0
IsoDehnad	0	0	0	2.901717
AKGDeh	0.054718	0.017662	0.104924	2.755376
SunCoaSyn	0.089026	0.10943	0.176314	3.039125
SucDeh(I)	0.089026	0.10943	0.176314	3.039514
Fum	0.089026	0.10943	0.176314	3.039514
MalDehM	0.113593	0.1352	0.176324	3.039524
NADHDeh(	0.410752	0.559151	0.676163	8.709936
ETFUO	0.262851	0.332184	0.330108	8.575344
UCytC(III)	0.673602	0.891335	1.006272	17.28528
CytCO(IV)	0.336801	0.445668	0.503136	8.64264

ATPSynth(\	1.757956	2.341822	2.688707	43.2805
AMP_ADPs	0.906337	1.181767	1.443671	999999
PalTK	0	0	0.125	0.125
CPT1B/CHK	0	0	0.00385	0.125
SLC25A20/	0	0	0.00385	0.125
CPT2	0	0	0.00385	0.125
CRAT(mit)	0	0	0	0
CRAT Trans	0	0	0	0
CRAT(cyt)	0	0	0	0
Acetyl-coaL	0	0	0	0
AcylCoADe	0	0	0.00385	0.125
EnoCoADel	0	0	0.00385	0.125
3HA-CoADe	0	0	0.00385	0.125
AcylCoAAT	0	0	0.00385	0.125
AcylCoADe	0	0	0	0
EnoCoADel	0	0	0	0
3HA-CoADe	0	0	0	0
AcylCoAAT	0	0	0	0
AcylCoADe	0	0	0.00385	0.355
EnoCoADel	0	0	0.00385	0.355
3HA-CoADe	0	0	0.00385	0.355
AcylCoAAT	0	0	0.00385	0.355
AcylCoADe	0	0	0.00385	0.355
EnoCoADel	0	0	0.00385	0.355
3HA-CoADe	0	0	0.00385	0.355
AcylCoAAT	0	0	0.00385	0.355
AcylCoADe	0	0	0.00385	0.355
EnoCoADel	0	0	0.00385	0.355
3HA-CoADe	0	0	0.00385	0.355
AcylCoAAT	0	0	0.00385	0.355
AcylCoADe	0	0	0.00385	0.355
EnoCoADel	0	0	0.00385	0.355
3HA-CoADe	0	0	0.00385	0.355
AcylCoAAT	0	0	0.00385	0.355
AcylCoADe	0	0	0.00385	0.355
EnoCoADel	0	0	0.00385	0.355
3HA-CoADe	0	0	0.00385	0.355
AcylCoAAT	0	0	0.00385	0.355
ButyrlCoAD	0	0	0.00385	0.355
EnoCoADel	0	0	0.00385	0.388026
3HA-CoADe	0	0	0.00385	0.388026
AcylCoAAT	0	0	0.00385	0.388415
GK	0	0	0	0
GPAT4	0	0	0.040383	0
ABHD5	0	0	0.040383	0
DAGZ/DAG	0	0	0.040383	0
DGAT1	0	0	0.040383	0

St_TAG	0	0	0.040383	0
ACAS2	0	0	0	0
ACSM-But	0	0	0	0
ASCM-5c	0	0	0	0
ASCM-6c	0	0	0	0
ACSM-8c	0	0	0	0
ACSM-9c	0	0	0	0
ACSM-10c	0	0	0	0
ACSM-12c	0	0	0	0
ACSM-14c	0	0	0	0.23
ACSM-18c	0	0	0	0
CPT1B/CHK	0	0	0	0
CPT1B/CHK	0	0	0	0
CPT1B/CHK	0	0	0	0
CPT1B/CHK	0	0	0	0
CPT1B/CHK	0	0	0	0
CPT1B/CHK	0	0	0	0
CPT1B/CHK	0	0	0	0
CPT1B/CHK	0	0	0	0
CPT1B/CHK	0	0	0	0.23
CPT1B/CHK	0	0	0	0
SLC25A20/	0	0	0	0
SLC25A20/	0	0	0	0
SLC25A20/	0	0	0	0
SLC25A20/	0	0	0	0
SLC25A20/	0	0	0	0
SLC25A20/	0	0	0	0
SLC25A20/	0	0	0	0
SLC25A20/	0	0	0	0
SLC25A20/	0	0	0	0.23
SLC25A20/	0	0	0	0
CPT2	0	0	0	0
CPT2	0	0	0	0
CPT2	0	0	0	0
CPT2	0	0	0	0
CPT2	0	0	0	0
CPT2	0	0	0	0
CPT2	0	0	0	0
CPT2	0	0	0	0
CPT2	0	0	0	0.23
CPT2	0	0	0	0
AcylCoADe	0	0	0	0
EnoCoADel	0	0	0	0
3HA-CoADe	0	0	0	0
AcylCoAAT	0	0	0	0
AcylCoADe	0	0	0	0
EnoCoADel	0	0	0	0

3HA-CoADe	0	0	0	0
AcylCoAAT-	0	0	0	0
AcylCoADe	0	0	0	0
EnoCoADel	0	0	0	0
3HA-CoADe	0	0	0	0
AcylCoAAT-	0	0	0	0
AKR1A1	0	0	0	0
ALDH3A2	0	0	0	0
GLYCTK	0	0	0	0
EAA_arg	-0.04645	-0.06036	-0.06712	-0.08
EAA_his	-0.01601	-0.0208	-0.02313	-0.082
EAA_ile	-0.04771	-0.062	-0.14033	-0.206
EAA_leu	-0.08513	-0.11062	-0.123	-0.147
EAA_lys	-0.08998	-0.11693	-0.13002	-0.188
EAA_met	-0.025	-0.03249	-0.03612	-0.04306
EAA_phe	-0.04706	-0.057	-0.04289	-0.057
EAA_thr	-0.04734	-0.06152	-0.06841	-0.08154
EAA_trp	-0.00494	-0.00642	-0.00714	-0.00851
EAA_val	-0.07651	-0.14661	-0.06098	-0.233
NEAA_ala	-0.333	-0.333	-0.333	-0.333
NEAA_asn	-0.03467	-0.041	-0.041	0
NEAA_asp	0	0	-0.003	-0.003
NEAA_cys	-0.01028	-0.01336	-0.01485	-0.0177
NEAA_glu	-0.024	-0.024	-0.024	-0.024
NEAA_gln	-0.05205	-0.07168	-0.0843	0
NEAA_gly	-0.04715	-0.06126	-0.06812	-0.13563
NEAA_pro	-0.168	-0.168	-0.168	-0.168
NEAA_ser	-0.00418	-0.01573	-0.02626	-0.06574
NEAA_tyr	0	-0.00415	-0.02511	-0.02405
DLDH	0.034309	0.091768	0.07139	0.284528
MT_val	0.034309	0.091768	0	0.160314
BCAT2-val	0.034309	0.091768	0	0.160314
BCKDHB-va	0.034309	0.091768	0	0.160314
BCKADE2-v	0.034309	0.091768	0	0.160314
ACADSB	0.034309	0.091768	0	0.160314
ECHS1	0.034309	0.091768	0	0.160314
HIBCH	0.034309	0.091768	0	0.160314
HIBADH	0.034309	0.091768	0	0.160314
ALDH6A1	0.034309	0.091768	0	0.160314
PCCB	0.034309	0.091768	0.07139	0.284138
MCEE	0.034309	0.091768	0.07139	0.284138
MUT	0.034309	0.091768	0.07139	0.284138
MT_ile	0	0	0.07139	0.123825
BCAT-ile	0	0	0.07139	0.123825
BCKDHB-ile	0	0	0.07139	0.123825
BCKADE2-il	0	0	0.07139	0.123825
ECHS1	0	0	0.07139	0.123825

HSD17B10	0	0	0.07139	0.123825
ACAT1	0	0	0.07139	0.123825
MT_leu	0	0	0	0.00039
BCAT2-leu	0	0	0	0.00039
BCKDHB-le	0	0	0	0.00039
BCKADE2-l	0	0	0	0.00039
IVD	0	0	0	0.00039
MCC1,MCC	0	0	0	0.00039
MGCA	0	0	0	0.00039
HMGCL	0	0	0	0.00039
OXCT	0	0	0	0.00039
HMGCS2	0	0	0	0
GluDeh	0	0	0	0.066441
GlnSynth	0	0	0	0.216047
GLN_Elim	0	0	0	0.066699
ALT	0	0	-0.04038	0
ALA_Elim	0.253122	0.229204	0.1772	0.195429
SDS	0	0	0	0.040739
CBS	0	0	0	0
CTH	0	0	0	0
ILVBL	0	0	0	0
2a2hButLe	0	0	0	0
MT_lys	0	0	0	0.033026
AASS	0	0	0	0.033026
AASS	0	0	0	0.033026
aAASADeh	0	0	0	0.033026
KATII	0	0	0	0.033026
lysdeg	0	0	0	0.033026
GCDH	0	0	0	0.033026
MT_pro	0.139516	0.130987	0.126843	0.118942
PRODH	0.139516	0.130987	0.126843	3.087101
ALDH4a1	0.139516	0.130987	0.126843	0.118942
GATM	0	0	0	0
Dr_orn	0	0	0	0
GAMT	0	0	0	0
Dr_cr	0	0	0	0
AdoHcyase	0	0	0	0
MTR	0	0	0	0
AdoMet	0	0	0	0
SHMT	0	0	0	-0.05443
MTHFR	0	0	0	0
DHFR	0	0	0	0
HAL	0	0	0	0.054433
FLJ31300	0	0	0	0.054433
MGC35366	0	0	0	0.054433
FTCD1	0	0	0	0.054433
FTCD2	0	0	0	0.054433

FT	0	0	0	0.054433
ASNase	0	0	0	0
ASNSynth	0	0.00405	0.009094	0.059709
PHGDH	0.041944	0.0442	0.040383	0
PSA	0.041944	0.0442	0.040383	0
PSPH	0.041944	0.0442	0.040383	0
P5CS	0	0	0	0
PYCS	0	0	0	0
Spont	0	0	0	0
PYRCR1	0	0	0	2.968159
PAG	0.017378	0.01843	0	0.00588
PCBD	0.017378	0.01843	0	0.00588
QDPR	0.017378	0.01843	0	0.00588
MT_glu	0.173824	0.222754	0.198233	0.403081
tRNA	0	0	0	0
YARS	0.017378	0.022581	0.02511	0.02993
WARS	0.004943	0.006424	0.007143	0.008514
TARS	0.047343	0.061519	0.068407	0.081538
LARS	0.085126	0.110615	0.123	0.14661
IARS	0.047713	0.062	0.068942	0.082175
KARS	0.089983	0.116926	0.130017	0.154974
AARS	0.079878	0.103796	0.115417	0.137571
VARS	0.042204	0.054841	0.060981	0.072686
MARS	0.025	0.032486	0.036123	0.043057
SARS	0.046124	0.059934	0.066645	0.079437
DARS	0.047082	0.061179	0.068029	0.081088
GARS	0.047147	0.061264	0.068124	0.0812
PARS	0.028484	0.037013	0.041157	0.049058
CARS	0.010279	0.013356	0.014852	0.017703
EPRS	0.108798	0.141375	0.157203	0.187379
QARS	0.052047	0.067631	0.075203	0.089639
RARS	0.04645	0.060359	0.067117	0.08
FARS	0.029682	0.03857	0.042888	0.05112
HARS	0.016006	0.020799	0.023127	0.027567
NARS	0.034669	0.04505	0.050094	0.059709
ACT	0.000152	0.000198	0.00022	0.000263
MYOHC1-2	0	0	0	0
MYOHC2a	0.000305	0.000396	0.000441	0.000525
MYOHC2b	0	0	0	0
MYOLCk	0.000305	0.000396	0.000441	0.000525
MYOLCp	0.000305	0.000396	0.000441	0.000525
MYO1-2x	0	0	0	0
MYO2a	0.000152	0.000198	0.00022	0.000263
MYO2x	0	0	0	0
MYO2b	0	0	0	0
TMN1	0	0	0	0
TMN2	4.36E-05	5.66E-05	6.29E-05	7.5E-05

DIM-TMN1	0	0	0	0
DIM-TMN2	2.18E-05	2.83E-05	3.15E-05	3.75E-05
TPNC1	0	0	0	0
TPNC2	2.18E-05	2.83E-05	3.15E-05	3.75E-05
TPNI1	0	0	0	0
TPNI2	2.18E-05	2.83E-05	3.15E-05	3.75E-05
TPNT1	0	0	0	0
TPNT2	2.18E-05	2.83E-05	3.15E-05	3.75E-05
TPN1	0	0	0	0
TPN2	2.18E-05	2.83E-05	3.15E-05	3.75E-05
CONTR-COI	0	0	0	0
CONTR-COI	2.18E-05	2.83E-05	3.15E-05	3.75E-05
CONTR-COI	0	0	0	0
CONTR-COI	0	0	0	0
Str_CONTR	0	0	0	0
Str_CONTR	2.18E-05	2.83E-05	3.15E-05	3.75E-05
Str_CONT-(	0	0	0	0
Str_CONTR	0	0	0	0
CKM	0	0	0	-42.3428
Spont	0	0	0	42.34276
Nut_cr	0	0	0	0
ATP2UTP	0	0	0	0
UDP2ADP	0	0	0	0
PGM1	0	0	0	0
UGP2	0	0	0	0
mGYG	0	0	0	0
mGYS1	0	0	0	0
G6PD	0	0	0.00001	0
PGLS	0	0	0.00001	0
PGD	0	0	0	0
PGD	0	0	0.00001	0
RPE	0	0	6.67E-06	0
RPIA	0	0	3.33E-06	0
TKT1	0	0	3.33E-06	0
TKT2	0	0	-3.33E-06	0
TALDO	0	0	3.33E-06	0
RBSK	0	0	0	0
GCL	0	0	0	0
GSS	0	0	0	0
glutathxpo	0	0	0	0
GSR	0	0	0	0
GSR	0	0	0	0
GlutathLea	0	0	0	0
GlutathLea	0	0	0	0
Recycle_na	0	0	0.00001	0
fa-c6 leak	0	0	0	0
acetyLeak(	0	0	0	0.00039

4	5	6	7	7-control
**Arg-Ile-Leu-Met**	**Arg-Ile-Leu-Met-Phe**	**Arg-Ile-Leu-Lys-Met-Pl**	**Arg-Ile-Leu-Lys-Met-Pl**	7-
0	0	0	0	0
-5E-06	-0.01816	-0.0145	-0.01311	-0.01311
-5E-06	-0.01816	-0.0145	-0.01311	-0.01311
0	0	0	0	0
0	0	0	0	0
-0.125	-0.125	-0.125	-0.125	-0.125
0	0	0	0	0
0	0	0	0	0
0	0	0	0	0
0	0	0	0	0
0	0	0	0	0
0	0	0	0	0
0	0	0	0	0
0	0	0	0	0
-0.23	-0.23	-0.23	-0.23	-0.23
0	0	0	0	0
0	0	0	0	0
0	0	0	0	0
0	0	0	0	0
0	0	0	0	0
0	0	0	0	0
0	0	0	0	0
0	0	0	0	0
-8.91054	-8.82511	-8.5865	-8.5572	-8.22039
6.283383	6.240209	6.036147	6.384457	6.253486
-999938	-999938	-999939	-999936	-999938
1.5E-05	0.054477	0.043508	0.01311	0.01311
-0.26286	-0.23186	-0.24382	-0.84552	-0.84552
-0.23	-0.23	-0.23	-0.23	-0.23
0	0	0	0	0
0	0	0	0	0
0	0	0	0	0
0	0	0	0	0
0	0	0	0	0
0	0	0	0	0
0.000005	0.018159	0.014503	0.01311	0.01311
0	0	0	0	0
0	0	0	0	0
0.125	0.125	0.125	0.125	0.125
0	0	0	0	0
0	0	0	0	0
0	0	0	0	0
0	0	0	0	0
0	0	0	0	0
0	0	0	0	0

0	0	0	0	0
0	0	0	0	0
0.23	0.23	0.23	0.23	0.23
0	0	0	0	0
0	0	0	0	0
0.23	0.23	0.23	0.23	0.23
0	0	0	0	0
0	0	0	0	0
0	0	0	0	0
0	0	0	0	0
0.000005	0.018159	0.014503	0.01311	0.01311
0.000005	0.018159	0.014503	0.01311	0.01311
0.000005	0.018159	0.014503	0.01311	0.01311
0	0	0	0	0
0.000005	0.018159	0.014503	0.01311	0.01311
-5E-06	-0.01816	-0.0145	0.01311	0.01311
0.00001	0.036318	0.029005	2E-10	2E-10
0.00001	0.036318	0.029005	2E-10	2E-10
0.00001	0.036318	0.006612	-0.11101	-0.06906
0.00001	0.036318	0.006612	-0.11101	-0.06906
999997.1	999996.9	999996.1	999994.3	999994.3
999997.1	999996.9	999996.1	999994.3	999994.3
0	0	0	0	0
0	0	0	0.167679	0.125735
0	0	0	0	0
0.00001	0.00001	0.00001	0.00001	-0.02456
0.00001	0.00001	0.00001	0.167689	0.101178
0	0	0	0.026221	0.026221
0.00001	0.00001	0.00001	0.00001	-0.02456
0.154	0.167812	0.238758	0.563497	0.449905
1E-05	0.036318	0.029005	0	0
0.154	0.167812	0.238758	0.563497	0.449905
0	0	0	0	0
1E-05	0.036318	0.029005	0	0
3.00075	2.971422	2.88374	2.826503	2.826503
3.00075	2.971422	2.88374	2.826503	2.826503
0	0	0	0	0
3.00075	2.971422	2.88374	2.826503	2.826503
2.867813	2.855787	2.771384	2.517637	2.462919
3.026857	3.045978	3.121573	3.389707	3.30068
3.15474	3.139224	3.122487	3.389991	3.300964
3.15474	3.139224	3.122487	3.389991	3.300964
3.15475	3.139234	3.122497	3.390001	3.276408
8.889565	8.844769	8.717974	9.136811	8.726059
8.93151	8.805449	8.455022	7.977581	7.714731
17.82108	17.65022	17.173	17.11439	16.44079
8.910538	8.825109	8.586498	8.557196	8.220395

44.53172	44.14521	43.06396	43.3656	41.60764
999999	999999	999999	999999	999998.1
0.125	0.125	0.125	0.125	0.125
0.125	0.125	0.125	0.046337	0.046337
0.125	0.125	0.125	0.046337	0.046337
0.125	0.125	0.125	0.046337	0.046337
0	0	0	0	0
0	0	0	0	0
0	0	0	0	0
0	0	0	0	0
0.125	0.125	0.125	0.046337	0.046337
0.125	0.125	0.125	0.046337	0.046337
0.125	0.125	0.125	0.046337	0.046337
0.125	0.125	0.125	0.046337	0.046337
0	0	0	0	0
0	0	0	0	0
0	0	0	0	0
0	0	0	0	0
0.355	0.355	0.355	0.276337	0.276337
0.355	0.355	0.355	0.276337	0.276337
0.355	0.355	0.355	0.276337	0.276337
0.355	0.355	0.355	0.276337	0.276337
0.355	0.355	0.355	0.276337	0.276337
0.355	0.355	0.355	0.276337	0.276337
0.355	0.355	0.355	0.276337	0.276337
0.355	0.355	0.355	0.276337	0.276337
0.355	0.355	0.355	0.276337	0.276337
0.355	0.355	0.355	0.276337	0.276337
0.355	0.355	0.355	0.276337	0.276337
0.355	0.355	0.355	0.276337	0.276337
0.355	0.355	0.355	0.276337	0.276337
0.355	0.355	0.355	0.276337	0.276337
0.355	0.355	0.355	0.276337	0.276337
0.355	0.355	0.355	0.276337	0.276337
0.355	0.355	0.355	0.276337	0.276337
0.355	0.355	0.355	0.276337	0.276337
0.355	0.355	0.355	0.276337	0.276337
0.355	0.355	0.355	0.276337	0.276337
0.355	0.355	0.355	0.276337	0.276337
0.355	0.355	0.355	0.276337	0.276337
0.355	0.355	0.355	0.276337	0.276337
0.482883	0.448246	0.355914	0.276621	0.276621
0	0	0	0	0
0	0	0	0.026221	0.026221
0	0	0	0.026221	0.026221
0	0	0	0.026221	0.026221
0	0	0	0.026221	0.026221

0	0	0	0.026221	0.026221
0	0	0	0	0
0	0	0	0	0
0	0	0	0	0
0	0	0	0	0
0	0	0	0	0
0	0	0	0	0
0	0	0	0	0
0	0	0	0	0
0.23	0.23	0.23	0.23	0.23
0	0	0	0	0
0	0	0	0	0
0	0	0	0	0
0	0	0	0	0
0	0	0	0	0
0	0	0	0	0
0	0	0	0	0
0	0	0	0	0
0	0	0	0	0
0.23	0.23	0.23	0.23	0.23
0	0	0	0	0
0	0	0	0	0
0	0	0	0	0
0	0	0	0	0
0	0	0	0	0
0	0	0	0	0
0	0	0	0	0
0	0	0	0	0
0	0	0	0	0
0.23	0.23	0.23	0.23	0.23
0	0	0	0	0
0	0	0	0	0
0	0	0	0	0
0	0	0	0	0
0	0	0	0	0
0	0	0	0	0
0	0	0	0	0
0	0	0	0	0
0	0	0	0	0
0.23	0.23	0.23	0.23	0.23
0	0	0	0	0
0	0	0	0	0
0	0	0	0	0
0	0	0	0	0
0	0	0	0	0
0	0	0	0	0
0	0	0	0	0

0	0	0	0	0
0	0	0	0	0
0	0	0	0	0
0	0	0	0	0
0	0	0	0	0
0	0	0	0	0
0	0	0	0	0
0	0	0	0	0
0	0	0	0	0
-0.0892	-0.09705	-0.13736	-0.2266	-0.18015
-0.03074	-0.03344	-0.04733	-0.07808	-0.06208
-0.2266	-0.2383	-0.384	-1.078	-1.03029
-0.29136	-0.2711	-0.25264	-0.41556	-0.33043
-0.1728	-0.188	-0.26609	-0.43897	-0.34898
-0.04801	-0.05223	-0.07393	-0.12196	-0.09696
-0.057	-0.09832	-0.13916	-0.22958	-0.18251
-0.09092	-0.09891	-0.14	-0.23096	-0.18361
-0.00949	-0.01033	-0.01462	-0.02412	-0.01917
-0.233	-0.233	-0.233	-0.233	-0.15649
-0.333	-0.333	-0.333	-0.333	0
0	0	0	0	0.034669
-0.003	-0.003	-0.003	-0.003	-0.003
-0.01974	-0.02148	-0.0304	-0.05014	-0.03986
-0.024	-0.024	-0.024	-0.024	0
-0.03864	-0.08793	-0.25552	-0.42275	-0.3707
-0.09054	-0.0985	-0.13942	-0.23	-0.18285
-0.168	-0.168	-0.168	-0.168	0
-0.08857	-0.09637	-0.114	-0.114	-0.10982
-0.03337	0	0	0	0
0.41481	0.376682	0.352017	0.872637	0.838329
0.151954	0.144824	0.108198	0.027115	-0.00719
0.151954	0.144824	0.108198	0.027115	-0.00719
0.151954	0.144824	0.108198	0.027115	-0.00719
0.151954	0.144824	0.108198	0.027115	-0.00719
0.151954	0.144824	0.108198	0.027115	-0.00719
0.151954	0.144824	0.108198	0.027115	-0.00719
0.151954	0.144824	0.108198	0.027115	-0.00719
0.151954	0.144824	0.108198	0.027115	-0.00719
0.151954	0.144824	0.108198	0.027115	-0.00719
0.286927	0.283436	0.351103	0.872353	0.838045
0.286927	0.283436	0.351103	0.872353	0.838045
0.286927	0.283436	0.351103	0.872353	0.838045
0.134973	0.138613	0.242905	0.845238	0.845238
0.134973	0.138613	0.242905	0.845238	0.845238
0.134973	0.138613	0.242905	0.845238	0.845238
0.134973	0.138613	0.242905	0.845238	0.845238
0.134973	0.138613	0.242905	0.845238	0.845238

0.134973	0.138613	0.242905	0.845238	0.845238
0.134973	0.138613	0.242905	0.845238	0.845238
0.127883	0.093246	0.000914	0.000284	0.000284
0.127883	0.093246	0.000914	0.000284	0.000284
0.127883	0.093246	0.000914	0.000284	0.000284
0.127883	0.093246	0.000914	0.000284	0.000284
0.127883	0.093246	0.000914	0.000284	0.000284
0.127883	0.093246	0.000914	0.000284	0.000284
0.127883	0.093246	0.000914	0.000284	0.000284
0.127883	0.093246	0.000914	0.000284	0.000284
0.127883	0.093246	0.000914	0.000284	0.000284
0	0	0	0	0
0.127883	0.093246	0.000914	0.000284	0.000284
0.127883	0.093246	0.000914	0.000284	0.000284
0	0	0	0	0
0	0	-0.02239	0.056672	0.056672
0.179606	0.166111	0.074397	0	-0.25312
0	0	0	0	0
0	0	0	0	0
0	0	0	0	0
0	0	0	0	0
0	0	0	0	0
0	0	0	0	0
0	0	0	0	0
0	0	0	0	0
0	0	0	0	0
0	0	0	0	0
0	0	0	0	0
0	0	0	0	0
0.1133	0.108488	0.083768	0.029044	-0.11047
3.241933	3.173156	2.968422	2.855831	2.716316
0.1133	0.108488	0.083768	0.029044	-0.11047
0	0	0	0	0
0	0	0	0	0
0	0	0	0	0
0	0	0	0	0
0	0	0	0	0
0	0	0	0	0
0	0	0	0	0
0	0	0	0	0
0	0	0	0	0
0	0	0	0	0
0	0	0	0	0
0	0	0	0	0
0	0	0	0	0
0	0	0	0	0
0	0	0	0	0

0	0	0	0	0
0	0	0	0	0
0.066577	0.072434	0.102521	0.169127	0.169127
0	0	0.022394	0.111007	0.069062
0	0	0.022394	0.111007	0.069062
0	0	0.022394	0.111007	0.069062
0	0	0	0	0
0	0	0	0	0
0	0	0	0	0
3.128633	3.064668	2.884654	2.826787	2.826787
0	0.036308	0.051389	0.084776	0.067398
0	0.036308	0.051389	0.084776	0.067398
0	0.036308	0.051389	0.084776	0.067398
0.400227	0.391924	0.434871	0.901397	0.727573
0	0	0	0	0
0.033372	0.036308	0.051389	0.084776	0.067398
0.009493	0.010328	0.014618	0.024115	0.019172
0.090916	0.098914	0.14	0.230956	0.183613
0.163472	0.177854	0.25173	0.415275	0.330149
0.091627	0.099687	0.141095	0.232762	0.185049
0.172798	0.188	0.26609	0.438965	0.348983
0.153394	0.166889	0.23621	0.389672	0.309794
0.081046	0.088176	0.124802	0.205885	0.163681
0.048009	0.052232	0.073928	0.121958	0.096958
0.088574	0.096366	0.136394	0.225007	0.178883
0.090414	0.098368	0.139227	0.229681	0.182599
0.090539	0.098504	0.13942	0.23	0.182853
0.0547	0.059512	0.084232	0.138956	0.110472
0.019739	0.021475	0.030396	0.050143	0.039864
0.20893	0.227311	0.32173	0.530753	0.421955
0.099949	0.108742	0.15391	0.253903	0.201856
0.089201	0.097048	0.13736	0.2266	0.18015
0.057	0.062015	0.087774	0.144799	0.115117
0.030737	0.033441	0.047332	0.078083	0.062077
0.066577	0.072434	0.102521	0.169127	0.134458
0.000293	0.000318	0.000451	0.000744	0.000591
0	0	0	0	0
0.000585	0.000637	0.000902	0.001487	0.001182
0	0	0	0	0
0.000585	0.000637	0.000902	0.001487	0.001182
0.000585	0.000637	0.000902	0.001487	0.001182
0	0	0	0	0
0.000293	0.000318	0.000451	0.000744	0.000591
0	0	0	0	0
0	0	0	0	0
0	0	0	0	0
8.36E-05	9.1E-05	0.000129	0.000212	0.000169

0	0	0	0	0
4.18E-05	4.55E-05	6.44E-05	0.000106	8.45E-05
0	0	0	0	0
4.18E-05	4.55E-05	6.44E-05	0.000106	8.45E-05
0	0	0	0	0
4.18E-05	4.55E-05	6.44E-05	0.000106	8.45E-05
0	0	0	0	0
4.18E-05	4.55E-05	6.44E-05	0.000106	8.45E-05
0	0	0	0	0
4.18E-05	4.55E-05	6.44E-05	0.000106	8.45E-05
0	0	0	0	0
4.18E-05	4.55E-05	6.44E-05	0.000106	8.45E-05
0	0	0	0	0
0	0	0	0	0
0	0	0	0	0
4.18E-05	4.55E-05	6.44E-05	0.000106	8.45E-05
0	0	0	0	0
0	0	0	0	0
-43.1133	-42.5429	-40.0214	-36.5721	-36.5721
43.11334	42.54293	40.02138	36.57209	36.57209
0	0	0	0	0
0	0	0	0	0
0	0	0	0	0
0	0	0	0	0
0	0	0	0	0
0	0	0	0	0
0	0	0	0	0
0	0	0	0	0
0	0	0	0	0
0	0	0	0	0
0	0	0	0	0
0	0	0	0	0
0	0	0	0	0
0	0	0	0	0
0	0	0	0	0
0	0	0	0	0
0	0	0	0	0
0	0	0	0	0
0	0	0	0	0
0	0	0	0	0
0	0	0	0	0
0	0	0	0	0
0	0	0	0	0
0	0	0	0	0
0	0	0	0	0
0	0	0	0	0
0.127883	0.093246	0.000914	0.000284	0.000284

	actin	myoHC1-2a	myoHC2a	myoHC2b	myoLCk	myoLCp	tmn1	tmn2
arg	18	104	106	100	24	7	14	20
his	9	37	36	40	10	1	2	2
ile	30	103	104	94	24	10	12	12
leu	26	191	200	194	50	9	34	28
lys	19	208	209	212	52	16	38	25
met	17	55	46	56	18	7	8	5
phe	12	56	58	61	20	12	1	1
thr	27	101	98	101	30	11	8	11
trp	4	9	9	9	4	1	0	0
val	21	84	84	86	31	10	7	10
ala	29	171	172	168	56	12	38	28
asn	12	76	75	77	24	7	7	6
asp	22	98	93	94	30	15	19	14
cys	6	17	18	17	10	2	1	3
glu	28	261	265	263	48	15	61	54
gln	11	130	131	127	23	7	15	15
gly	28	72	77	73	48	13	3	4
pro	19	31	30	32	47	6	0	0
ser	23	94	92	97	39	6	11	7
tyr	16	41	38	38	8	2	6	3

tpnc1	tpnc2	tpni1	tpni2	tpnt1	tpnt2	Type 1
4	7	19	17	29	25	2096
0	1	4	5	5	4	748
9	10	4	5	10	10	2175
13	9	22	17	19	19	3804
13	9	24	23	32	39	4142
11	11	8	10	7	4	1281
9	10	2	3	6	4	1335
7	6	6	4	4	7	2210
0	0	2	1	3	2	229
8	7	13	8	11	6	1943
7	12	15	13	20	24	3667
5	3	3	5	3	4	1607
23	19	10	11	12	18	2239
2	1	3	3	1	0	456
23	26	22	25	54	49	4953
5	5	4	8	16	16	2372
12	13	6	7	12	9	2094
2	2	7	5	18	6	1336
5	7	11	10	11	8	2156
3	2	2	2	5	4	848

Type 2a	Type 2x	Type 2b
2133	2105	2049
735	749	791
2191	2177	2051
3909	3783	3825
4132	4118	4174
1148	1274	1288
1363	1335	1405
2174	2216	2216
227	227	227
1938	1938	1966
3668	3654	3612
1592	1606	1620
2162	2232	2176
472	458	458
4996	4940	4968
2390	2376	2334
2165	2095	2109
1308	1322	1336
2118	2146	2188
798	840	798

AA	Available (flux)	Normalized to 1: available/control flux
ARG	0.08	3673.6
HIS	0.082	3765.44
ILE	0.062	2847.04
LEU	0.123	5648.16
LYS	0.188	8632.96
MET	0.025	1148
PHE	0.057	2617.44
THR	0.14	6428.8
TRP	0.044	2020.48
VAL	0.233	10699.36
ALA	0.333	15291.36
ASN	0.041	1882.72
ASP	0.003	137.76
CYS	0.052	2387.84
GLU	0.024	1102.08
GLN	0.586	26909.12
GLY	0.23	10561.6
PRO	0.168	7714.56
SER	0.114	5234.88
TYR	0.059	2709.28

mmol

6.02E+23

Initial Orde	Essentialit	CE		Needed	Available	Difference	Expected	Actual
6	1	1	**MET**	1148	1148	0	1	1
3	1	1	**ILE**	2191	2847	656	2	2
7	1	1	**PHE**	1363	2617	1254	3	5
4	1	1	**LEU**	3909	5648	1739	4	3
9	1	1	**TRP**	227	2020	1793	5	n/a
8	1	1	**THR**	2174	6429	4255	6	7
5	1	1	**LYS**	4132	8633	4501	7	6
10	1	1	**VAL**	1938	10699	8761	8	n/a
1	2	1	**ARG**	2133	3674	1541	9	4
2	2	1	**HIS**	735	3765	3030	10	n/a
15	3	2	**GLU**	4996	1102	-3894	11	n/a
13	3	2	**ASP**	2162	138	-2024	12	n/a
12	3	2	**ASN**	1592	1883	291	13	n/a
20	3	2	**TYR**	798	2709	1911	14	n/a
14	3	2	**CYS**	472	2388	1916	15	n/a
19	3	2	**SER**	2118	5235	3117	16	n/a
18	3	2	**PRO**	1308	7715	6407	17	n/a
17	3	2	**GLY**	2165	10562	8397	18	n/a
11	3	2	**ALA**	3668	15291	11623	19	n/a
16	3	2	**GLN**	2390	26909	24519	20	n/a

**Essentiality**	**Expect on I**	**Expect on CE**
e	1	1
e	2	2
e	3	3
e	4	5
e	5	6
e	6	8
e	7	9
e	8	10
ce	9	4
ce	10	7
ne	11	11
ne	12	12
ne	13	13
ne	14	14
ne	15	15
ne	16	16
ne	17	17
ne	18	18
ne	19	19
ne	20	20
