# Supplementary material for: To Supplement or Not to Supplement: A Metabolic Network Framework for Human Nutritional Supplements
Source: PLoS One. 2013 Aug 5;8(8):e68751. doi: 10.1371/journal.pone.0068751 (PMC3740736; doi:10.1371/journal.pone.0068751)
Supplement: File S5 — Results: Post-absorptive. (PDF) [file pone.0068751.s006.pdf]

Fasting		Type 2a	Single		Double
	Available	Control	Methionin	Difference	Met-Phe
Prot Synth	n/a	2.61E-05	4.99E-05		5.39E-05
ARG	0.115	-0.056	-0.106		-0.115
HIS	0.118	-0.019	-0.037		-0.040
ILE	0.128	-0.057	-0.128		-0.128
LEU	0.271	-0.102	-0.195		-0.211
LYS	0.294	-0.108	-0.206		-0.223
MET	0.030	-0.030	-0.057		-0.062
PHE	0.068	-0.036	-0.068		-0.073
THR	0.182	-0.057	-0.108		-0.117
TRP	0.044	-0.006	-0.011		-0.012
VAL	0.349	-0.051	-0.145		-0.206
ALA	0.309	-0.309	-0.309		-0.309
ASN	0.045	-0.042	-0.045		-0.045
ASP	0.067	0.000	0.000		-0.067
CYS	0.093	-0.012	-0.024		-0.025
GLU	0.152	-0.152	-0.152		-0.152
GLN	0.541	-0.063	-0.154		-0.541
GLY	0.281	-0.057	-0.108		-0.117
PRO	0.299	-0.161	-0.299		-0.299
SER	0.175	0.000	-0.010		-0.114
TYR	0.092	-0.021	-0.040		-0.043

	Available					
	0	1	2 HR	3	4	0-2 hr diff
ARG	0.061	0.098	0.120	0.106	0.092	0.059
HIS	0.092	0.105	0.155	0.105	0.106	0.063
ILE	0.067	0.105	0.144	0.155	0.137	0.077
LEU	0.14	0.205	0.271	0.282	0.247	0.131
LYS	0.189	0.277	0.328	0.281	0.259	0.139
MET						
PHE	0.049	0.062	0.068	0.069	0.06	0.019
THR	0.149	0.185	0.214	0.212	0.19	0.065
TRP						
VAL	0.238	0.3	0.359	0.395	0.366	0.121
ALA	0.271	0.34	0.345	0.324	0.289	0.074
ASN						
ASP	0.115	0.114	0.114	0.116	0.112	-0.001
CYS						
GLU	0.145	0.148	0.152	0.148	0.149	0.007
GLN	0.658	0.662	0.676	0.653	0.66	0.018
GLY	0.352	0.387	0.377	0.367	0.345	0.025
PRO	0.179	0.223	0.231	0.227	0.211	0.052
SER	0.118	0.209	0.217	0.197	0.183	0.099
TYR	0.063	0.077	0.092	0.093	0.082	0.029

0.061

	Basal						Postabsorptive
	Cynober	Aoki	Pozefsky	Tessari	AVG	STD	Aoki
**ARG**	0.08	0.061	0.075	0.073	**0.072**	0.008	0.120
**HIS**	0.082	0.092	0.076	0.06	**0.078**	0.013	0.155
**ILE**	0.062	0.067	0.056	0.053	**0.060**	0.006	0.144
**LEU**	0.123	0.14	0.12		**0.128**	0.011	0.271
**LYS**	0.188	0.189	0.172	0.144	**0.173**	0.021	0.328
**MET**	0.025		0.021	0.014	**0.020**	0.006	
**PHE**	0.057	0.049	0.049		**0.052**	0.005	0.068
**THR**	0.14	0.149	0.135	0.107	**0.133**	0.018	0.214
**TRP**	0.044		0.038		**0.041**	0.004	
**VAL**	0.233	0.238	0.238	0.211	**0.230**	0.013	0.359
**ALA**	0.333	0.271	0.231	0.24	**0.269**	0.046	0.345
**ASN**	0.041			0.025	**0.033**	0.011	
**ASP**	0.003	0.115		0.016	**0.045**	0.061	0.114
**CYS**	0.052		0.086		**0.069**	0.024	
**GLU**	0.024	0.145		0.152	**0.107**	0.072	0.152
**GLN**	0.586	0.658		0.366	**0.537**	0.152	0.676
**GLY**	0.23	0.352	0.179	0.191	**0.238**	0.079	0.377
**PRO**	0.168	0.179	0.178	0.215	**0.185**	0.021	0.231
**SER**	0.114	0.118	0.107	0.109	**0.112**	0.005	0.217
**TYR**	0.059	0.063	0.054	0.047	**0.056**	0.007	0.092

	Triple		Quadruple	
**Difference**	**Arg-Met-Phe-**	**Difference**	**Arg-Ile-Met-Phe**	**Difference**
	5.84E-05		6.93E-05	
	-0.1246		-0.148	
	-0.0429		-0.051	
	-0.1280		-0.331	
	-0.2710		-0.271	
	-0.2414		-0.286	
	-0.0671		-0.080	
	-0.0796		-0.094	
	-0.1270		-0.151	
	-0.0133		-0.016	
	-0.3490		-0.134	
	-0.3090		-0.309	
	0.3510		-0.045	
	-0.0670		0.000	
	-0.0276		-0.033	
	-0.1520		-0.152	
	-0.5410		-0.231	
	-0.1265		-0.150	
	-0.2990		-0.299	
	0.1875		-0.103	
	-0.0466		-0.055	

	Mixed Meal (Tessari et al)	
Cynober - Aoki	Basal	Meal
0.054	0.073	0.11
0.026	0.06	0.081
0.061	0.053	0.112
0.131		
0.105	0.144	0.26
	0.014	0.03
0.019		
0.0325	0.107	0.149
0.111	0.211	0.339
0.0375	0.24	0.272
	0.025	0.045
-0.048	0.016	0.02
0.007	0.152	0.152
-0.1175	0.366	0.405
-0.071	0.191	0.185
0.12	0.215	0.367
0.057	0.109	0.133
0.0285	0.047	0.091

0.035

(post insulin)

**Posefsky**	**Tessari**	**AVG**	**STD**	**Diff b/n AVG**	**%Dif**
	0.11	**0.115**	0.007	0.043	59%
	0.081	**0.118**	0.052	0.041	52%
	0.112	**0.128**	0.023	0.069	115%
		**0.271**		0.143	112%
	0.26	**0.294**	0.048	0.121	70%
	0.03	**0.030**		0.010	50%
		**0.068**		0.016	32%
	0.149	**0.182**	0.046	0.049	37%
		**0.044**			
	0.339	**0.349**	0.014	0.119	52%
	0.272	**0.309**	0.052	0.040	15%
	0.045	**0.045**		0.012	36%
	0.02	**0.067**	0.066	0.022	50%
0.093		**0.093**			
	0.152	**0.152**	0.000	0.045	42%
	0.405	**0.541**	0.192	0.004	1%
	0.185	**0.281**	0.136	0.043	18%
	0.367	**0.299**	0.096	0.114	62%
	0.133	**0.175**	0.059	0.063	56%
	0.091	**0.092**	0.001	0.036	64%

Quintiple		Hextuple		Heptuple
**Arg-Ile-Leu-Met-Phe**	**Difference**	**Arg-Ile-Leu-Lys-Met-Phe**	**Difference**	**Arg-Ile-Leu**
7.12E-05		8.37E-05		0.00013
-0.152		-0.1786		-0.277
-0.052		-0.0615		-0.095
-0.347		-0.4546		-1.035
-0.278		-0.3272		-0.507
-0.294		-0.3459		-0.536
-0.082		-0.0961		-0.149
-0.097		-0.1141		-0.280
-0.155		-0.1820		-0.282
-0.016		-0.0190		-0.029
-0.138		-0.1622		-0.349
-0.309		-0.3090		-0.309
-0.045		-0.0450		-0.045
0.000		0.0000		-0.067
-0.034		-0.0395		-0.061
-0.152		-0.1520		-0.152
-0.238		-0.2884		-0.472
-0.154		-0.1812		-0.281
-0.299		-0.2990		-0.299
-0.111		-0.1639		-0.175
-0.057		-0.0668		0.000

avg from Cynober

-Lys-Met-Phe-Thr

		1	2	3
Flux	Control	Met	**Met-Phe**	**Arg-Met-Phe**
Obj_atp	0	0	0	0
Ex_ins	0	0	0	0
Ex_gluc	0	0	0	0
Ex_lac	0	0	0	0
Ex_pyr	0	0	0	0
Ex_pal	0	0	0	0
Ex_prop	0	0	0	0
Ex_but	0	0	0	0
Ex_5c	0	0	0	0
Ex_6c	0	0	0	0
Ex_8c	0	0	0	0
Ex_9c	0	0	0	0
Ex_10c	0	0	0	0
Ex_12c	0	0	0	0
Ex_14c	0	0	0	0
Ex_18c	0	0	0	0
Ex_glyc	0	0	0	0
Ex_tag	0	0	0	0
Ex_dag	0	0	0	0
Ex_mag	0	0	0	0
Ex_akg	0	0	0	0
Ex_oaa	0	0	0	0
Ex_rib	0	0	0	0
Nut_o2	-0.3776	-0.75931	-0.8275	-0.90385
Nut_co2	0.240598	0.371894	0.374225	0.376835
Nut_h2o	2.620854	4.991492	5.389164	5.834404
Nut_p	0	0	0	0
Nut_hco3	0	-0.01869	-0.00987	0
Nut_coa	0	0	0	0
Nut_carn	0	0	0	0
Nut_dhf	0	0	0	0
LPL	0	0	0	0
DAGL	0	0	0	0
12DAG	0	0	0	0
MAGL	0	0	0	0
GLUT4	0	0	0	0
MCT4	0	0	0	0
MCT_pyr	0	0	0	0
FAT/CD36	0	0	0	0
FATprop	0	0	0	0
FATbut	0	0	0	0
FAT5c	0	0	0	0
FAT6c	0	0	0	0
FAT8c	0	0	0	0
FAT9c	0	0	0	0

FAT10c	0	0	0	0
FAT12c	0	0	0	0
FAT14c	0	0	0	0
FAT18c	0	0	0	0
Alc_glyc	0	0	0	0
COA	0	0	0	0
SLC22A5/O	0	0	0	0
Xport_rib	0	0	0	0
Dr_akg	0	0	0	0
Dr_oaa	0	0	0	0
Hex	0	0	0	0
PGI	0	0	0	0
PFK	0	0	0	0
FBP	0	0	0	0
Ald	0	0	0	0
TPI	0	0	0	0
D3PDeh	0	0	0	0
PGK	0	0	0	0
PGM	-0.05535	-0.09611	-0.09854	-0.10126
Eno	-0.05535	-0.09611	-0.09854	-0.10126
PyrK	0.036702	0	0	0
PEPSynth	0	0	0	0
PEPCarbK	0	0	0	0
PEPCarbl	0.09205	0.096113	0.098542	0.101262
LacDeh	0	0	0	0
MalDehC(n	0.055348	0.096113	0.098542	0.101262
ASTC	0.147398	0.192225	0.197084	0.202523
G3PDehC	0	0	0	0
Mal-AKGEX	0.055348	0.096113	0.098542	0.101262
Glu-AspEx	0.203897	0.334512	0.354479	0.376835
MCT	0	0	0	0
ASTM	0.203897	0.334512	0.354479	0.376835
G3PDehM	0	0	0	0
PyrSynth/P	0	0	0	0
CitSynth	0	0.018691	0.009873	0
Acon	0	0.018691	0.009873	0
IsoDeh	0	0	0	0
IsoDehnad	0	0.018691	0.009873	0
AKGDeh	0.148548	0.18961	0.161827	0.130721
SunCoaSyn	0.148548	0.257091	0.26581	0.275573
SucDeh(I)	0.148548	0.257091	0.26581	0.275573
Fum	0.148548	0.257091	0.26581	0.275573
MalDehM	0.203897	0.353203	0.364352	0.376835
NADHDeh(	0.479551	0.960308	1.056736	1.164698
ETFUO	0.275654	0.558315	0.598273	0.643011
UCytC(III)	0.755204	1.518623	1.655009	1.807709
CytCO(IV)	0.377602	0.759312	0.827505	0.903855

ATPSynth(\	1.989959	3.997555	4.366755	4.780116
AMP_ADPs	1.087605	2.110795	2.284707	2.479422
PalTK	0	0	0	0
CPT1B/CHK	0	0	0	0
SLC25A20/	0	0	0	0
CPT2	0	0	0	0
CRAT(mit)	0	0	0	0
CRAT Trans	0	0	0	0
CRAT(cyt)	0	0	0	0
Acetyl-coaL	0	0	0	0
AcylCoADe	0	0	0	0
EnoCoADel	0	0	0	0
3HA-CoADe	0	0	0	0
AcylCoAAT	0	0	0	0
AcylCoADe	0	0	0	0
EnoCoADel	0	0	0	0
3HA-CoADe	0	0	0	0
AcylCoAAT	0	0	0	0
AcylCoADe	0	0	0	0
EnoCoADel	0	0	0	0
3HA-CoADe	0	0	0	0
AcylCoAAT	0	0	0	0
AcylCoADe	0	0	0	0
EnoCoADel	0	0	0	0
3HA-CoADe	0	0	0	0
AcylCoAAT	0	0	0	0
AcylCoADe	0	0	0	0
EnoCoADel	0	0	0	0
3HA-CoADe	0	0	0	0
AcylCoAAT	0	0	0	0
AcylCoADe	0	0	0	0
EnoCoADel	0	0	0	0
3HA-CoADe	0	0	0	0
AcylCoAAT	0	0	0	0
AcylCoADe	0	0	0	0
EnoCoADel	0	0	0	0
3HA-CoADe	0	0	0	0
AcylCoAAT	0	0	0	0
ButyrlCoAD	0	0	0	0
EnoCoADel	0	0	0	0
3HA-CoADe	0	0	0	0
AcylCoAAT	0	0	0	0
GK	0	0	0	0
GPAT4	0	0	0	0
ABHD5	0	0	0	0
DAGZ/DAG	0	0	0	0
DGAT1	0	0	0	0

St_TAG	0	0	0	0
ACAS2	0	0	0	0
ACSM-But	0	0	0	0
ASCM-5c	0	0	0	0
ASCM-6c	0	0	0	0
ACSM-8c	0	0	0	0
ACSM-9c	0	0	0	0
ACSM-10c	0	0	0	0
ACSM-12c	0	0	0	0
ACSM-14c	0	0	0	0
ACSM-18c	0	0	0	0
CPT1B/CHK	0	0	0	0
CPT1B/CHK	0	0	0	0
CPT1B/CHK	0	0	0	0
CPT1B/CHK	0	0	0	0
CPT1B/CHK	0	0	0	0
CPT1B/CHK	0	0	0	0
CPT1B/CHK	0	0	0	0
CPT1B/CHK	0	0	0	0
CPT1B/CHK	0	0	0	0
CPT1B/CHK	0	0	0	0
SLC25A20/	0	0	0	0
SLC25A20/	0	0	0	0
SLC25A20/	0	0	0	0
SLC25A20/	0	0	0	0
SLC25A20/	0	0	0	0
SLC25A20/	0	0	0	0
SLC25A20/	0	0	0	0
SLC25A20/	0	0	0	0
SLC25A20/	0	0	0	0
SLC25A20/	0	0	0	0
CPT2	0	0	0	0
CPT2	0	0	0	0
CPT2	0	0	0	0
CPT2	0	0	0	0
CPT2	0	0	0	0
CPT2	0	0	0	0
CPT2	0	0	0	0
CPT2	0	0	0	0
CPT2	0	0	0	0
CPT2	0	0	0	0
AcylCoADe	0	0	0	0
EnoCoADe	0	0	0	0
3HA-CoAD	0	0	0	0
AcylCoAAT	0	0	0	0
AcylCoADe	0	0	0	0
EnoCoADe	0	0	0	0

3HA-CoADe	0	0	0	0
AcylCoAAT-	0	0	0	0
AcylCoADe	0	0	0	0
EnoCoADel	0	0	0	0
3HA-CoADe	0	0	0	0
AcylCoAAT-	0	0	0	0
AKR1A1	0	0	0	0
ALDH3A2	0	0	0	0
GLYCTK	0	0	0	0
EAA_arg	-0.05574	-0.10642	-0.115	-0.12461
EAA_his	-0.01921	-0.03667	-0.03963	-0.04294
EAA_ile	-0.05726	-0.128	-0.128	-0.128
EAA_leu	-0.10215	-0.19502	-0.21075	-0.22837
EAA_lys	-0.10798	-0.20615	-0.22278	-0.24139
EAA_met	-0.03	-0.05727	-0.06189	-0.06707
EAA_phe	-0.03562	-0.068	-0.07349	-0.07963
EAA_thr	-0.05681	-0.10846	-0.11721	-0.12701
EAA_trp	-0.00593	-0.01133	-0.01224	-0.01326
EAA_val	-0.05064	-0.14548	-0.1986	-0.25807
NEAA_ala	-0.309	-0.309	-0.309	-0.309
NEAA_asn	-0.0416	-0.045	-0.045	-0.045
NEAA_asp	0	0	0	0
NEAA_cys	-0.01233	-0.02355	-0.02545	-0.02757
NEAA_glu	-0.152	-0.152	-0.152	-0.152
NEAA_gln	-0.06246	-0.15366	-0.16969	-0.18763
NEAA_gly	-0.05658	-0.10801	-0.11673	-0.12648
NEAA_pro	-0.16129	-0.299	-0.299	-0.299
NEAA_ser	0	-0.00955	-0.01565	-0.02247
NEAA_tyr	-0.02085	-0.03981	-0.04302	-0.04662
DLDH	0	0.067481	0.103983	0.144852
MT_val	0	0.04879	0.09411	0.144852
BCAT2-val	0	0.04879	0.09411	0.144852
BCKDHB-va	0	0.04879	0.09411	0.144852
BCKADE2-v	0	0.04879	0.09411	0.144852
ACADSB	0	0.04879	0.09411	0.144852
ECHS1	0	0.04879	0.09411	0.144852
HIBCH	0	0.04879	0.09411	0.144852
HIBADH	0	0.04879	0.09411	0.144852
ALDH6A1	0	0.04879	0.09411	0.144852
PCCB	0	0.067481	0.103983	0.144852
MCEE	0	0.067481	0.103983	0.144852
MUT	0	0.067481	0.103983	0.144852
MT_ile	0	0.018691	0.009873	0
BCAT-ile	0	0.018691	0.009873	0
BCKDHB-ile	0	0.018691	0.009873	0
BCKADE2-il	0	0.018691	0.009873	0
ECHS1	0	0.018691	0.009873	0

HSD17B10	0	0.018691	0.009873	0
ACAT1	0	0.018691	0.009873	0
MT_leu	0	0	0	0
BCAT2-leu	0	0	0	0
BCKDHB-le	0	0	0	0
BCKADE2-l	0	0	0	0
IVD	0	0	0	0
MCC1,MCC	0	0	0	0
MGCA	0	0	0	0
HMGCL	0	0	0	0
OXCT	0	0	0	0
HMGCS2	0	0	0	0
GluDeh	0	0	0	0
GlnSynth	0	0	0	0
GLN_Elim	0	0	0	0
ALT	0.036702	0	0	0
ALA_Elim	0.249848	0.126004	0.111241	0.094712
SDS	0	0	0	0
CBS	0	0	0	0
CTH	0	0	0	0
ILVBL	0	0	0	0
2a2hButLe	0	0	0	0
MT_lys	0	0	0	0
AASS	0	0	0	0
AASS	0	0	0	0
aAASADeh	0	0	0	0
KATII	0	0	0	0
lysdeg	0	0	0	0
GCDH	0	0	0	0
MT_pro	0.127106	0.233744	0.22848	0.222586
PRODH	0.127106	0.252435	0.238353	0.222586
ALDH4a1	0.127106	0.233744	0.22848	0.222586
GATM	0	0	0	0
Dr_orn	0	0	0	0
GAMT	0	0	0	0
Dr_cr	0	0	0	0
AdoHcyase	0	0	0	0
MTR	0	0	0	0
AdoMet	0	0	0	0
SHMT	0	0	0	0
MTHFR	0	0	0	0
DHFR	0	0	0	0
HAL	0	0	0	0
FLJ31300	0	0	0	0
MGC35366	0	0	0	0
FTCD1	0	0	0	0
FTCD2	0	0	0	0

FT	0	0	0	0
ASNase	0	0	0	0
ASNSynth	0	0.034425	0.040832	0.048006
PHGDH	0.055348	0.096113	0.098542	0.101262
PSA	0.055348	0.096113	0.098542	0.101262
PSPH	0.055348	0.096113	0.098542	0.101262
P5CS	0	0	0	0
PYCS	0	0	0	0
Spont	0	0	0	0
PYRCR1	0	0.018691	0.009873	0
PAG	0	0	0	0
PCBD	0	0	0	0
QDPR	0	0	0	0
MT_glu	0.127106	0.301225	0.332463	0.367438
tRNA	0	0	0	0
YARS	0.020854	0.039812	0.043024	0.04662
WARS	0.005932	0.011325	0.012239	0.013262
TARS	0.056812	0.108461	0.117211	0.127007
LARS	0.102152	0.19502	0.210752	0.228367
IARS	0.057256	0.109309	0.118127	0.128
KARS	0.107979	0.206145	0.222775	0.241395
AARS	0.095854	0.182996	0.197759	0.214288
VARS	0.050645	0.096687	0.104487	0.11322
MARS	0.03	0.057274	0.061894	0.067067
SARS	0.055348	0.105667	0.114191	0.123735
DARS	0.056498	0.107862	0.116564	0.126306
GARS	0.056577	0.108012	0.116725	0.126481
PARS	0.034181	0.065256	0.07052	0.076414
CARS	0.012334	0.023548	0.025448	0.027575
EPRS	0.130557	0.24925	0.269358	0.29187
QARS	0.062456	0.119237	0.128856	0.139626
RARS	0.05574	0.106415	0.115	0.124612
FARS	0.035618	0.068	0.073486	0.079628
HARS	0.019207	0.036669	0.039627	0.042939
NARS	0.041603	0.079425	0.085832	0.093006
ACT	0.000183	0.000349	0.000377	0.000409
MYOHC1-2	0	0	0	0
MYOHC2a	0.000366	0.000698	0.000755	0.000818
MYOHC2b	0	0	0	0
MYOLCk	0.000366	0.000698	0.000755	0.000818
MYOLCp	0.000366	0.000698	0.000755	0.000818
MYO1-2x	0	0	0	0
MYO2a	0.000183	0.000349	0.000377	0.000409
MYO2x	0	0	0	0
MYO2b	0	0	0	0
TMN1	0	0	0	0
TMN2	5.23E-05	9.98E-05	1.08E-04	0.000117

DIM-TMN1	0	0	0	0
DIM-TMN2	2.61E-05	4.99E-05	5.39E-05	5.84E-05
TPNC1	0	0	0	0
TPNC2	2.61E-05	4.99E-05	5.39E-05	5.84E-05
TPNI1	0	0	0	0
TPNI2	2.61E-05	4.99E-05	5.39E-05	5.84E-05
TPNT1	0	0	0	0
TPNT2	2.61E-05	4.99E-05	5.39E-05	5.84E-05
TPN1	0	0	0	0
TPN2	2.61E-05	4.99E-05	5.39E-05	5.84E-05
CONTR-CO	0	0	0	0
CONTR-CO	2.61E-05	4.99E-05	5.39E-05	5.84E-05
CONTR-CO	0	0	0	0
CONTR-CO	0	0	0	0
Str_CONTR	0	0	0	0
Str_CONTR	2.61E-05	4.99E-05	5.39E-05	5.84E-05
Str_CONT-(	0	0	0	0
Str_CONTR	0	0	0	0
CKM	0	0	0	0
Spont	0	0	0	0
Nut_cr	0	0	0	0
ATP2UTP	0	0	0	0
UDP2ADP	0	0	0	0
PGM1	0	0	0	0
UGP2	0	0	0	0
mGYG	0	0	0	0
mGYS1	0	0	0	0
G6PD	0	0	0	0
PGLS	0	0	0	0
PGD	0	0	0	0
PGD	0	0	0	0
RPE	0	0	0.00E+00	0
RPIA	0	0	0.00E+00	0
TKT1	0	0	0.00E+00	0
TKT2	0	0	0.00E+00	0
TALDO	0	0	0.00E+00	0
RBSK	0	0	0	0
GCL	0	0	0	0
GSS	0	0	0	0
glutathxpo	0	0	0	0
GSR	0	0	0	0
GSR	0	0	0	0
GlutathLea	0	0	0	0
GlutathLea	0	0	0	0
Recycle_na	0	0	0	0
fa-c6 leak	0	0	0	0
acetyLeak(	0	0	0	0

4	5	6	7	
**Arg-Ile-Met-Phe**	**Arg-Ile-Leu-Met-Phe**	**Arg-Ile-Leu-Lys-Met-Pl**	**Arg-Ile-Leu-Lys-Met-Pl**	Supp-Contr
0	0	0	4.201629	4.201629
0	0	0	-0.15432	-0.15432
0	0	0	-0.15432	-0.15432
0	0	0	0	0
0	0	0	0	0
0	0	0	-0.125	-0.125
0	0	0	0	0
0	0	0	0	0
0	0	0	0	0
0	0	0	0	0
0	0	0	0	0
0	0	0	0	0
0	0	0	0	0
0	0	0	0	0
0	0	0	0	0
0	0	0	0	0
0	0	0	0	0
0	0	0	0	0
0	0	0	0	0
0	0	0	0	0
0	0	0	0.266977	0.266977
0	0	0	0	0
0	0	0	0	0
-1.06723	-1.09651	-1.29808	-2.82494	-2.44734
0.661522	0.683949	0.838371	1.875909	1.635311
7.265351	7.472777	8.901019	19.5801	16.95925
0	0	0	0.421299	0.421299
-0.17953	-0.19115	-0.27115	-0.75033	-0.75033
0	0	0	0	0
0	0	0	0	0
0	0	0	0	0
0	0	0	0	0
0	0	0	0	0
0	0	0	0	0
0	0	0	0	0
0	0	0	0.154322	0.154322
0	0	0	0	0
0	0	0	0	0
0	0	0	0.125	0.125
0	0	0	0	0
0	0	0	0	0
0	0	0	0	0
0	0	0	0	0
0	0	0	0	0
0	0	0	0	0

0	0	0	0	0
0	0	0	0	0
0	0	0	0	0
0	0	0	0	0
0	0	0	0	0
0	0	0	0	0
0	0	0	0	0
0	0	0	0	0
0	0	0	0.266977	0.266977
0	0	0	0	0
0	0	0	0.154322	0.154322
0	0	0	0.154322	0.154322
0	0	0	0.154322	0.154322
0	0	0	0	0
0	0	0	0.154322	0.154322
0	0	0	-0.11266	-0.11266
0	0	0	0.266977	0.266977
0	0	0	0.266977	0.266977
-0.0436	-0.03977	-0.0134	0.167078	0.222426
-0.0436	-0.03977	-0.0134	0.167078	0.222426
0	0	0	0.167078	0.130376
0	0	0	0	0
0	0	0	0	0
0.043605	0.039775	0.013402	0	-0.09205
0	0	0	0	0
0.043605	0.039775	0.013402	0.221636	0.166288
0.08721	0.07955	0.026804	0.221636	0.074238
0	0	0	0.041667	0.041667
0.043605	0.039775	0.013402	0.221636	0.166288
0.302464	0.301654	0.296077	0.596876	0.392979
0	0	0	0	0
0.302464	0.301654	0.296077	0.596876	0.392979
0	0	0	0	0
0	0	0	0	0
0.179529	0.191148	0.271147	0.750335	0.750335
0.179529	0.191148	0.271147	0.750335	0.750335
0	0	0	0	0
0.179529	0.191148	0.271147	0.750335	0.750335
0.258859	0.261879	0.282674	0.277777	0.129229
0.438388	0.453027	0.553822	1.125575	0.977026
0.438388	0.453027	0.553822	1.125575	0.977026
0.438388	0.453027	0.553822	1.125575	0.977026
0.481993	0.492802	0.567224	1.347211	1.143314
1.308231	1.342909	1.581691	3.547278	3.067727
0.826237	0.850108	1.014468	2.102604	1.82695
2.134468	2.193017	2.596159	5.649882	4.894678
1.067234	1.096509	1.298079	2.824941	2.447339

5.577167	5.728943	6.774009	14.84704	12.85708
2.950697	3.029548	3.57248	5.688448	4.600844
0	0	0	0.125	0.125
0	0	0	0	0
0	0	0	0	0
0	0	0	0	0
0	0	0	0	0
0	0	0	0	0
0	0	0	0	0
0	0	0	0	0
0	0	0	0	0
0	0	0	0	0
0	0	0	0	0
0	0	0	0	0
0	0	0	0	0
0	0	0	0	0
0	0	0	0	0
0	0	0	0	0
0	0	0	0	0
0	0	0	0	0
0	0	0	0	0
0	0	0	0	0
0	0	0	0	0
0	0	0	0	0
0	0	0	0	0
0	0	0	0	0
0	0	0	0	0
0	0	0	0	0
0	0	0	0	0
0	0	0	0	0
0	0	0	0	0
0	0	0	0	0
0	0	0	0	0
0	0	0	0	0
0	0	0	0	0
0	0	0	0	0
0	0	0	0	0
0	0	0	0	0
0	0	0	0	0
0	0	0	0	0
0	0	0	0	0
0	0	0	0	0
0	0	0	0	0
0	0	0	0.041667	0.041667
0	0	0	0.041667	0.041667
0	0	0	0.041667	0.041667
0	0	0	0.041667	0.041667

0	0	0	0.041667	0.041667
0	0	0	0	0
0	0	0	0	0
0	0	0	0	0
0	0	0	0	0
0	0	0	0	0
0	0	0	0	0
0	0	0	0	0
0	0	0	0	0
0	0	0	0	0
0	0	0	0	0
0	0	0	0	0
0	0	0	0	0
0	0	0	0	0
0	0	0	0	0
0	0	0	0	0
0	0	0	0	0
0	0	0	0	0
0	0	0	0	0
0	0	0	0	0
0	0	0	0	0
0	0	0	0	0
0	0	0	0	0
0	0	0	0	0
0	0	0	0	0
0	0	0	0	0
0	0	0	0	0
0	0	0	0	0
0	0	0	0	0
0	0	0	0	0
0	0	0	0	0
0	0	0	0	0
0	0	0	0	0
0	0	0	0	0
0	0	0	0	0
0	0	0	0	0
0	0	0	0	0
0	0	0	0	0
0	0	0	0	0
0	0	0	0	0
0	0	0	0	0
0	0	0	0	0
0	0	0	0	0
0	0	0	0	0
0	0	0	0	0
0	0	0	0	0
0	0	0	0	0

0	0	0	0	0
0	0	0	0	0
0	0	0	0	0
0	0	0	0	0
0	0	0	0	0
0	0	0	0	0
0	0	0	0	0
0	0	0	0	0
0	0	0	0	0
-0.14787	-0.15177	-0.17857	-0.27685	-0.22111
-0.05096	-0.0523	-0.06153	-0.0954	-0.07619
-0.33142	-0.34704	-0.45457	-1.03471	-0.97745
-0.271	-0.27813	-0.32725	-0.50736	-0.40521
-0.28646	-0.294	-0.34592	-0.5363	-0.42832
-0.07959	-0.08168	-0.09611	-0.149	-0.119
-0.09449	-0.09698	-0.11411	-0.28048	-0.24486
-0.15072	-0.15468	-0.182	-0.28217	-0.22536
-0.01574	-0.01615	-0.019	-0.02946	-0.02353
-0.13436	-0.13789	-0.16224	-0.349	-0.29836
-0.309	-0.309	-0.309	-0.309	0
-0.045	-0.045	-0.045	-0.045	-0.0034
0	0	0	-0.067	-0.067
-0.03272	-0.03358	-0.03951	-0.06126	-0.04893
-0.152	-0.152	-0.152	-0.152	0
-0.23106	-0.23833	-0.28836	-0.47183	-0.40938
-0.15009	-0.15404	-0.18125	-0.281	-0.22442
-0.299	-0.299	-0.299	-0.299	-0.13771
-0.10323	-0.11093	-0.16391	-0.175	-0.175
-0.05532	-0.05678	-0.06681	0	0.020854
0.179529	0.191148	0.271147	0.847798	0.847798
0	0	0	0.097463	0.097463
0	0	0	0.097463	0.097463
0	0	0	0.097463	0.097463
0	0	0	0.097463	0.097463
0	0	0	0.097463	0.097463
0	0	0	0.097463	0.097463
0	0	0	0.097463	0.097463
0	0	0	0.097463	0.097463
0	0	0	0.097463	0.097463
0.179529	0.191148	0.271147	0.847798	0.847798
0.179529	0.191148	0.271147	0.847798	0.847798
0.179529	0.191148	0.271147	0.847798	0.847798
0.179529	0.191148	0.271147	0.750335	0.750335
0.179529	0.191148	0.271147	0.750335	0.750335
0.179529	0.191148	0.271147	0.750335	0.750335
0.179529	0.191148	0.271147	0.750335	0.750335
0.179529	0.191148	0.271147	0.750335	0.750335

0.179529	0.191148	0.271147	0.750335	0.750335
0.179529	0.191148	0.271147	0.750335	0.750335
0	0	0	0	0
0	0	0	0	0
0	0	0	0	0
0	0	0	0	0
0	0	0	0	0
0	0	0	0	0
0	0	0	0	0
0	0	0	0	0
0	0	0	0	0
0	0	0	0	0
0	0	0	0	0
0	0	0	0	0
0	0	0	0	0
0	0	0	0.167078	0.130376
0.054708	0.048015	0.001927	0	-0.24985
0	0	0	0	0
0	0	0	0	0
0	0	0	0	0
0	0	0	0	0
0	0	0	0	0
0	0	0	0	0
0	0	0	0	0
0	0	0	0	0
0	0	0	0	0
0	0	0	0	0
0	0	0	0	0
0	0	0	0	0
0.20832	0.205933	0.189499	0.129232	0.002126
0.387849	0.397081	0.460646	0.879567	0.752461
0.20832	0.205933	0.189499	0.129232	0.002126
0	0	0	0	0
0	0	0	0	0
0	0	0	0	0
0	0	0	0	0
0	0	0	0	0
0	0	0	0	0
0	0	0	0	0
0	0	0	0	0
0	0	0	0	0
0	0	0	0	0
0	0	0	0	0
0	0	0	0	0
0	0	0	0	0
0	0	0	0	0
0	0	0	0	0

0	0	0	0	0
0	0	0	0	0
0.065369	0.068274	0.088277	0.161629	0.161629
0.043605	0.039775	0.013402	0.0999	0.044551
0.043605	0.039775	0.013402	0.0999	0.044551
0.043605	0.039775	0.013402	0.0999	0.044551
0	0	0	0	0
0	0	0	0	0
0	0	0	0	0
0.179529	0.191148	0.271147	0.750335	0.750335
0	0	0	0.103574	0.103574
0	0	0	0.103574	0.103574
0	0	0	0.103574	0.103574
0.387849	0.397081	0.460646	0.97703	0.849924
0	0	0	0	0
0.055323	0.056779	0.066806	0.103574	0.08272
0.015737	0.016152	0.019004	0.029463	0.023531
0.150717	0.154684	0.182	0.282168	0.225356
0.271	0.278133	0.327248	0.507358	0.405206
0.151896	0.155894	0.183423	0.284375	0.227118
0.28646	0.294	0.345917	0.536301	0.428322
0.254292	0.260985	0.307073	0.476078	0.380224
0.134356	0.137893	0.162243	0.251537	0.200893
0.079588	0.081682	0.096107	0.149001	0.119001
0.146835	0.1507	0.177312	0.2749	0.219551
0.149885	0.153831	0.180995	0.280611	0.224112
0.150093	0.154044	0.181247	0.281	0.224423
0.09068	0.093067	0.109501	0.169768	0.135587
0.032722	0.033584	0.039514	0.061262	0.048927
0.346359	0.355475	0.418248	0.648442	0.517884
0.165692	0.170053	0.200083	0.310203	0.247747
0.147875	0.151767	0.178568	0.276847	0.221106
0.094493	0.09698	0.114106	0.176907	0.141288
0.050955	0.052297	0.061532	0.095397	0.07619
0.110369	0.113274	0.133277	0.206629	0.165026
0.000485	0.000498	0.000586	0.000909	0.000726
0	0	0	0	0
0.000971	0.000996	0.001172	0.001817	0.001451
0	0	0	0	0
0.000971	0.000996	0.001172	0.001817	0.001451
0.000971	0.000996	0.001172	0.001817	0.001451
0	0	0	0	0
0.000485	0.000498	0.000586	0.000909	0.000726
0	0	0	0	0
0	0	0	0	0
0	0	0	0	0
0.000139	0.000142	0.000167	0.00026	0.000207

0	0	0	0	0
6.93E-05	7.12E-05	8.37E-05	0.00013	0.000104
0	0	0	0	0
6.93E-05	7.12E-05	8.37E-05	0.00013	0.000104
0	0	0	0	0
6.93E-05	7.12E-05	8.37E-05	0.00013	0.000104
0	0	0	0	0
6.93E-05	7.12E-05	8.37E-05	0.00013	0.000104
0	0	0	0	0
6.93E-05	7.12E-05	8.37E-05	0.00013	0.000104
0	0	0	0	0
6.93E-05	7.12E-05	8.37E-05	0.00013	0.000104
0	0	0	0	0
0	0	0	0	0
0	0	0	0	0
6.93E-05	7.12E-05	8.37E-05	0.00013	0.000104
0	0	0	0	0
0	0	0	0	0
0	0	0	0	0
0	0	0	0	0
0	0	0	0	0
0	0	0	0	0
0	0	0	0	0
0	0	0	0	0
0	0	0	0	0
0	0	0	0	0
0	0	0	0	0
0	0	0	0	0
0	0	0	0	0
0	0	0	0	0
0	0	0	0	0
0	0	0	0	0
0	0	0	0	0
0	0	0	0	0
0	0	0	0	0
0	0	0	0	0
0	0	0	0	0
0	0	0	0	0
0	0	0	0	0
0	0	0	0	0
0	0	0	0	0
0	0	0	0	0
0	0	0	0	0
0	0	0	0	0
0	0	0	0	0
0	0	0	0	0
0	0	0	0	0

ol

	actin	myoHC1-2	myoHC2a	myoHC2b	myoLCk	myoLCp	tmn1	tmn2
arg	18	104	106	100	24	7	14	20
his	9	37	36	40	10	1	2	2
ile	30	103	104	94	24	10	12	12
leu	26	191	200	194	50	9	34	28
lys	19	208	209	212	52	16	38	25
met	17	55	46	56	18	7	8	5
phe	12	56	58	61	20	12	1	1
thr	27	101	98	101	30	11	8	11
trp	4	9	9	9	4	1	0	0
val	21	84	84	86	31	10	7	10
ala	29	171	172	168	56	12	38	28
asn	12	76	75	77	24	7	7	6
asp	22	98	93	94	30	15	19	14
cys	6	17	18	17	10	2	1	3
glu	28	261	265	263	48	15	61	54
gln	11	130	131	127	23	7	15	15
gly	28	72	77	73	48	13	3	4
pro	19	31	30	32	47	6	0	0
ser	23	94	92	97	39	6	11	7
tyr	16	41	38	38	8	2	6	3

tpnc1	tpnc2	tpni1	tpni2	tpnt1	tpnt2	Type 1
4	7	19	17	29	25	2096
0	1	4	5	5	4	748
9	10	4	5	10	10	2175
13	9	22	17	19	19	3804
13	9	24	23	32	39	4142
11	11	8	10	7	4	1281
9	10	2	3	6	4	1335
7	6	6	4	4	7	2210
0	0	2	1	3	2	229
8	7	13	8	11	6	1943
7	12	15	13	20	24	3667
5	3	3	5	3	4	1607
23	19	10	11	12	18	2239
2	1	3	3	1	0	456
23	26	22	25	54	49	4953
5	5	4	8	16	16	2372
12	13	6	7	12	9	2094
2	2	7	5	18	6	1336
5	7	11	10	11	8	2156
3	2	2	2	5	4	848

Type 2a	Type 2x	Type 2b
2133	2105	2049
735	749	791
2191	2177	2051
3909	3783	3825
4132	4118	4174
1148	1274	1288
1363	1335	1405
2174	2216	2216
227	227	227
1938	1938	1966
3668	3654	3612
1592	1606	1620
2162	2232	2176
472	458	458
4996	4940	4968
2390	2376	2334
2165	2095	2109
1308	1322	1336
2118	2146	2188
798	840	798

**AA**	**Available (flux)**	Normalized to 1: available/control flux
**ARG**	0.08	3061.381
**HIS**	0.082	3137.915
**ILE**	0.062	2372.57
**LEU**	0.123	4706.873
**LYS**	0.188	7194.245
**MET**	0.025	956.6815
**PHE**	0.057	2181.234
**THR**	0.14	5357.416
**TRP**	0.044	1683.759
**VAL**	0.233	8916.271
**ALA**	0.333	12743
**ASN**	0.041	1568.958
**ASP**	0.003	114.8018
**CYS**	0.052	1989.897
**GLU**	0.024	918.4142
**GLN**	0.586	22424.61
**GLY**	0.23	8801.469
**PRO**	0.168	6428.899
**SER**	0.114	4362.467
**TYR**	0.059	2257.768

mmol

6.02E+23

Initial Orde	Essentiality	CE		Needed	Available	Difference	Fed	Actual
6	1	1	**MET**	1148	957	-191	1	1
3	1	1	**ILE**	2191	2373	182	2	4
4	1	1	**LEU**	3909	4707	798	3	5
7	1	1	**PHE**	1363	2181	818	4	2
9	1	1	**TRP**	227	1684	1457	5	n/a
5	1	1	**LYS**	4132	7194	3062	6	6
8	1	1	**THR**	2174	5357	3183	7	7
10	1	1	**VAL**	1938	8916	6978	8	n/a
1	1	1	**ARG**	2133	3061	928	9	3
2	1	1	**HIS**	735	3138	2403	10	n/a
15	3	2	**GLU**	4996	918	-4078	11	n/a
13	3	2	**ASP**	2162	115	-2047	12	n/a
12	3	2	**ASN**	1592	1569	-23	13	n/a
20	3	2	**TYR**	798	2258	1460	14	n/a
14	3	2	**CYS**	472	1990	1518	15	n/a
19	3	2	**SER**	2118	4362	2244	16	n/a
18	3	2	**PRO**	1308	6429	5121	17	n/a
17	3	2	**GLY**	2165	8801	6636	18	n/a
11	3	2	**ALA**	3668	12743	9075	19	n/a
16	3	2	**GLN**	2390	22425	20035	20	n/a

Essentiality	Expect on I	Basal
e	1	3
e	2	5
e	3	8
e	4	6
e	5	9
e	6	15
e	7	14
e	8	18
ce	9	7
ce	10	12
ne	11	1
ne	12	2
ne	13	4
ne	14	10
ne	15	11
ne	16	13
ne	17	16
ne	18	17
ne	19	19
ne	20	20
