# Supplementary material for: To Supplement or Not to Supplement: A Metabolic Network Framework for Human Nutritional Supplements
Source: PLoS One. 2013 Aug 5;8(8):e68751. doi: 10.1371/journal.pone.0068751 (PMC3740736; doi:10.1371/journal.pone.0068751)
Supplement: Table S1 — Table of the contractile protein complexes in different types of muscle tissue used for the model. (PDF) [file pone.0068751.s007.pdf]

Table 1 Table of the contractile protein complexes in different types of muscle tissue used for the model.

Muscle Type	Protein	GI (from NCBI)
Type 1	Actin	4501881
	Myosin Heavy Chain type 1/2x	115527082
	Myosin Light Chain Kinase	14993776
	Myosin Light Chain Phosph.	28372499
	Tropomyosin type 1	114155140
	Troponin C type 1	4507615
	Troponin I type 1	56682969
	Troponin T type 1	187173288
Type 2a	Actin	4501881
	Myosin Heavy Chain type 2a	153792663
	Myosin Light Chain Kinase	14993776
	Myosin Light Chain Phosph.	28372499
	Tropomyosin type 2	114155144
	Troponin C type 2	4507617
	Troponin I type 2	4507621
	Troponin T type 2	5803203
Type 2x	Actin	4501881
	Myosin Heavy Chain type 1/2x	115527082
	Myosin Light Chain Kinase	14993776
	Myosin Light Chain Phosph.	28372499
	Tropomyosin type 2	114155144
	Troponin C type 2	4507617
	Troponin I type 2	4507621
	Troponin T type 2	5803203
Type 2b	Actin	4501881
	Myosin Heavy Chain type 1/2x	110611903
	Myosin Light Chain Kinase	14993776
	Myosin Light Chain Phosph.	28372499
	Tropomyosin type 2	114155144
	Troponin C type 2	4507617
	Troponin I type 2	4507621
	Troponin T type 2	5803203
