# Supplementary material for: To Supplement or Not to Supplement: A Metabolic Network Framework for Human Nutritional Supplements
Source: PLoS One. 2013 Aug 5;8(8):e68751. doi: 10.1371/journal.pone.0068751 (PMC3740736; doi:10.1371/journal.pone.0068751)
Supplement: Table S2 — Amino Acids Needed Compared to the Amino Acids Available to produce one contractile protein complex in Type 2a. (PDF) [file pone.0068751.s008.pdf]

Table S2 Amino Acids Needed Compared to the Amino Acids Available to produce one contractile protein complex in Type 2a

- ce – conditionally essential
- E – essential
- ne – non-essential

Table S2A Fasting state ranked by difference

AA	Needed	Available	Difference	Rank	Actual Supplementation Effect	AA Essentiality
GLU	4996	1102	-3894	1	n/a	ne
ASP	2162	138	-2024	2	n/a	ne
MET	1148	1148	0	3	1	e
ASN	1592	1883	291	4	n/a	ne
ILE	2191	2847	656	5	2	e
PHE	1363	2617	1254	6	5	e
ARG	2133	3674	1541	7	4	ce
LEU	3909	5648	1739	8	3	e
TRP	227	2020	1793	9	n/a	e
TYR	798	2709	1911	10	n/a	ne
CYS	472	2388	1916	11	n/a	ne
HIS	735	3765	3030	12	n/a	ce
SER	2118	5235	3117	13	n/a	ne
THR	2174	6429	4255	14	7	e
LYS	4132	8633	4501	15	6	e
PRO	1308	7715	6407	16	n/a	ne
GLY	2165	10562	8397	17	n/a	ne
VAL	1938	10699	8761	18	n/a	e
ALA	3668	15291	11623	19	n/a	ne
GLN	2390	26909	24519	20	n/a	ne

Table S2B Fasting state ranked by essentiality (ce and e considered essential) and then by difference

AA	Needed	Available	Difference	Rank	Actual Supplementation Effect	AA Essentiality
MET	1148	1148	0	1	1	e
ILE	2191	2847	656	2	2	e
PHE	1363	2617	1254	3	5	e
ARG	2133	3674	1541	4	4	ce
LEU	3909	5648	1739	5	3	e
TRP	227	2020	1793	6	n/a	e
HIS	735	3765	3030	7	n/a	ce

THR	2174	6429	4255	8	7	e
LYS	4132	8633	4501	9	6	e
VAL	1938	10699	8761	10	n/a	e
GLU	4996	1102	-3894	11	n/a	ne
ASP	2162	138	-2024	12	n/a	ne
ASN	1592	1883	291	13	n/a	ne
TYR	798	2709	1911	14	n/a	ne
CYS	472	2388	1916	15	n/a	ne
SER	2118	5235	3117	16	n/a	ne
PRO	1308	7715	6407	17	n/a	ne
GLY	2165	10562	8397	18	n/a	ne
ALA	3668	15291	11623	19	n/a	ne
GLN	2390	26909	24519	20	n/a	ne

Table S2C Postabsorptive state ranked by difference

AA	Needed	Available	Difference	Rank	Actual Supplementation Effect	AA Essentiality
GLU	4996	918	-4078	1	n/a	ne
ASP	2162	115	-2047	2	n/a	ne
MET	1148	957	-191	3	1	e
ASN	1592	1569	-23	4	n/a	ne
ILE	2191	2373	182	5	4	e
LEU	3909	4707	798	6	5	e
PHE	1363	2181	818	7	2	e
ARG	2133	3061	928	8	3	ce
TRP	227	1684	1457	9	n/a	e
TYR	798	2258	1460	10	n/a	ne
CYS	472	1990	1518	11	n/a	ne
SER	2118	4362	2244	12	n/a	ne
HIS	735	3138	2403	13	n/a	ce
LYS	4132	7194	3062	14	6	e
THR	2174	5357	3183	15	7	e
PRO	1308	6429	5121	16	n/a	ne
GLY	2165	8801	6636	17	n/a	ne
VAL	1938	8916	6978	18	n/a	e
ALA	3668	12743	9075	19	n/a	ne
GLN	2390	22425	20035	20	n/a	ne

Table S2D Postabsorptive state ranked by essentiality (ce and e considered essential) and then by difference

AA	Needed	Available	Difference	Rank	Actual Supplementation Effect	AA Essentiality
MET	1148	957	-191	1	1	e
ILE	2191	2373	182	2	4	e
LEU	3909	4707	798	3	5	e
PHE	1363	2181	818	4	2	e
ARG	2133	3061	928	5	3	ce
TRP	227	1684	1457	6	n/a	e
HIS	735	3138	2403	7	n/a	ce
LYS	4132	7194	3062	8	6	e
THR	2174	5357	3183	9	7	e
VAL	1938	8916	6978	10	n/a	e
GLU	4996	918	-4078	11	n/a	ne
ASP	2162	115	-2047	12	n/a	ne
ASN	1592	1569	-23	13	n/a	ne
TYR	798	2258	1460	14	n/a	ne
CYS	472	1990	1518	15	n/a	ne
SER	2118	4362	2244	16	n/a	ne
PRO	1308	6429	5121	17	n/a	ne
GLY	2165	8801	6636	18	n/a	ne
ALA	3668	12743	9075	19	n/a	ne
GLN	2390	22425	20035	20	n/a	ne
